# A Spatiotemporal Analysis of Brazilian Science from the Perspective of Researchers’ Career Trajectories

**DOI:** 10.1371/journal.pone.0141528

**Published:** 2015-10-29

**Authors:** Caio Alves Furtado, Clodoveu A. Davis, Marcos André Gonçalves, Jussara Marques de Almeida

**Affiliations:** Computer Science Department, Universidade Federal de Minas Gerais, Belo Horizonte, Brazil; VU University Amsterdam, NETHERLANDS

## Abstract

The growth of Brazilian scientific production in recent years is remarkable, which motivates an investigation on the factors, inside and outside the country, that helped shape this wealthy research environment. This article provides a thorough analysis of the education of researchers that constitute the main Brazilian research groups, using data on about 6,000 researchers involved in the country’s National Institutes of Science and Technology (INCT) initiative. Data on the steps taken by each researcher in her education, from the bachelor’s degree to doctorate, including a possible postdoctoral experience, and employment, are extracted from an official curriculum vitae repository. The location and the time at which each career step occurred define spatiotemporal career trajectories. We then analyze such trajectories considering additional data, including the area of knowledge of the INCTs to which each researcher is associated. We found an increasing prevalence of Brazilian institutions in the education of Brazilian scientists, as the number of doctorates earned abroad is decreasing over time. Postdoctoral stages, on the other hand, often take place in Europe or in the United States. Taking an international postdoctoral position after a full education in Brazil suggests a drive towards seeking higher-level exchange and cooperation with foreign groups in a more advanced career stage. Results also show that Brazilian researchers tend to seek employment in regions that are close to the institutions at which they received their bachelor’s degrees, suggesting low mobility within the country. This study can be instrumental in defining public policies for correcting distortions, and can help other developing countries that aim to improve their national science systems.

## Introduction

The Brazilian scientific production has grown remarkably over the last 15 years [1], reaching an average annual growth rate of 10.7% [2]. With that performance, Brazil has been growing at a pace five times greater than the world average, raising to the 13^th^ place in the international ranking of the most productive nations [2]. The Brazilian academic system, however, is quite young when compared to those of other countries in Europe, North America, Australasia or even South America. The first main Brazilian university was founded only in 1912. The main “ecosystem” of research universities that currently exists in Brazil, with federally funded universities spread over every state of the country, dates from the 1950s [3]. This has historical reasons, mainly related to the main objectives of Portugal in its colonies, which did not include developing strong local educational systems [3]. The recent growth has been impressive, considering that other indicators, such as Brazil’s government spending in R&D and the percentage of the population with tertiary education, show the country is far behind developed countries [4].

Therefore, it is interesting to understand how this young scientific ecosystem evolved to produce such remarkable results in a short period, by analyzing the influences, outside and inside the country, that helped shape this currently wealthy research environment.

In this context, this article provides a thorough analysis of the education of the researchers who compose the main Brazilian research groups, observed from the perspective of their individual *career trajectories*. We analyze the “official” curriculum vitae (CV) of almost 6,000 researchers of the most qualified research groups in Brazil in all areas of knowledge, covering more than 60 years (The Brazilian 2010 Census indicated that about 96,000 PhD graduates lived in the country.). This effort is possible because the CVs of most researchers are available in the Lattes CV system, a central repository kept by the Brazilian government [5]. Data from Lattes are expected to be accurate, as funding sources and promotions in the Brazilian research system require CVs to be always available and up-to-date [6].

In particular, we want to analyze the influence of more scientifically developed regions, such as North America and Europe, on the education of the main Brazilian research groups. We also want to understand the internal influence of the main research institutions in Brazil on the upbringing of the current generation of researchers and on the mobility patterns along their careers. There exists a general idea, which, to our knowledge, mostly derives from anecdotal evidence rather than from scientific studies, that Brazilian researchers tend to stay close to their birthplaces, where their education started. This pattern, supposedly related to social-cultural Latin traditions, differs from the behavior of many other regions in the world that have historically shown high mobility patterns [7, 8]. Restricted mobility could negatively impact the homogeneous development of research institutions and environments throughout the country. We want to investigate whether such tendencies exist, and, if they do, whether this collective behavior has changed over the years.

Specifically, our objectives are: (1) to identify and assess spatial concentrations of researchers in regions of the country, (2) to identify temporal tendencies in the education of researchers and the prevalence (or not) of foreign high education stages, and (3) to characterize frequent spatial patterns for career trajectories of the researchers, including various education stages, a possible postdoctoral experience, and employment. These elements can be instrumental in defining public policies for correcting distortions, and can help other developing countries that aim to improve their national science systems.

The remainder of this article is organized as follows. Section 2 discusses related studies and provides background on the Brazilian science system and on the Lattes CV repository, which serves as a data source for this work. Section 3 describes the dataset used in our study, obtained from the Lattes CVs of a distinguished subset of Brazilian researchers. It also shows how career stages were geographically located based on text contained in the vitae. Section 4 presents our analyses of the collected data, focused on spatial and temporal aspects. Finally, Section 5 presents our conclusions and discusses possible directions for future work.

## Background

### Related work

Previous studies have tackled different aspects of researchers’ careers such as coauthorship patterns [9], research funding [10], job [11] and geographic mobility [12–15]. Several studies on the career trajectories of researchers refer to the so-called *brain drain* phenomenon. Brain drain is an expression used to describe the loss of highly educated individuals living in developing countries through migrations to developed countries [16]. Arenas et al. [13] and Guth & Gill [15] discuss the effects of brain drain in Mexico and East Europe countries respectively. Both studies show that the loss of trained people can cause worrisome outcomes to the scientific development of those countries.

It is easy to see the advantages of brain drain to the country receiving these individuals, an effect called *brain gain* in this perspective. However, could brain drain be anyhow beneficial to the country that loses people? Song’s study about brain drain and brain gain in South Korea [12] shows that Koreans who live abroad or return to Korea after years in another country are helpful in creating international links, due to their enhanced experience. The diaspora, population with ethnic ties living outside their native home, could also contribute to the development of science if they interact with researchers still living in their country of origin [17].

On the other hand, lower mobility can generate other problems, such as academic inbreeding. Indeed, prior studies have observed that PhD holders working in the same institution at which they were trained tend to be less productive, have a smaller *h-index* and coordinate fewer projects [18]. Such behavior is undesirable, but common in Latin institutions [19, 20].

In this article, we perform a detailed analysis of the career trajectories of the researchers of the main research groups in Brazil, covering different areas of knowledge and regions of the country. Our work complements previous studies by focusing on the spatial and temporal distributions of career trajectory stages, from undergraduate studies to employment. In Section 4, we discuss our main findings in light of prior observations, comparing with previous results whenever appropriate.

### The Lattes platform and the Brazilian research groups

The primary source of information of researcher formation and career trajectories used in this work is the Lattes platform. Lattes is a Web-based application created by CNPq (the Brazilian National Council for Scientific and Technological Development) to collect and integrate curriculum vitae (CV) information from the academic community at large. Researchers and students are required to keep their vitae up-to-date in Lattes, as a precondition to applying for grants and other forms of financing [6]. The Lattes platform also carries information on research groups and a directory of institutions. All information in Lattes is publicly available, and currently covers nearly all active Brazilian researchers, groups, and institutions.

As a result, Lattes is a rich database on Brazilian scientific research, taken from the perspective of the individuals involved in it. Financing agencies, science and technology support foundations, as well as CNPq itself, use the platform for management and planning. Moreover, Brazilian researchers also use the platform to trace profiles of the Brazilian science [21–23]. More broadly, the use of CVs as input data for studies on research and science around the world is not new [24, 25], but it offers challenges. For example, it is hard to obtain data for large samples of researchers and the lack of standards in their organization makes their processing more difficult [24, 26]. The Lattes platform helps to mitigate these challenges by being an easily available and standardized source for curriculum vitae [26].

In 2008, CNPq, CAPES (the Brazilian Ministry of Education's organization for graduate courses and curricula) and other regional science foundations created a program to foster and promote Brazilian research groups, called *Institutos Nacionais de Ciência e Tecnologia* (National Institutes of Science and Technology, or INCT). With substantial investment, the program created 101 institutes, covering thematic areas considered strategically important for the country and spreading over every Brazilian region. While the INCT program does not cover every research group in the country, it does include many of the best Brazilian research groups.

InWeb (National Institute of Science and Technology for the Web), one of the institutes created by the INCT program, has the objective of developing models and algorithms to improve the integration of the Web and the society. CiênciaBrasil (*ScienceBrazil*) is one of the research projects conducted within InWeb. The project created a portal in which Lattes vitae data are restructured to configure a research-based social network. Relationships in this social network derive from research collaborations (i.e., coauthorships in publications). The analysis of the resulting network brings forward many aspects of the researchers’ careers and activities, identifying practices, behaviors and characteristics of the scientific production and collaborations. Currently, the portal includes vitae data for all researchers that are members of each INCT’s team.

This work explores spatial and temporal information contained in Lattes vitae data captured in the CiênciaBrasil portal. We characterize the researchers’ spatiotemporal trajectory throughout their careers, including stages such as graduation, graduate studies, and employment (in a university, research institution, or private company), and other career moves. Information on events along each researcher’s career have been collected and organized as described in the next section.

We should note some limitations of our work. First, we analyze CV data only of researchers belonging to the INCTs. All of them were required to have their CVs in the Lattes platform when the INCT projects were submitted to CNPq. We cannot claim that our discoveries extend to *all* Brazilian researchers, nor to particular researchers who do not have a Lattes CV, although we believe the latter to be a minority in the Brazilian academic community. Second, the scientists in our dataset are related to the research area of their INCT groups. Many INCT groups favor multidisciplinarity by aggregating researchers with different expertises and of different areas, despite the general focus of each INCT on a particular theme (e.g., Mathematics, Energy and Biodiversity, Web). Thus, the analysis of research areas in this article reflects the area on which the researcher is currently working, which might differ from that of her undergraduate (or even graduate) degree. Third, since most research members of INCT groups currently live in Brazil, our analyses are biased towards researchers who live in the country.

## Dataset

### Source

As mentioned before, we use data from the Lattes curriculum vitae database. Lattes CVs are available for public consultation online, but downloading large subsets of the database is only allowed under special conditions. Our access to the data is part of the CiênciaBrasil project, through which we intend to provide to CNPq, and to the society at large, a broad view of Brazilian science and scientists as an academic social network, in which relationships derive from scientific cooperation [5]. Currently, CiênciaBrasil data encompasses Lattes CVs for researchers who are involved in the INCT program, in fields that range from health to environmental sciences, and from energy to agriculture.

CNPq grouped the INCTs into 8 major subjects or knowledge areas, considered to be strategically important to the country: agriculture sciences, humanities, environment, energy, engineering and IT, exact sciences, nanotechnology, and health sciences. As mentioned, research groups in INCTs can be multidisciplinary, but each INCT is associated exclusively with one main subject. For instance, the INCT on bioanalytics is associated to exact sciences as a research subject, but may include researchers who work on biology and other related fields.

The dataset currently comprises the CVs of 5,973 unique researchers, who compose the universe of researchers associated with at least one INCT. For this study, we concentrate on spatial and temporal elements of the CV, that is, elements that relate to places and times in the career of each researcher. We intend to learn more about the *career trajectories* of the researchers, that is, where they studied and the locations of the institutions they joined after getting their doctoral degrees (e.g., employment). Combined with CV-declared dates for these steps in each researcher’s career, we put together a view on their spatial and temporal trajectories, to reveal patterns and show tendencies over time.

The Lattes CV contains academic information on the researcher, such as his name, workplace, and a list of her education degrees. Each degree, as well as a possible postdoctoral experience, is associated with an institution, and has the starting and ending years explicitly indicated. We consider the set of institutions attended by a researcher, along with the time periods of attendance, and followed by work places, to be her *career trajectory*. The trajectory is chronologically ordered by the conclusion year of each stage (since we have only the ongoing job, with no “conclusion year” information, the work stage always comes in the last position).

**Fig 1:**
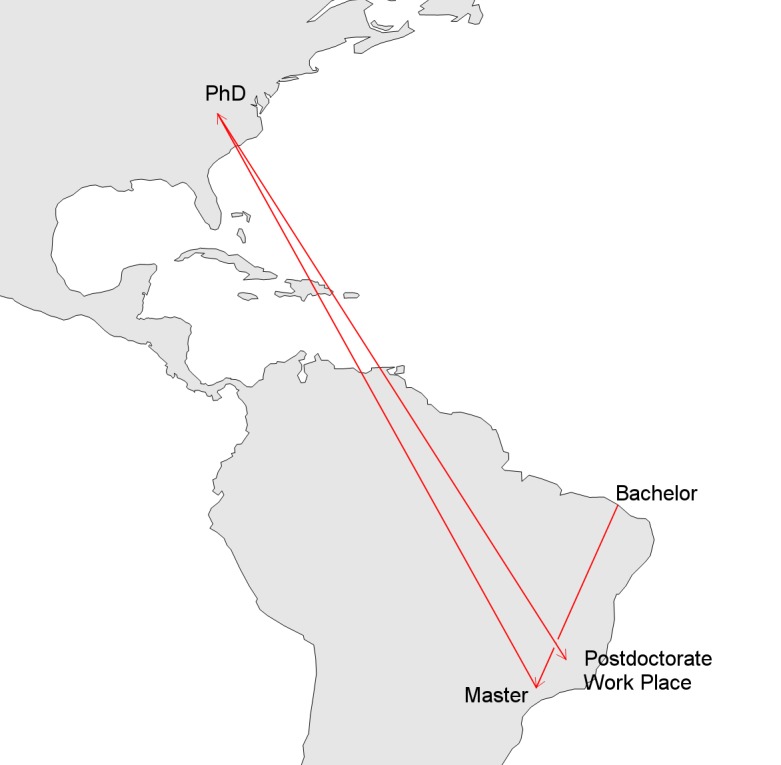
Example of an individual career trajectory Made with Natural Earth. Free vector and raster map data @ naturalearthdata.com.

One possible trajectory is *bachelor* → *master* → *doctorate → post-doctorate* → *work*, as illustrated in Fig 1. The example, created using a visual exploratory analysis tool [27], shows a researcher (in fact, one of the authors of this article) who got his bachelor’s degree in an institution in Ceará state (Brazilian Northeast). He moved to São Paulo (Brazilian Southeast) to study for his master’s degree, and then moved to Virginia state, USA, to pursue his PhD. After completing his PhD, the researcher moved back to Brazil to do his postdoctoral research in Minas Gerais state (Brazilian Southeast), and then got a job in the same institution, remaining in Minas Gerais.

Every researcher in the database has at least one PhD degree entry (although some have not completed the PhD yet). Yet, the number of degrees and the order in which the researcher completed them vary across researchers. For example, a researcher may have two PhD degrees and no postdoctoral experience, or she may jump to a PhD degree after completing the bachelor’s degree, and get a master’s degree afterwards. These moves are indicated by the time interval associated with each degree.

There are two types of institutions in the dataset: *academic institutions*, in which the researchers conducted each stage of their education (including a possible postdoc), and *employment institutions*, where the researchers were working at the time we collected the data. In general, such separation allows us to identify institutions where most researchers enroll for education purposes and those where researchers join as working places. Yet, in our present context, we find no clear division between the two types, since often the academic institutions are the same ones that employ the researchers. This is expected, since all researchers in the database are affiliated to an INCT, and most research groups are based in academic institutions, as very little research is performed in the industry at large in the country. Nevertheless, we opted to keep the separation between academic and employment institutions as a basis for future work involving a broader group of researchers. In our dataset, there are 1,412 unique institutions, 830 of which are exclusively academic, 486 are exclusively employment, and 96 are both academic and employment institutions. As further discussed in Section 4.1, the distribution of researchers between academic and employment institutions follows a long tail pattern: a few institutions formed and employ most of the researchers in our dataset. In particular, the 96 institutions that appear as both academic and employment institutions are responsible for 70% of all degrees granted to the researchers in our dataset. They also employ 78% of these researchers.

We extracted a collection of CVs from the Lattes database following a previously compiled list of INCT research members supplied by CNPq (S1 Table). Data extraction took place between December 2012 and August 2013. Only researchers who are members of at least one INCT and hold at least one PhD degree were considered for this study. As mentioned previously, we assume that Lattes CV data are reasonably accurate, since most researchers must keep their data up-to-date when applying for grants or undergoing any kind of performance evaluation. To gather evidence that supports this assumption, we analyzed the last modification date available in each CV. We found that 94.7% of the profiles had been modified between January 2012 and August 2013, and 76.8% of the profiles had been modified between January 2013 and August 2013. Naturally, typos and other kinds of form-filling mistakes may occur, but overall Lattes CVs are considered to be highly reliable.

### Geocoding

Lattes CVs contain many attributes that indicate geographic locations, either directly or indirectly. Some of them are related to temporal attributes as well. We used geocoding techniques to establish a correspondence between references to places and geographic coordinates. Such techniques intend to determine a location or set of locations given a description of a place. Geocoding used to be associated with the location of postal addresses [28, 29], but we adopt a broader concept, considering as source any kind of textual description [30, 31] and using a wide range of supporting data, such as gazetteers [32] and the application programming interfaces (APIs) of online mapping resources [33].

One of the most important geographic references contained in Lattes CVs derives from the institution of affiliation. We generated a list with unique references to academic institutions, and geocoded this list using the Google Geocoding API. The name of the institution was used as an input address, relying on the API’s capacity for recognizing named landmarks, such as large buildings or facilities. However, we detected variations in the identification of the same institution across CVs from the dataset. We note that, in the Lattes platform, the name of the institution associated with each academic degree, postdoc and employment is provided by the researcher herself as a free-form textual field. Some researchers use only the institution’s name, while others identify a specific city or campus as their work or study places. We chose to use all the information provided by the researcher, including possibly the name of a campus. This is particularly important for institutions that have multiple *campuses*, or maintain units in various cities, as it allows us to get a more accurate location of the researcher. We assumed the main campus location if no further information is provided.

Some studies [34–37] have used the complete address, including the institution’s campus, in their geocoding process, leading to small error rates. Unfortunately, we cannot use the same approach, because, in Lattes, the address is optional for workplace and absent for academic formation institutions. Moreover, CiênciaBrasil’s dataset only includes the name of these institutions. Thus, we can locate a specific campus only if the researcher provided that information along with the institution’s name.

For each unique entry in the list of institutions, we identified the city, state, country, and geographic location associated with it. To validate the geocoding, we manually inspected the location of all PhD granting and employment institutions referenced by five or more researchers. There are 84 educational institutions that meet this condition, corresponding to 80.59% (4,878) of the references in the PhD degree entries, and 106 employment institutions, corresponding to 88.93% (5,207) of the entries. We compared the result with the address declared in the institution’s site and counted the number of incorrect cases.

A total of 13 academic institutions were geocoded incorrectly, corresponding to 594 researchers. Ten of these institutions were geocoded into the right country and state, but in the wrong city. The remaining three institutions, corresponding to 61 of the 594 entries, were geocoded into wrong countries or could not be geocoded at all due to faulty information. A total of 22 employment institutions were geocoded incorrectly, corresponding to 534 researchers. Sixteen of these institutions were geocoded to the right country and state, but in the wrong city. The remaining 6 institutions, corresponding to 62 of the 534 researchers, were geocoded into wrong countries/states or could not be geocoded at all. Most geocoding mistakes occurred in institutions with multiple campuses.

After these verifications, we concluded that institutions that are associated with the large majority of the researchers in our dataset were correctly geocoded. Besides, we manually corrected all errors found in the verification process. A low error rate was expected, since our dataset is composed mostly of universities. Such institutions usually occupy large areas, and therefore are important points of interest for urban mapping in Google Maps, readily identifiable by the geocoding API. Thus, the simple geocoding process we employed was sufficiently accurate.

## Analyses

We begin by providing an exploratory view on the distribution of researchers by institution and by region, within Brazil and abroad. We proceed to analyze the geographical and temporal distributions of researchers’ education degrees across different research areas. Since degrees and work places are geocoded, we analyze the distribution of academic and employment institutions in the world, and within Brazil, to identify typical trajectories. Finally, we analyze the most frequent types of trajectories in the dataset. The following subsections present the results of these analyses.

### Distribution of academic and employment affiliation

**Fig 2:**
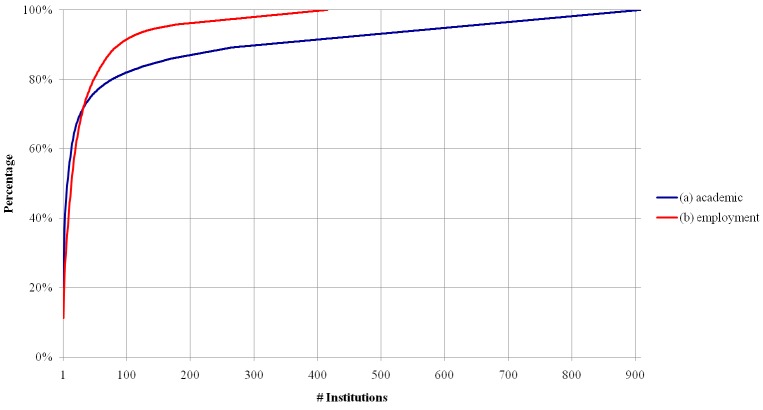
Cumulative distribution of researchers for (a) academic and (b) employment institutions.

We counted the number of researchers associated with each academic institution considering their PhD degrees. Fig 2A shows a cumulative distribution plot. There are 6,053 different entries. Note that researchers who studied in more than one institution (e.g., with a double PhD), which correspond to 1.29% of all researchers, count more than once. As previously mentioned, the distribution displays a long tail behavior, which can be explained by many factors. In particular, we believe that this result primarily reflects two main facts. First, a small number of education degrees are spread through many academic institutions outside Brazil. Second, several academic institutions, such as local colleges, are infrequently associated with researchers, reflecting the tendency, in Brazil, for research to be conducted mostly in major public universities (Source: http://goo.gl/abr5aW in Portuguese). Notice that about 80% of the researchers are concentrated in less than one hundred institutions, which can be considered the main drivers of Brazilian research. Moreover, we also note that Lattes CVs require the indication of the institution’s name, but some researchers mention a department or institute within the institution. For instance, while 1,206 researchers filled out “*Universidade de São Paulo*”, 113 other researchers explicitly mentioned subdivisions of that university (e.g., institutes, campuses, academic units) as their PhD granting institution. Such variations may also contribute, to some extent, to the long tail observed in Fig 2A.

The distribution of employment institutions has a similar behavior, but our data includes a single employment institution per researcher (Fig 2B). Few institutions (34) account for more than 70% (4,199) of the entries. Again, the institutions that employ most researchers correspond to the top Brazilian universities, as observed for the academic institution affiliations. The long tail, in this case, is composed mostly of private companies.

### Distribution of research areas

Table 1 shows the distribution of researchers according to the main subject of the INCTs in which they participate. For each area, it shows the number of researchers (86 researchers are associated to more than one INCT) as well as the earliest and the median year in which these researchers received their PhDs. Health/Medical and Environment are the areas with the largest numbers of researchers, accounting for almost half of the researchers in the dataset. Most of the areas have at least one researcher who received a PhD in the late 1950s or early 1960s, with two exceptions: Agriculture and Humanities. These two areas have younger senior scientists and younger teams in general. On the other hand, the Exact, Health and Engineering/IT INCTs include the most senior researchers. It may seem strange at first that research areas such as Nanotechnology have older senior scientists than other more traditional areas, such as Agriculture and Humanities. This fact reflects the multidisciplinary nature of the INCTs. For example, the researcher in the Nanotechnology INCT who graduated in 1961 has a PhD in Chemistry, and his INCT group, the INAMI (*Nanotecnologia para Marcadores Integrados*), also employs biologists, physicists and engineers.

**Table 1:** Distribution of researchers across INCT areas.

Area	# Researchers	Earliest PhD Year	Median PhD Year
**Agriculture**	535	1972	2000
**Energy**	386	1960	1999
**Engineering/IT**	691	1958	1999
**Environment**	897	1962	2000
**Exact sciences**	585	1958	1995
**Health/Medical**	1,908	1957	1999
**Humanities**	388	1969	2002
**Nanotechnology**	626	1961	1999
**Total**	6,016	1957	1999

**Fig 3:**
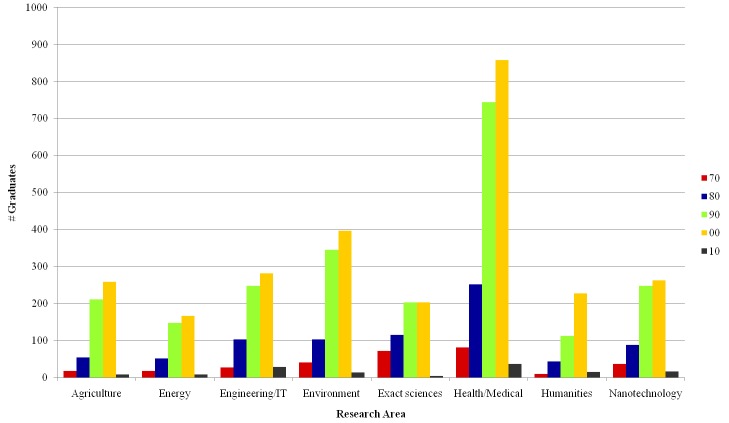
PhD graduations per decade.

Fig 3 shows the distributions of PhD graduations over time for each area. Agriculture, Health/Medical and Engineering/IT show a larger increase in the number of researchers in more recent decades. On the other hand, the rate of growth for Exact sciences is less steep. The strong growth in Health/Medical degrees can be associated with the increase in research related to health issues which are typical of developing and tropical countries (Source: http://goo.gl/vWKQMQ in Portuguese), such as Brazil. INCT themes in this field include dengue fever, tuberculosis, neglected diseases, biomedicine in the semi-arid Northeastern Brazil, and others. There is also a strong strategic drive towards research in that direction, as indicated by the fact that Health/Medical is the largest group of INCT: 37 out of 101 institutes belong to this group. On the other hand, notice that the number of researchers grows in every area, showing that scientific activities in Brazil have increased in the last two decades. Note also that the numbers for the 2010s are not comparable to other time periods, as the decade is far from complete.

#### Master degrees and postdoctoral positions per research area

Having analyzed the PhD stage, in this subsection we focus on other stages in the education of the researchers, looking for subject-specific patterns of career trajectory. Using the Lattes data, we analyze how the numbers of researchers with master degrees and postdoctoral experiences vary across the research areas.

Table 2 contrasts the research groups and the number of researchers with postdocs. The distribution is uneven and, in some areas, such as Exact sciences and Nanotechnology, the fraction of researchers with PhDs who also have postdocs (4^th^ column of Table 2) is much higher than in the other areas. Furthermore, since a researcher may have spent multiple postdoctoral periods in different institutions, there are even more postdoc positions than PhD degrees (each postdoctoral period is counted once) in Exact sciences (6^th^ column). Prior studies that also analyzed the frequency of postdoc experiences among researchers with PhD degrees in different knowledge areas found somewhat different results. For instance, Nerad and Cerny [38] compared the shares of PhDs with postdocs in Mathematics and Biochemistry, finding it to be higher for the latter. Martinelli [39] analyzed the ratio of postdocs among researchers who had recently obtained their PhD degrees, finding it to be higher in Natural Sciences and Chemistry, as opposed to Humanities, Mathematics and Electrical Engineering. Yet, we note that our findings cannot be directly compared to these analyses, since we group researchers based on the area they are currently working on (inferred from the area of the INCT they belong to). As mentioned, though centered on specific themes, INCTs often aggregate researchers with degrees in different areas of knowledge. Other related studies analyzed the distribution of a group of researchers with postdocs across different knowledge areas [40–42]. Our analysis complements these prior efforts as we here focus on the frequency of postdoctoral experiences within groups of researchers in different knowledge areas.

**Table 2:** Masters degrees and postdoctoral positions per research area.

Area	# Researchers with PhD	# Researchers with Postdoc	% Postdoc/PhD	#Postdoc positions	% Postdoc positions/PhD	# MSc	% MSc/PhD
**Agriculture**	526	254	48.29%	348	66.16%	493	93.73%
**Energy**	379	146	38.52%	217	57.26%	342	90.24%
**Engineering/IT**	666	317	47.60%	449	67.42%	643	96.55%
**Environment**	872	350	40.14%	476	54.59%	824	94.50%
**Exact**	585	436	74.53%	750	128.21%	508	86.84%
**Health/Medical**	1,881	1,004	53.38%	1,434	76.24%	1,525	81.07%
**Humanities**	378	162	42.86%	213	56.35%	354	93.65%
**Nanotechnology**	622	405	65.11%	604	97.11%	534	85.85%

Table 2 also shows the distribution of master (MSc, or Master of Science) degrees in each research area. The differences across areas are not as expressive as observed for postdocs. The table shows that most researchers have both PhD and MSc degrees, although the fraction of researchers with a PhD and *without* a MSc degree is larger in the Health/Medical, Nanotechnology, and Exact sciences INCTs (rightmost column of Table 2).

#### Geographic distribution of PhD granting institutions across research areas

**Fig 4:**
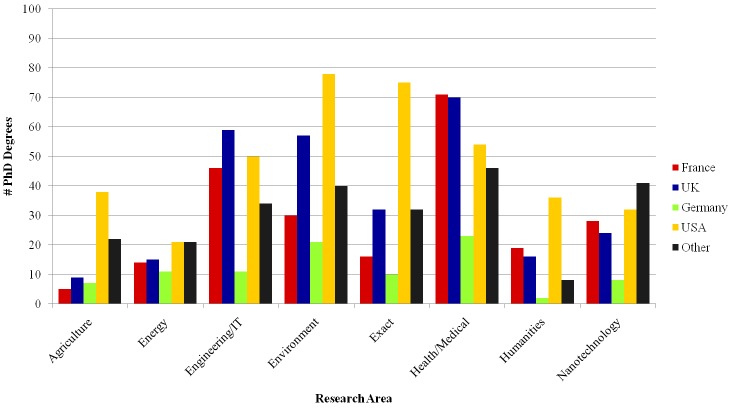
Geographic distribution of PhD degrees per research area (except Brazil).

Researchers involved in the INCTs have obtained their PhD degrees from institutions all around the world. Fig 4 shows the most common countries, besides Brazil (left out for clarity), for each research area. They are France, United Kingdom, United States, and Germany. Around half of the areas (Agriculture, Exact sciences, Humanities and Environment) have a larger share of PhDs from institutions in the USA, while the UK and France graduated proportionally more researchers in Health and Engineering/IT.

**Fig 5:**
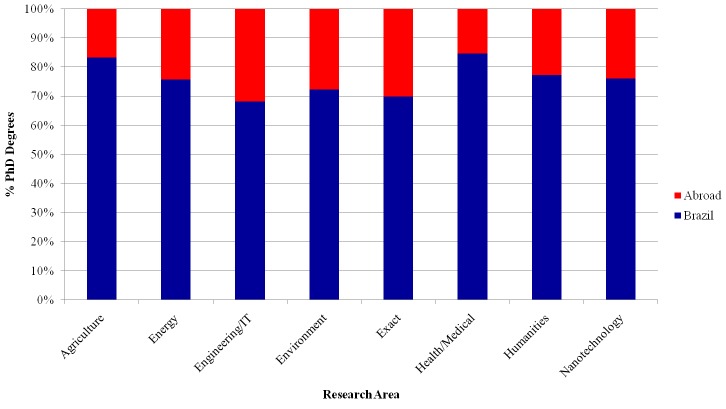
Proportion of Brazilian and international PhD degrees.

Fig 5 shows the proportion of PhD degrees obtained in Brazilian institutions and abroad. While the ratio of degrees from international institutions to degrees granted by Brazilian institutions varies across research areas, Brazilian institutions dominate the PhD granting institutions in all areas. No research area has more than 40% of international graduates, and the smallest ratio is 15% (for Health/Medical). This distribution shows that most of the education of Brazilian researchers is currently done within the country. However, there is a non-negligible fraction of international PhDs, from which international cooperation and joint research projects may arise.

**Fig 6:**
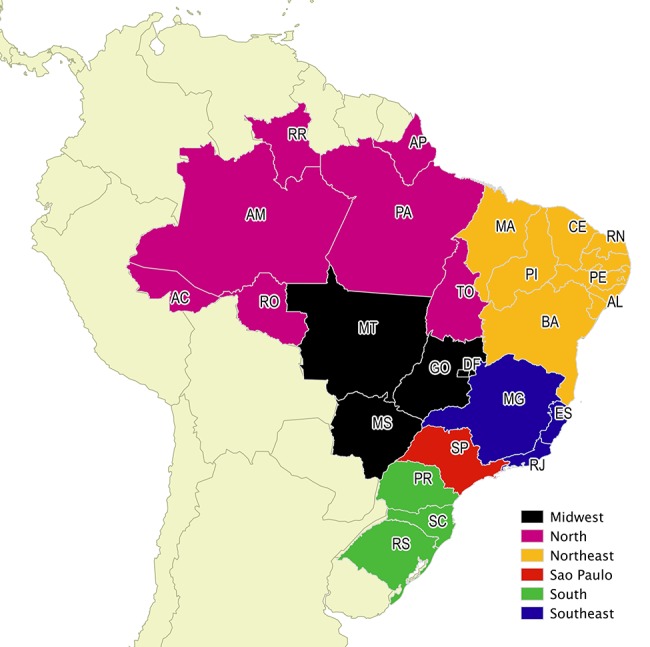
Brazilian regions (São Paulo detached from the Southeast) Made with IBGE map. Free vector map data @ mapas.ibge.gov.br/interativos/arquivos.

We further analyzed the PhD degrees obtained in Brazil by looking at the distribution of institutions across the country. We considered a traditional division of Brazil into regions (North, Northeast, Midwest, Southeast and South), but took São Paulo state, the richest and most populous state in the country, separately from the Southeast region (Fig 6). São Paulo alone is responsible for about half of the researchers in the dataset, although it concentrates around only 22% of the Brazilian population (São Paulo population is 41 million habitants in 2010. Brazil total population in 2010 is about 190.7 million). Since the state’s numbers are expressive, we chose to analyze it separately.

**Fig 7:**
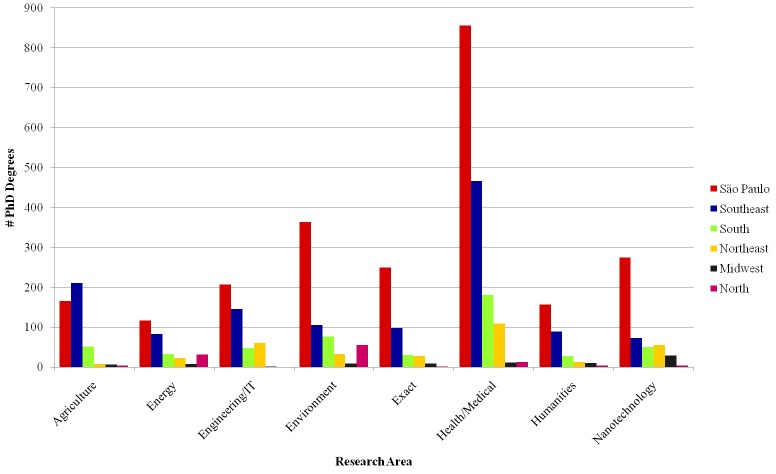
PhD degrees per Brazilian region.

Fig 7 shows the distribution of the number of PhD degrees across Brazilian regions for each research area. Most PhD degrees granted by institutions in the North region, where the Amazon forest is located, are in the Environment and Energy areas. Nanotechnology also accounts for a disproportionately large share of the degrees obtained in the Midwest region, although the absolute number of degrees granted by institutions in that region is low. Other Brazilian regions follow a more regular pattern, with an emphasis on Agriculture sciences in the southeast.

Possible explanations for the patterns observed in Fig 7 are as follows. According to GeoCapes, a georeferenced information system maintained by CAPES, the Brazilian regions sorted in decreasing order of number of graduate programs are Southeast, South, Northeast, Midwest and North. The same order holds for the distributions of PhD degrees for most research areas in Fig 7, with São Paulo state separately standing out from the other southeastern states. Indeed, the growth of programs in the North, Northeast and Midwest is very recent, according to Avelar [43], and has attained an expressive growth of 50% between 2000 and 2010. This might explain why these regions still have a less expressive participation in the education of Brazil’s research community. One exception to the general pattern is the Agriculture area, where São Paulo state comes in second place. This can be explained by the prevalence of a larger number of graduate programs in the state of Minas Gerais (compared to São Paulo). Indeed, one third of the programs located in Minas Gerais (Southeast) are dedicated to agricultural sciences. Another exception is the Environment area, in which the North region shows an expressive number of PhDs, probably due to the proximity to the Amazon region. Regarding Nanotechnology, the larger share of degrees obtained in the Midwest region might be explained by one particularly large INCT in that group which has as theme the nanobiotechnology of the North and Midwest regions.

São Paulo’s domination in most areas can be further explained by the fact that the state houses some of the top Brazilian universities. Three institutions located in São Paulo state and supported by the state government appear in the Top 10 of the 2014 QS University Rankings of Latin American. Most of the other top Brazilian institutions are supported by the federal government. Yet, even though there is at least one federal university per state, those that are more active in research are in the Southeast and South regions, including São Paulo. The same QS ranking have 17 institutions from South and Southeast in their Top 20 of Papers per Faculty, considering Brazilian institutions only

In sum, São Paulo state concentrates most of the researcher education institutions, as some of the main universities in the country are located in that state. However, as we discuss in the next section, the prevalence of São Paulo has been decreasing over time. Other regions are still far behind São Paulo, although some institutions in the Northeast and in the North are important both regionally and as reference centers for specific subjects, such as Environment.

### Spatiotemporal distribution of PhD degrees

**Fig 8:**
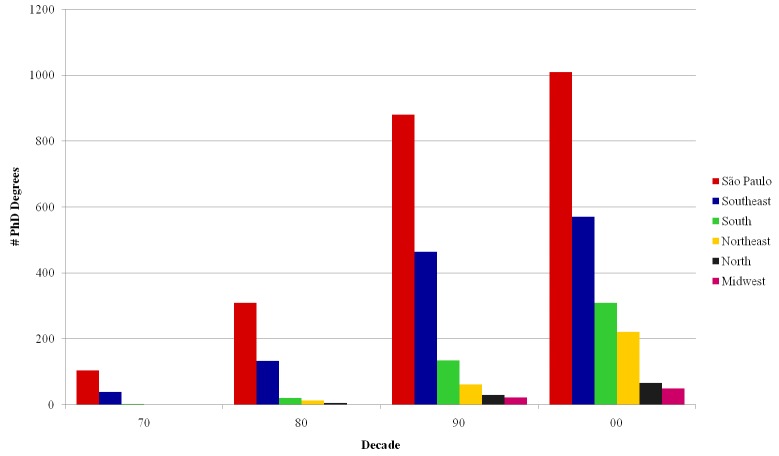
PhD degrees per decade–National.

We now turn to the temporal distribution of PhD degrees across different regions. Focusing first on regions in Brazil, Fig 8 shows that institutions located in São Paulo state have been responsible for a large share of the PhD degrees granted by Brazilian institutions in the last decades. However, their prevalence in the country has been diminishing recently. Considering that the data reflects only INCT researchers, Fig 8 shows that the share of the scientists in the main Brazilian research groups coming from institutions located in other parts of the country has increased over the years. As to the absolute growth in the number of PhD degrees granted by Brazilian institutions, the Fig shows an acceleration in the graduation of PhDs from the 1980s in Southeast institutions, and from the 1990s in South and Northeast institutions. This indicates a tendency towards decentralization as institutions distributed throughout the country become more mature.

**Fig 9:**
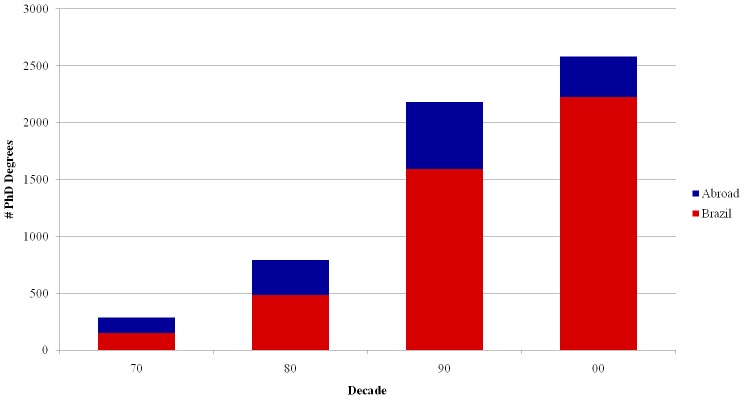
PhD degrees per decade, referred to the year of completion.

Fig 9 shows the distribution of PhD completions per decade, considering both degrees obtained in Brazil and abroad. Most of the INCT researchers graduated between the 1990s and the 2000s. The small numbers of PhDs from the 1970s reflect the coming of age for retirement. We note that the fraction of PhD degrees obtained abroad is decreasing along the years. This fraction was 48% in the 1970s, went down to 27% in the 1990s and to only 14% in the 2000s. These numbers provide additional evidence of the maturing of the Brazilian Science. This observed trend is well aligned with the governmental programs and grants in vogue in these decades, which stimulated study abroad from the 1950s to the 1980s. In parallel, in the 1960s, a law promoted an expansion of the number of graduate programs, in order to employ researchers educated out of the country and to cater for the domestic demand thereon [44, 45].

**Fig 10:**
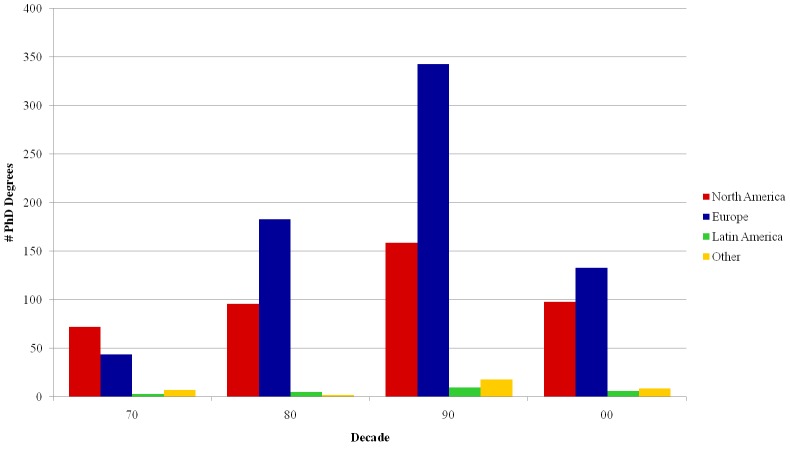
PhD degrees per decade–International distribution

In total, there are 1,225 researchers with PhD degrees obtained abroad. As shown in Fig 10, North America (mostly the USA) has a large participation, but European PhDs have become prevalent since the 1980s. It is interesting to note the small number of researchers who studied in other Latin American countries. One could expect that Brazilians would study in countries where they share similar languages or are geographically close, but this is not the case. Not even Portugal accounts for a large share of the degrees, as only 6 of the 1,225 researchers graduated in that country. A similar fact was also observed in Portugal, where only 4% of the research contracts in the country are awarded by people natural from Portuguese-speaking countries [19].

In summary, CiênciaBrasil data shows that most researchers in INCTs have obtained their PhDs in Brazil, and the share of international PhDs seems to be diminishing over time. International destinations for PhD studies are mainly in Europe, although a large share of those degrees are also obtained in the United States. The distribution of destinations, in Brazil and abroad, varies according to the area of knowledge, as further demonstrated in the next section.

### Origin and destination points in the trajectory

In this section, we compare the initial point of the researcher’s academic trajectory recorded in her CV, i.e., the place where the researcher completed her undergraduate studies, to two other points further ahead in her trajectory, the location of the institution that granted her PhD and the location of the employment institution.

Regarding the initial stage in the academic trajectory, we point out some peculiarities of the Brazilian undergraduate education system. Brazilian student admittance procedure to undergraduate programs in most public universities, which are free of tuition and other fees, typically includes a very competitive selection process. In 2002, admissions to public institutions (including federal and non-federal institutions) corresponded to 16.7% of the total offers. The reported average number of applicants per position in a federal public university was 9.5 in that year. On the other hand, the national average for private institutions was only 1.6 [46]. In 2013, the national average of applicants per position in federal institutions reached 18.7, with institutions located in the Southeast region being the most competitive ones (23.1 candidates per position). The National Institute of Educational Studies and Research (INPE) provides yearly statistical synopsis of Brazilian Higher Education. A file with the complete synopsis can be found in http://portal.inep.gov.br/superior-censosuperior-sinopse (in Portuguese)

Until recently, each university conducted its own selection process. Since the selection occurred at the same time of the year all around the country, most students applied only to few institutions, often those located closer to their home towns (or in their home states). Since 2009, a unified selection system is in place, and universities around the country are increasingly (partially or totally) adhering to the new system. Such unified selection process is expected to promote more mobility, so students would more often seek better institutions, even if they are far away from their home towns. Yet, a survey conducted in 2013 (Source: http://goo.gl/BFLVe in Portuguese) shows that only about 13% of the undergraduate students enroll in universities outside their home states. We conjecture that this percentage was even lower in the previous decades, and thus INCT researchers mostly took their undergraduate courses in their home states. Therefore, we assume that the initial point in their academic trajectories is in the same state where they received their first (bachelor) degrees.

Table 3 shows the distribution of researchers by regions in Brazil and abroad in three of their career stages: undergraduate, PhD, and work. As before, we considered only the first entry in case of multiple degrees. The distributions of undergraduate studies and employment institutions are similar, while there is a proportionally higher concentration of PhD degrees obtained in São Paulo, Europe and North America. This confirms the analysis discussed in the previous section: over the last decades, these were the most sought-after destinations for advanced studies. The final trajectory point, however, suggests a move to an institution that is closer to home, that is, in the state where the undergraduate studies were completed.

**Table 3:** Distribution of researchers per trajectory stage and region.

	Undergraduate		PhD		Employment	
	Absolute	Relative	Absolute	Relative	Absolute	Relative
**Brazil Midwest**	226	3.88%	78	1.33%	381	6.51%
**Brazil Northeast**	840	14.41%	310	5.28%	885	15.12%
**Brazil North**	206	3.53%	106	1.81%	346	5.91%
**Brazil Southeast**	1,601	27.47%	1,24	21.14%	1,562	26.68%
**Brazil São Paulo**	1,557	26.71%	2,335	39.81%	1,671	28.54%
**Brazil South**	922	15.82%	483	8.23%	783	13.37%
**Asia and Oceania**	18	0.31%	37	0.63%	2	0.03%
**Africa**	2	0.03%	2	0.03%	1	0.02%
**Europe**	109	1.87%	703	11.98%	17	0.29%
**Latin America**	138	2.37%	27	0.46%	1	0.02%
**North America**	58	1.00%	429	7.31%	20	0.34%
**Non-located**	152	2.61%	116	1.98%	186	3.18%
**Total**	5,829	100%	5,866	100%	5,855	100%

We note that the concentration of advanced studies in institutions located in São Paulo as well as in other regions is not an effect of population concentration. Table 4 shows a comparison between the number of people with PhD degrees, according to CNPq data available at http://www.memoria.cnpq.br/estatisticas/investimentos/regiao.htm (Table 1.5.8), and the total number of INCT researchers across different regions of the country. We associate the researcher with the region where her work institution is located, since it is the most current information available about the researcher’s location. For comparison purposes, the table also shows the distribution of the population across these regions (rightmost column) as provided by CNPq.

**Table 4:** Distribution of researchers–National and per INCT groups.

	#PhDs Census	% PhD	#PhDs in INCTs	% PhDs in INCTs	% Population (2014)
**Midwest**	7,694	7.96%	381	6.77%	7.5%
**Northeast**	16,773	17.36%	885	15.72%	27.7%
**North**	4,011	4.15%	346	6.15%	8.5%
**Southeast**	23,554	24.37%	1,562	27.75%	20.3%
**São Paulo**	25,270	26.15%	1,671	29.69%	21.7%
**South**	19,336	20.01%	783	13.91%	14.3%
**Total**	96,638	100%	5,628	100%	100%

The overall distribution is similar, but notice that São Paulo and the Southeast together account for more than half of the PhDs in the country, and over 57% of the researchers involved in INCTs, while these regions correspond to 42% of the overall population. Since the INCTs represent the most advanced research in Brazil, a larger concentration of researchers in these regions (with respect to the population) may indicate more mature research institutions and groups, compared to those located elsewhere. The same holds for the South region, which accounts for 14% of the INCT researchers, and a similar fraction of the overall population.

On the other hand, the table shows that the INCT program succeeded in involving scientists from every region, and in a proportion that is similar to their capacities (measured by the number of resident PhDs). The absolute numbers, however, indicate that there is still a long way to go. While there are 0.6 researchers per 1,000 inhabitants in the most developed region of Brazil (Southeast including São Paulo), the ratios in Europe (27 countries) and in the USA are 6 and over 8 researchers per 1,000 inhabitants respectively [7].

We further analyze origin and destination points in the researcher’s trajectories by presenting in Tables 5, 6 and 7 the distributions of researchers per region in two distinct points of their career trajectories. The distribution in Table 5 is computed using the undergraduate degree as the origin and the employment institution as destination, leaving out trajectories with missing information at either end. The concentration in the main diagonal shows a tendency to avoid moving to other parts of the country for a job. People tend to prefer living in the same region where they completed their undergraduate studies. We can see that Brazilian institutions do employ people who graduated in different regions. However, most of them graduated in some institution located in the same region as the institution. Similarly, most researchers who graduated in a region tend to stay there. One interesting exception is the Midwest: while almost 70% of researchers working in the Midwest have not received their undergraduate degrees in the region, about 50% of them are employed there.

**Table 5:** Origin and destination trajectory analysis–from undergraduate to employment institution.

	Destination—Work												
Origin–Bachelor		AA	AF	BMI	BNE	BNO	BSE	BSP	BSU	EU	LA	NA	Total
	**AA**	***0***	0	2	2	1	2	8	1	0	0	0	16
	**AF**	0	***0***	0	1	0	1	0	0	0	0	0	2
	**BMI**	0	0	***113***	11	14	27	41	9	0	0	1	216
	**BNE**	0	0	31	***610***	28	47	70	16	0	0	1	803
	**BNO**	0	0	10	9	***152***	8	11	4	1	1	0	196
	**BSE**	1	0	86	51	31	***1*,*122***	175	53	8	0	6	1,533
	**BSP**	0	0	68	82	35	131	***1*,*076***	70	0	0	8	1,470
	**BSU**	1	0	40	56	41	63	108	***566***	0	0	2	877
	**EU**	0	1	6	10	6	32	30	12	***5***	0	0	102
	**LA**	0	0	9	17	8	31	36	24	1	***0***	0	126
	**NA**	0	0	1	4	6	14	19	6	0	0	***1***	51
	**Total**	2	1	366	853	322	1,478	1,574	761	15	1	19	***5*,*391***

AA: Asia

AF: Africa

BMI: Brazil Midwest

BNE: Brazil Northeast

BSE: Brazil Southeast (except São Paulo)

BSP: São Paulo

BSU: Brazil South

EU: Europe

LA: Latin America

NA: North America.

**Table 6:** Origin and destination trajectory analysis–from Undergratuate to PhD.

	Destination–PhD												
Origin—Undergraduate		AA	AF	BMI	BNE	BNO	BSE	BSP	BSU	EU	LA	NA	Total
	**AA**	***5***	0	1	0	1	2	2	0	2	0	3	16
	**AF**	0	***0***	0	0	0	1	0	0	0	0	0	1
	**BMI**	1	0	***35***	2	3	26	89	6	34	0	21	217
	**BNE**	4	0	6	***254***	6	96	259	16	114	1	42	798
	**BNO**	1	0	7	10	***55***	32	57	9	16	0	7	194
	**BSE**	7	0	11	11	12	***871***	292	22	179	2	146	1,553
	**BSP**	5	1	6	8	13	45	***1*,*213***	8	119	0	94	1,512
	**BSU**	11	1	6	7	8	53	241	***390***	131	1	46	895
	**EU**	0	0	0	0	0	14	19	3	***58***	1	6	101
	**LA**	0	0	1	3	2	30	47	7	7	***22***	12	131
	**NA**	1	0	0	1	1	6	6	3	4	0	***34***	56
	**Total**	1	2	73	296	101	1,176	2,225	464	664	27	411	**5,*440***

AA: Asia

AF: Africa

BMI: Brazil Midwest

BNE: Brazil Northeast

BSE: Brazil Southeast (except São Paulo)

BSP: São Paulo

BSU: Brazil South

EU: Europe

LA: Latin America

NA: North America.

**Table 7:** Origin and destination trajectory analysis–from PhD to employment institution.

	Destination—Work												
Origin—PhD		AA	AF	BMI	BNE	BNO	BSE	BSP	BSU	EU	LA	NA	Total
	**AA**	***0***	0	1	9	1	4	12	5	0	0	1	33
	**AF**	0	***0***	0	1	0	0	1	0	0	0	0	2
	**BMI**	0	0	***44***	5	10	6	2	3	0	0	1	71
	**BNE**	0	0	2	***253***	15	10	10	4	0	0	1	295
	**BNO**	0	0	5	4	***87***	3	2	1	0	0	0	102
	**BSE**	1	0	70	94	41	***866***	65	45	6	0	4	1,192
	**BSP**	0	0	151	273	99	253	***1*,*230***	194	2	1	7	2,210
	**BSU**	1	0	22	29	20	24	23	***340***	0	0	0	459
	**EU**	0	1	51	128	32	186	147	125	***7***	0	2	679
	**LA**	0	0	1	5	1	3	7	9	0	***0***	0	26
	**NA**	0	0	27	47	20	160	115	37	0	0	***2***	408
	**Total**	2	1	374	848	326	1,515	1,614	763	15	1	18	***5*,*476***

AA: Asia

AF: Africa

BMI: Brazil Midwest

BNE: Brazil Northeast

BSE: Brazil Southeast (except São Paulo)

BSP: São Paulo

BSU: Brazil South

EU: Europe

LA: Latin America

NA: North America.

Table 6 compares the locations of the undergraduate and the PhD studies, leaving out trajectories with missing information at either end. Once again, numbers are heavily concentrated along the main diagonal, indicating a similar trend towards seeking a PhD in the same region of the institution that granted the undergraduate degree. A notable exception is the concentration of PhD degrees granted in São Paulo to researchers who obtained their undergraduate degrees in the Brazilian Midwest, Northeast and South, as expected from the results of the previous analyses.

Finally, Table 7 covers the transition from the PhD granting institution to the employment institution. Naturally, given that the dataset contains INCT researchers, most of them, even those who received their PhDs abroad, work at an institution located somewhere in Brazil, mainly in São Paulo and in the Southeast and South regions. Notice also that researchers who received their PhDs in São Paulo are spread across several regions of the country, although most of them work in São Paulo itself.

In summary, analyzing the CVs of INCT researchers, we notice some resistance against moving to other parts of the country. INCT researchers are mostly Brazilians who obtained (most of) their degrees close to home and currently work in the same region. The fraction of researchers who have pursued advanced studies in other regions or abroad is about 32%. The behavior changes from region to region, though São Paulo and Southeast are the only regions where the majority of researchers are originated from the region itself. Midwest, North, Northeast, and South (to a lesser extent) show a temporary migration behavior: we note an expressive fraction of researchers migrating out of these regions from undergraduate to PhD, and then an influx of researchers back to there from PhD to workplace. This observation contrasts sharply with a mobility study regarding European researchers [8], which shows that half of the students who went to the USA for a PhD in Economics took up a job in that country. Out of the other half, one third remained in their home countries, while the other two thirds sought employment in other European countries. We also note that the participation of Brazilians working abroad is small, and it is evidence of the need to encourage the Brazilian scientific diaspora to take a more important role in the Brazilian science [17].

### Internationalization

The set of points in the trajectories of INCT researchers, while concentrated in Brazil, includes an expressive number of international steps. In total, 2,727 researchers have completed at least one degree or a postdoc in a foreign institution. Table 8 shows the percentages of degrees obtained and postdoctoral periods spent in foreign institutions.

**Table 8:** Foreign stages per modality.

	% stages abroad
**Undergraduate**	5.78%
**Master**	6.94%
**PhD**	20.58%
**Postdoctoral periods**	62.46%

The percentage of international degrees increases in higher stages, reaching almost 21% for PhD. Besides, comparing all trajectory points, we find that a postdoc position is the most common career stage spent abroad. Thus, in general, we find that the higher the stage the researcher is in her trajectory, the higher the chance of the researcher doing this stage in some foreign institution.

For example, over 60% of the INCT researchers who have had a postdoctoral research experience have done it in foreign institutions. Moreover, 1,262 researchers (21% of the INCT researchers) have a postdoc as their only career stage abroad. Indeed, only 1,207 PhD degrees were obtained in international institutions, representing about 20% of the number of INCT researchers. A possible explanation for the difference in the shares of PhDs and postdocs abroad would be that Brazilian researchers who conducted most or all of their education in Brazil seek postdoctoral positions as a means to expand their international experience and increase cooperation with foreign research groups. A more detailed analysis of the intensity of the cooperation, measured by the number of publications coauthored with foreign scientists after the postdoctoral experience, could confirm this hypothesis. Publication lists can also be obtained from Lattes CVs, but naturally foreign scientists do not have their CVs in the system. Therefore, we leave this analysis for future work.

Although the increasing demand for international postdoctoral labor in the USA [47, 48] and UK [47] in recent years might help explain (at least partially) the higher share of postdoctoral positions taken abroad, it is not the only factor. As a matter of fact, we observe a much sharper increase in the number of postdoctoral positions in Brazilian institutions in the recent years, compared to postdoctoral positions taken abroad. For instance, 1,016 researchers in our dataset held a postdoctoral position abroad before the year 2000. The same number for the following years is only slightly larger (1,345). On the other hand, while only 216 researchers held postdoctoral positions in Brazil before 2000, this number increased by almost a factor of 5 afterwards (1,036). The internationalization of researchers in the postdoctoral stage seems to be a recurring phenomenon, reflecting old governmental programs that sent scholars abroad [44] allied to current demands for international labor in institutions outside Brazil.

### Career trajectory patterns

In this section we discuss academic trajectory patterns, considering every stage in the education, as well as postdoctoral and work experiences of the INCT researchers. In this analysis, we only include degrees that have already been granted. For example, while all researchers in our dataset have at least one PhD entry, not all of them have finished their PhD studies yet. Thus, we only account for PhD stages that have been finished.

In the cases in which a researcher holds more than one degree of the same type (such as double PhDs), we consider only the first one. We did the same for researchers with multiple postdoctoral experiences. These cases are in general uncommon, but we observe an average of 1.48 postdocs per researcher and 1.12 undergraduate degrees per researcher (Table 9). Almost every researcher has at least one undergraduate degree and one PhD degree with valid entries in their CVs (over 97% of all researchers in each case). There is also a large proportion of Master’s degrees, and about half of the researchers have completed a postdoctoral stage.

**Table 9:** Number of graduated researchers per stage type.

	# stage holders	% stage holders	# total stages	stage/researcher
**Undergraduate**	5,811	97.29%	6,537	1.12
**Masters**	5,172	86.59%	5,405	1.05
**PhD**	5,866	98.21%	5,945	1.01
**Postdoctoral**	2,994	50.13%	4,439	1.48

For each researcher, we defined a trajectory consisting of a temporal sequence of academic and work stages, each of which related to a geographic region as defined in Section 4.4. We then performed a frequent pattern analysis on two sets of sequences: one consisting of the sequences of education degrees (from undergraduate to PhD) ending with a possible postdoc, and the other consisting of the sequences of geographic regions related to each stage in the trajectory. In the first case, we are looking for frequent behaviors as to the sequence of stages in the academic formation. In the second case, we are interested in analyzing the mobility of the researchers while pursuing their academic formation. The frequent pattern analysis algorithm simply counts the number of occurrences of a pattern, and selects those that account for at least θ% of the cases. In other words, these patterns have a *minimum support* of θ.

Table 10 shows the most common patterns on the education sequence, i.e., the sequence of degrees recorded in the CV. The table shows only combinations that account for θ = 1% or more of the patterns, corresponding to 84.9% of all INCT researchers. The two most common patterns are *Undergraduate → Masters → Doctorate* and *Undergraduate → Masters → Doctorate → Postdoctorate*. The direct move from an undergraduate degree to a PhD is much less common, indicating that the education of Brazilian scientists usually includes a Master’s stage, which is the usual standard in Brazilian institutions.

**Table 10:** Most common graduation trajectory patterns.

Pattern	# Researchers	Support %
Undergrad → Master → Doctorate	2,090	34.99%
Undergrad → Master → Doctorate → Postdoctorate	1,454	24.34%
Undergrad → Master→ Doctorate → Postdoctorate → Postdoctorate	437	7.32%
Undergrad → Doctorate → Postdoctorate	257	4.30%
Undergrad → Doctorate	248	4.15%
Undergrad → Bachelor → Master → Doctorate	187	3.13%
Undergrad → Master → Doctorate → Postdoctorate → Postdoctorate → Postdoctorate	133	2.23%
Undergrad → Doctorate → Postdoctorate → Postdoctorate	101	1.69%
Undergrad → Master	84	1.41%
Undergrad → Bachelor → Master → Doctorate → Postdoctorate	83	1.39%

Table 11 shows the most common patterns regarding the sequence of regions in which researchers performed each stage of their academic formation. Patterns were defined as a sequence *Undergraduate → Masters → Doctorate → Postdoctorate*, following the most common patterns found in the previous stage, with placeholders for the cases in which a stage has been skipped by the researcher. In the case of multiple entries for the same stage, we considered only the first one completed.

**Table 11:** Most common region trajectory patterns.

Pattern				# Researchers	Support %
Undergraduate	Master	Doctorate	Postdoctorate		
BSP	BSP	BSP	---	403	6.75%
BSE	BSE	BSE	---	370	6.19%
BSP	BSP	BSP	BSP	215	3.60%
BSU	BSU	BSU	---	178	2.98%
BNE	BNE	BNE	---	147	2.46%
BSP	BSP	BSP	NA	132	2.21%
BSE	BSE	BSE	BSE	126	2.11%
BSP	BSP	BSP	EU	122	2.04%
BSE	BSE	BSE	NA	95	1.59%
BSE	BSE	BSE	EU	76	1.27%
BSP	---	BSP	---	74	1.24%

BNE: Brazil Northeast

BSE: Brazil Southeast (except São Paulo)

BSP: São Paulo

BSU: Brazil South

EU: Europe

NA: North America.

The patterns shown in Table 11, which have a minimum support of 1%, account for 32.4% of the researchers in the dataset. The most common academic trajectory consists of completing every stage within São Paulo state. Indeed, the top 5 most frequent patterns in Table 11 show trajectories in which all stages were completed in the same region, indicating a low mobility of Brazilian researchers during their education. For the heterogeneous patterns, i.e., patterns that include at least one stage out of the original region, the most common situations show São Paulo and Southeast researchers seeking postdocs abroad. Finally, the most frequent patterns that present intra-country movements consist of researchers who move out of their original regions to seek a PhD in São Paulo. Europe and North America are the only foreign regions that appear in the most frequent trajectory patterns shown in Table 11.

When the stage corresponding to the employment institution is added to the analysis (Table 12), the tendency detected in the trajectory is reinforced. Notice that nearly the same patterns shown in Table 11 appear. For researchers from São Paulo and the Southeast, patterns that include a postdoc abroad (USA and Europe) also come up over the minimum support. In all cases with a minimum support of 1%, patterns show employment in the same region in which the undergraduate degree was obtained. The most frequent pattern that shows employment in a different region corresponds to undergraduate, master and PhD degrees in the Southeast and employment in the Midwest, which occurs only in 0.50% of the cases.

**Table 12:** Most common region trajectory patterns (considering employment).

Pattern					# Researchers	Support %
Undergraduate	Master	Doctorate	Postdoctorate	Employment		
BSE	BSE	BSE	---	BSE	285	4.77%
BSP	BSP	BSP	---	BSP	276	4.62%
BSP	BSP	BSP	BSP	BSP	154	2.58%
BSU	BSU	BSU	---	BSU	148	2.48%
BNE	BNE	BNE	---	BNE	126	2.11%
BSE	BSE	BSE	BSE	BSE	108	1.81%
BSP	BSP	BSP	NA	BSP	107	1.79%
BSP	BSP	BSP	EU	BSP	98	1.64%
BSE	BSE	BSE	NA	BSE	77	1.29%
BSE	BSE	BSE	EU	BSE	69	1.16%

BNE: Brazil Northeast

BSE: Brazil Southeast (except São Paulo)

BSP: São Paulo

BSU: Brazil South

EU: Europe

NA: North America.

Table 13 shows a similar pattern analysis, but now considering a coarser region description to better distinguish between intra-country and international mobility. We use the labels “A” to refer to the Brazilian region where the researcher concluded his undergraduate studies, “B” to refer to any other Brazilian region the researcher moved to in later stages, and “Abroad” to indicate stages which were done outside Brazil. The table accounts for 65.47% of the researchers in our dataset. Once again, the results shown in Table 13 confirm the observation that Brazilian researchers have a tendency to stay in the same region for most part of their academic lives. In most cases, researchers who do not work in their original region usually have moved out only after finishing their latest academic stage or as soon as they started a master’s degree. Moreover, we find no clear influence of stages taken abroad on the researchers’ mobility. For example, according to Table 13, of the 1,249 researchers in the table who have a PhD and/or a postdoc in a foreign institution, 1,017 of them (81%) have returned to their original region rather than moving to another Brazilian region.

**Table 13:** Most common generic trajectory patterns (considering employment).

Pattern					# Researchers	Support %
Undergraduate	Master	Doctorate	Postdoctorate	Employment		
A	A	A	---	A	876	14.67%
A	A	A	ABROAD	A	458	7.67%
A	A	A	A	A	317	5.31%
A	B	B	---	B	269	4.50%
A	A	ABROAD	---	A	202	3.38%
A	A	A	---	B	202	3.38%
A	A	ABROAD	ABROAD	A	180	3.01%
A	A	B	---	A	165	2.76%
A	B	B	---	A	164	2.75%
A	B	B	ABROAD	B	150	2.51%
A	A	B	---	B	112	1.88%
A	A	---	---	A	109	1.82%
A	---	A	---	A	107	1.79%
A	---	A	ABROAD	A	107	1.79%
A	B	B	B	B	101	1.69%
A	A	A	NULL	A	83	1.39%
A	A	A	ABROAD	B	82	1.37%
A	A	A	---	---	79	1.32%
A	A	A	A	B	78	1.31%
A	B	B	ABROAD	A	70	1.17%

A: institution is located in Brazil and in the same region where researcher obtained his undergraduate degree

B: institution is located in Brazil and in a region different from the one where researcher obtained his undergraduate degree

Abroad: institution is located outside Brazil

Null: the geocoding process could not determine the institution’s location.

**Fig 11:**
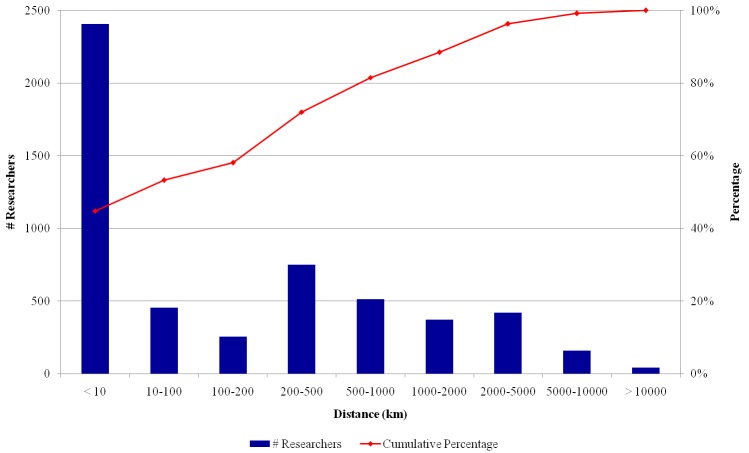
Distribution of the distance between the first and the last institutions in the career trajectories.

We also performed an analysis of the distribution of the geographic distances between the first and the last institutions in the trajectory, usually the undergraduate and the employment institutions. Fig 11 shows a large concentration of researchers who work less than 100 km away from the first institution. The share of researchers who established themselves in jobs farther away from the first point in their trajectories is low: less than 20% of the researchers work farther than 500 km away from the first institution.

The most frequent patterns confirm the tendency against moving to other parts of the country among Brazilian researchers. A large share of INCT researchers has conducted all stages of their education in the same region of the country. Foreign institutions only appear in the frequent patterns in the final trajectory point, e.g., for a postdoctoral stage. This analysis also confirms the tendency towards a reduced demand for PhD studies abroad, and establishes institutions located in São Paulo state as the focus of PhD demand from people educated elsewhere in the country. If we consider that it is desirable, in the education of a researcher, to seek a wider diversity of institutions, research groups or research centers, the frequent patterns found in this analysis suggest that some policies could be envisaged to promote more mobility. One possible explanation for the reduced mobility is the lack of appropriate funding or the lack of incentive to move, but such hypotheses cannot be tested against our current data. A national policy has been established in 2010 to incentivize undergraduate students to spend some time and do credit work in foreign institutions, a program called *Ciência Sem Fronteiras* (Science Without Borders). Whether this kind of program will have an influence over the next generations of researchers remains to be seen in the future, possibly using CiênciaBrasil data as a baseline. No similar program exists to incentivize the mobility within the country, except for senior scientists over short periods of time.

## Conclusions and Future Work

In this work we analyzed spatiotemporal aspects of a group of Brazilian researchers among the top research groups in the country. We characterized their career trajectories as recorded in their CVs, showing where they pursued their higher education, their fields of work and their geographic distribution, and analyzed how this behavior evolved over time. Even though this study is focused on a selected group of researchers, since our data comprises the CVs of the set of scientists involved in INCTs, their proportion as to the PhD holders in the country is expressive and reasonably comparable to the regional distribution.

These researchers are divided into eight knowledge areas, defined by the Brazilian government as key areas for INCTs. The number of researchers working in each group serves as indication to the political and scientific emphasis in these areas for the development of Brazilian research. Health Sciences is an expressive example, since it concentrates a large share of the researchers.

From the spatial analyses, we confirmed the tendency towards concentrating the education and the employment of INCT researchers in the Brazilian Southeast, notably São Paulo state. After all, the four southeastern states concentrate about 40% of the Brazilian population. Nevertheless, the Brazilian Northeast (26% of the population), North (8%), and Midwest (7%) are underrepresented in the distribution of INCT researchers, thus justifying policies that prioritize grants and research funding for groups based in those regions.

Looking at the trajectories, we observed a strong tendency towards seeking every career stage in institutions located in the same region. This contrasts sharply with European and USA studies that indicate a tendency towards higher mobility during and after advanced study stages [7, 8].

Within Brazil, São Paulo and Southeast institutions attract students from other parts of the country, but the distribution of qualified academic institutions seems to be improving, with a diminishing prevalence of degrees granted in São Paulo institutions over the recent years. International education is expressive, but concentrated at postdoc stages; about 80% of INCT researchers have obtained their PhDs in Brazil. International postdoc stages, after a full education in Brazil, indicate a drive towards seeking higher-level exchange and cooperation with foreign groups in a more advanced career stage.

This work opens new directions for investigating further issues, such as the mobility and regional representativeness of Brazilian science, and for assessing the impact of national policies for fostering research and development. Future work includes expanding the dataset to include all PhDs registered in the Lattes platform, as well as delve into further and deeper analyses, seeking a broader view of the career moves by Brazilian scientists.

## Supporting Information

S1 Table List of INCT researchers suplied by CNPq. (PDF)

## References

[1] Van Noorden R. The impact gap: South America by the numbers. Nature. 2014:2. 10.1038/510202a 24919906

[2] Almeida E, Chaves E, Guimarães JA. Brazil's growing production of scientific articles: how are we doing with review articles and other qualitative indicators? Scientometrics. 2013;97(2):287–315.

[3] Souza PNP. Structure and Operation of the Brazilian Higher Education System (in Portuguese): Editora Pioneira Ciências Sociais; 1991.

[4] Heitor. M, Horta H. Further Democratizing Latin America: Broadening Access to Higher Education and Promoting Science Policies Focused on the Advanced Training of Human Resources. Journal of Technology Management & Innovation. 2014;9(4):19.

[5] Laender AHF, Moro MM, Silva AS, Davis CA Jr, Gonçalves MA, Galante R, et al CiênciaBrasil—the Brazilian portal of science and technology Seminário Integrado de Software e Hardware (SEMISH); Natal (RN), Brazil: Sociedade Brasileira de Computação (SBC); 2011 p. 1366–79.

[6] Carvalho J, Maia R. Understanding EU and Brazilian Higher Education and Research: Policies, Frameworks and Structures. ALISIOS Short Paper 1. 2014:30.

[7] IDEA Consult. Study on mobility patterns and career paths of EU researchers Brussels, Belgium: European Commission, Research Directorate-General, 2010.

[8] Van Bouwel LAC. International mobility patterns of researchers and their determinants. Sumer Conference 2010 on Opening Up Innovation: Strategy, Organization and Technology; Imperial College London Business School2010. p. 1–26.

[9] Lee S, Bozeman B. The Impact of Research Collaboration on Scientific Productivity. Social Studies of Science. 2005;35(5):30.

[10] Bozeman B, Gaughan M. Impacts of grants and contracts on academic researchers’ interactions with industry. Research Policy. 2007;36(5):14.

[11] Horta H, Yonezawa A. Going places: exploring the impact of intra-sectoral mobility on research productivity and communication behaviors in Japanese academia. Asia Pacific Education Review. 2013;14(4):11.

[12] Song H. From Brain Drain to Reverse Brain Drain: Three Decades of Korean Experience. Science, Technology & Society. 1997;2(2):28.

[13] Arenas JLd, Castaños-Lomnitz H, Valles J, González E, Arenas-Licea J. Mexican scientific brain drain: causes and impact. Research Evaluation. 2001;10(2):5.

[14] Netz N, Jaksztat S. Mobilised by mobility? Determinants of international mobility plans among doctoral candidates in Germany In: Maadad N, Tight M, editors. International Perspectives on Higher Education Research. 11: Emerald Group Publishing Limited; 2014.

[15] Guth J, Gill B. Motivations in East-West Doctoral Mobility: Revisiting the Question of Brain Drain. Journal of Ethnic and Migration Studies. July 2008;34(5):17.

[16] The New Palgrave Dictionary of Economics. Palgrave Macmillan 2008. Brain drain.

[17] Balbachevsky E, do Couto e Silva E A diáspora científica brasileira: perspectivas para sua articulação em favor da ciência brasileira (in Portuguese). Revista Parcerias Estratégicas. 2011;16(33):14.

[18] Inanc O, Tuncer O. The effect of academic inbreeding on scientific effectiveness. Scientometrics. 2011;88(3):14.

[19] Heitor M, Horta H, Mendonça J. Developing human capital and research capacity: Science policies promoting brain gain. Technological Forecasting & Social Change. 2014;82:17.

[20] Padilla LE. How has Mexican faculty been trained? A national perspective and a case study. Higher Education: The International Journal of Higher Education and Educational Planning. 2007;56:17.

[21] Balancieri R, Bovo AB, Kern VM, Pacheco RCdS, Barcia RM. A análise de redes de colaboração científica sob as novas tecnologias de informação e comunicação: um estudo na Plataforma Lattes (in Portuguese). Ciência da Informação. 2005;34(1).

[22] Teixeira RKC, Gonçalves TB, Botelho NM. Analysis of the Brazilian Post-Graduates in Health Sciences: Current Situation per State (in Portuguese). UNOPAR Científica Ciências Biológicas e da Saúde. 2012;14(3).

[23] Cavalcante RA, Barbosa DR, Bonan PRF, Pires MBdO, Martelli-Júnior H. Profile of dentistry researchers of the Brazilian National Research Council (CNPq). Revista Brasileira de Epidemiologia. 2008;11(1):8.

[24] Morzinski JA, Schubot DB. Evaluating Faculty Development Outcomes by Using Curriculum Vitae Analysis. Family Medicine. 2000;32(3):5. 10726219

[25] Gaughan M, Bozeman B. Using curriculum vitae to compare some impacts of NSF research grants with research Center funding. Research Evaluation. 2002;11(1):17.

[26] Cañibano C, Bozeman B. Curriculum vitae method in science policy and research evaluation: the state-of-the-art. Research Evaluation. 2009;18(2):86.

[27] Furtado CA, Andrade TK, Davis Jr. CA. Geovisualization of The Academic Trajectories of Brazilian Researchers. XV Brazilian Symposium on Geoinformatics (GeoInfo 2015); Campos do Jordão (SP), Brazil2014. p. 83–94.

[28] Davis Jr CA, Fonseca FT, Borges KAV, editors. A flexible addressing system for approximate urban geocoding. V Brazilian Symposium on GeoInformatics (GeoInfo 2003); 2003; Campos do Jordão (SP).

[29] Davis CA Jr, Fonseca FT. Assessing the Certainty of Locations Produced by an Address Geocoding System. Geoinformatica. 2007;11(1):103–29. 10.1007/s10707-006-0015-7 PMC7087595 32214874

[30] Goldberg DW, Wilson JP, Knoblock CA. From text to geographic coordinates: the current state of geocoding. URISA Journal. 2007;19(1):33–46.

[31] Goldberg DW. Advances in geocoding research and practice. Transactions in GIS. 2011;15(6):727–33.

[32] Machado IMR, Alencar RO, Campos RO Jr, Davis CA Jr. An ontological gazetter and its application for place name disambiguation in text. Journal of the Brazilian Computer Society. 2011;17(4):267–79.

[33] Alencar RO, Davis CA Jr. Evaluation of the quality of an online geocoding resource in the context of a large Brazlian city. Transactions in GIS. 2011;15(6):851–68.

[34] Bornmann L, Waltman L. The detection of “hot regions” in the geography of science—A visualization approach by using density maps. Journal of Informetrics. 2011;5(4):7.

[35] Waltman L, Tijssen RJW, van Eck NJ. Globalisation of science in kilometres. Journal of Informetrics. 2011;5(4):9.

[36] Bornmann L, Leydesdorff L, Walch-Solimena C, Ettl C. Mapping excellence in the geography of science: An approach based on Scopus data. Journal of Informetrics. 2011;5(4):537.

[37] Leydesdorff L, Persson O. Mapping the Geography of Science: Distribution Patterns and Networks of Relations among Cities and Institutes. Journal of the American Society for Information Science & Technology. 2010;61(8):13.

[38] Cerny J, Nerad M. Postdoctoral Patterns, Career Advancement, and Problems. Science. 1999:3. 10.1126/science.285.5433.1533 10477510

[39] Martinelli D. A Brain Drain Among Young PhDs: Mirage or Reality? International Mobility of the Highly Skilled. 2002:9.

[40] Horta H. Holding a post-doctoral position before becoming a faculty member: does it bring benefits for the scholarly enterprise? Higher Education. 2009;58(5):33.

[41] Stephan P, Ma J. The Increased Frequency and Duration of the Postdoctorate Career Stage. The American Economic Review. 2005;95(2):5.

[42] Thompson J, Pearson M, Akerlind GS, Hooper J, Mazur N. Postdoctoral Training and Employment Outcomes. Canberra: Evaluations and Investigations Program, Higher Education Division DoE, Training and Youth Affairs, Commonwealth of Australia; 2001.

[43] Avellar SOdC. Internal migration of masters and doctors degree in Brazil: some considerations (in Portuguese). Revista Brasileira de Pós Graduação. 2014;11(24):20.

[44] Balbachevsky E. Academic Careers in Brazil: The Legacy of the Past. The Journal of the Professoriate. 2011;4(2):27.

[45] Balbachevsky E. Higher Education in Brazil: Different Worlds and Diverse Beliefs Comparative & International Higher Education. 2013;5:4.

[46] Pinto JMDR. O Acesso À Educação Superior No Brasil. Educação & Sociedade. 2004;25(88):30.

[47] Cantwell B. Academic in-sourcing: international postdoctoral employment and new modes of academic production. Journal of Higher Education Policy and Management. 2011;33(2):14.

[48] Cantwell B, Taylor BJ. Internationalization of the postdoctorate in the United States: analyzing the demand for international postdoc labor. Higher Education: The International Journal of Higher Education and Educational Planning. 2013;66(5):17.

