# Supplementary material for: A Spatiotemporal Analysis of Brazilian Science from the Perspective of Researchers’ Career Trajectories
Source: PLoS One. 2015 Oct 29;10(10):e0141528. doi: 10.1371/journal.pone.0141528 (PMC4626096; doi:10.1371/journal.pone.0141528)
Supplement: S1 Table — (PDF) [file pone.0141528.s001.pdf]

# Researcher	Researcher Name
1	Abelardo Antônio de Assunção Montenegro
2	Abelardo Silva Júnior
3	Abelmon da Silva Gesteira
4	Abilio Manuel Variz
5	Abilio Pereira de Lucena Filho
6	Abraham Chian-Long Chian
7	Abramo Hefez
8	Acácio Luiz Siarkowski
9	Adaildo Gomes D Assuncao
10	Adailton João Bortoluzzi
11	Adailton Silva Borges
12	Adalberto Fazzio
13	Adalberto José dos Santos
14	Adalberto Luis Val
15	Adalberto Pessoa Junior
16	Adalberto Ramon Vieyra
17	Adalbery Rodrigues Castro
18	Adalene Moreira Silva
19	Adalgiza Fornaro
20	Adalto Bianchini
21	Ada Maria de Barcelos Alves
22	Adamo Ferreira Gomes do Monte
23	Ádamo Lima de Santana
24	Adauto Jose Goncalves de Araujo
25	Adauto Lúcio Cardoso
26	Adauto Trigueiro de Almeida Filho
27	Adelaide Maria de Souza Antunes
28	Adelardo Adelino Dantas de Medeiros
29	Adelia Emilia de Almeida
30	Adelina Martha dos Reis
31	Adelina Pinheiro Santos
32	Ademar Lopes
33	Ademar Muraro Júnior
34	Ademir Castro e Silva
35	Ademir de Jesus Martins Júnior
36	Ademir Farias Morel
37	Ademir Neves
38	Adenilso da Silva Simão
39	Adenir da Silva Filho
40	Aderson da Silva Araújo
41	Aderson Farias do Nascimento
42	Adiel Mittmann
43	Adilson Elias Xavier
44	Adilson Marques da Cunha
45	Adla Betsaida Martins Teixeira
46	Adley Antonini Neves de Lima
47	Adley Forti Rubira
48	Admir Créso de Lima Targino
49	Adnei Melges de Andrade
50	Ado Jorio de Vasconcelos
51	Adolfo Gomes Marto
52	Adolfo Horn Junior
53	Adriana Augusto de Rezende
54	Adriana Benetti Marques Valio
55	Adriana Bittencourt Campaner
56	Adriana Campos Moreira Britto
57	Adriana Chatack Carmelo
58	Adriana Cristina Motta
59	Adriana Curi Aiub Casagrande

60	Adriana de Mello Gugliotta
61	Adriana de Souza Martins
62	Adriana Diaferia
63	Adriana Ferreira Uchôa
64	Adriana Fontes
65	Adriana Galindo Dalto
66	Adriana Karla Cardoso Amorim Reis
67	Adriana Maria Coimbra Horbe
68	Adriana Mary Mestriner Felipe de Melo
69	Adriana Nunes Correia
70	Adriana Pedrosa Biscaia Tufaile
71	Adriana Raffin Pohlmann
72	Adriana Ribeiro Silva
73	Adriana Rios Lopes
74	Adriana Santarosa Vivacqua
75	Adriana Silva Hemerly
76	Adriane Maria Ferreira Milagres
77	Adriane Ribeiro Rosa
78	Adriano Afonso Spielmann
79	Adriano Almeida Gonçalves Siqueira
80	Adriano Alonso Veloso
81	Adriano Antunes de Souza Araujo
82	Adriano Cesar Machado Pereira
83	Adriano Defini Andricopulo
84	Adriano Hoth Cerqueira
85	Adriano Lisboa Monteiro
86	Adriano Lorena Inacio de Oliveira
87	Adriano Luis Schünemann
88	Adriano Mauro Cansian
89	Adriano Mesquita Alencar
90	Adriano Monteiro de Castro Pimenta
91	Adriano Oliveira dos Santos
92	Adriano Otávio Maldaner
93	Adriano Trindade de Barros
94	Adriano Yacubian Fernandes
95	Adson Ferreira da Rocha
96	Adunias dos Santos Teixeira
97	Afonso Celso Dias Bainy
98	Afonso Celso Vigorito
99	Afonso de Moraes Paiva
100	Afonso Duarte Leão de Souza
101	Afonso Figueiredo Filho
102	Afonso Luís Barth
103	Afonso Nogueira
104	Afonso Paiva Neto
105	Afonso Rodrigues de Almeida
106	Agata Lages Gava
107	Agda Eunice de Souza
108	Agenor Pina da Silva
109	Agma Juci Machado Traina
110	Agnaldo Lopes da Silva Filho
111	Agnaldo Silva Martins
112	Agostinho Dirceu Didonet
113	Aguinaldo Robinson de Souza
114	Ailton Santa Barbara
115	Ailton Teixeira do Vale
116	Airam Jônatas Preto
117	Airton Nabarrete
118	Airton Tetelbom Stein
119	Ajith Kumar Sankarankutty

120	Akira Tanaka
121	Alain André Quivy
122	Alair Augusto Sarmet Moreira Damas dos Santos
123	Alan Carvalho Andrade
124	Alan Cavalcanti da Cunha
125	Álan Maia Borges
126	Albanita de Jesus Rodrigues da Silva
127	Albano Antonio da Silva Leite
128	Alba Valeria Rezende
129	Alberdan Santiago de Aquino
130	Albérico Blohem de Carvalho Júnior
131	Alberthmeiry Teixeira de Figueiredo
132	Albertina Pimentel Lima
133	Albert Josef Rudolf Bruch
134	Alberto Augusto Eichman Jakob
135	Alberto Barbosa Raposo
136	Alberto Cardoso Arruda
137	Alberto Carlos de Campos Bernardi
138	Alberto de Sampaio Ferraz Jardim Sayão
139	Alberto Felix Antonio da Nobrega
140	Alberto Groisman
141	Alberto Henrique Frade Laender
142	Alberto José Cavalheiro
143	Alberto Melo Soares
144	Alberto Paiva
145	Alberto Tufaile
146	Alberto Waingort Setzer
147	Albina Rodrigues Torres
148	Alcides José Monteiro da Silva
149	Alcides Lins Neto
150	Alcina Magnólia Franca Barreto
151	Alcindo Aparecido dos Santos
152	Alcindo José de Sá
153	Alcineia Conceição Oliveira
154	Alcino Palermo de Aguiar
155	Alcione Brasileiro Oliveira Cunha
156	Alcione Miranda dos Santos
157	Alcir Luiz Dafre
158	Alda Lucia Gomes Monteiro
159	Aldário Chrestani Bordonalli
160	Aldebaro Barreto da Rocha Klautau Júnior
161	Aldina Maria Prado Barral
162	Aldo Alfonso Raul Valcarce Bravo
163	Aldo Ângelo Moreira Lima
164	Aldo Eloizo Job
165	Aldo Jose Gorgatti Zarbin
166	Aldo Litaiff
167	Aldo Rogelis Aquiles Rodrigues
168	Aldo von Wangenheim
169	Aldrin Vieira Pires
170	Alejandra Kandus
171	Alejandro Antonio Fonseca Duarte
172	Alejandro Correa Dominguez
173	Alejandro Pedro Ayala
174	Alessandra Alves de Souza
175	Alessandra Aparecida Guarneri
176	Alessandra Greatti
177	Alessandro de Sousa Villar
178	Alessandro Martins da Costa
179	Alexander Charles Lees

180	Alexander Sibajev
181	Alexander Turra
182	Alexander Wilhelm Armin Kellner
183	Alexandra Alves Nicolau
184	Alexandra Ioppi Zugno
185	Alexandra Maria Monteiro Grisolia
186	Alexandra Susana Latini
187	Alexandre Afranio Peixoto
188	Alexandre Amstalden Moraes Sampaio
189	Alexandre Antunes Ribeiro
190	Alexandre Araújo Costa
191	Alexandre Archanjo Ferraro
192	Alexandre Bella Cruz
193	Alexandre Bragio Bonaldo
194	Alexandre Camargo Coutinho
195	Alexandre Cláudio Botazzo Delbem
196	Alexandre Cunha Ribeiro
197	Alexandre de Almeida e Silva
198	Alexandre de Almeida Prado Pohl
199	Alexandre de Avila Leripio
200	Alexandre de Magalhaes Vieira Machado
201	Alexandre Dias Ribeiro
202	Alexandre dos Santos Pyrrho
203	Alexandre Florian da Costa
204	Alexandre Guimaraes de Almeida Barros
205	Alexandre Holanda Sampaio
206	Alexandre Jean René Serres
207	Alexandre José Bühler
208	Alexandre José da Silva Góes
209	Alexandre José Macedo
210	Alexandre Leopold Busse
211	Alexandre Louis de Almeida DAvignon
212	Alexandre Luis Padovan Aleixo
213	Alexandre Luiz Amarante Mesquita
214	Alexandre Martins Valença
215	Alexandre Moraes Pinheiro
216	Alexandre Morais do Amaral
217	Alexandre Perez Umpierre
218	Alexandre Ricardo Soares Romariz
219	Alexandre Salino
220	Alexandre Santos de Alencar
221	Alexandre Savaris
222	Alex Andre Schmidt
223	Alexandre Soares de Oliveira
224	Alexandre Soares Rosado
225	Alexandre Souto Martinez
226	Alexandre Stamford da Silva
227	Alexandre Sztajnberg
228	Alexandre Vasconcellos
229	Alexandre Viecelli
230	Alexandre Vieira Werneck
231	Alexandre Zirpoli Simões
232	Alex Cavaliéri Carciofi
233	Alexei Manso Correa Machado
234	Alexei Mikhailovich Essiptchouk
235	Alex Fabiano Murillo da Costa
236	Alex Fiorini de Carvalho
237	Alex Niche Teixeira
238	Alexsandro Branco
239	Alexsandro Ferreira Cardoso da Silva

240	Alex Sandro Gomes
241	Alex Vladimir Krusche
242	Alfredo Augusto Cunha Alves
243	Alfredo Mayall Simas
244	Alfredo Miguel Ozorio de Almeida
245	Alfredo Noel Iusem
246	Alfredo Ribeiro Neto
247	Alfredo Ricardo Marques de Oliveira
248	Alfredo Wagner Berno de Almeida
249	Algemir Lunardi Brunetto
250	Alice Kazuko Inoue Nagata
251	Alice Maria Costa Martins
252	Alice Shimada Bacic
253	Alicia Juliana Kowaltowski
254	Alicia Norma González de Castells
255	Aline Maria da Silva
256	Aline Roberta Aceituno da Costa
257	Aline Schwarz
258	Aline Souza de Paula
259	Alisson Flávio Barbieri
260	Allan Cunha Barros
261	Allan Kardec Duailibe Barros Filho
262	Allbens Atman Picardi Faria
263	Almeriane Maria Weffort-Santos
264	Almir Diniz de Carvalho Júnior
265	Almir Goncalves Wanderley
266	Almir Spinelli
267	Alneu de Andrade Lopes
268	Aloa Machado de Souza
269	Aloir Antonio Merlo
270	Aloisio Pessoa de Araujo
271	Aloizio Soares Ferreira
272	Altamiro Amadeu Susin
273	Altay Alves Lino de Souza
274	Altigran Soares da Silva
275	Aluisio Augusto Cotrim Segurado
276	Alvair Pinto de Almeida
277	Alvaro Antonio Alencar de Queiroz
278	Alvaro Augusto da Costa Leitão
279	Alvaro Cantini Nunes
280	Alvaro Eduardo Eiras
281	Álvaro Luiz Heidrich
282	Álvaro Nogueira de Souza
283	Alvaro Penteado Crósta
284	Alvaro Ramon Coelho Ovalle
285	Álvaro Rossan de Brandão Prieto da Silva
286	Alvaro Toubes Prata
287	Alviclér Magalhães
288	Alysson Roncally Silva Carvalho
289	Amâncio Cesar Santos Friaça
290	Amanda Frederico Mortati
291	Amanda Latercia Tranches Dias
292	Amando Siuiti Ito
293	Amarildo Salina Ruiz
294	Amauri Arias Wenceslau
295	Amauri Pereira de Oliveira
296	Amaury Augusto de Almeida
297	Amelia Iaeca Kanagawa
298	Amélia Maria Ribeiro de Jesus
299	Amelia Teresinha Henriques

300	Américo Garcia da Silva Sobrinho
301	Amilcar Machulek Junior
302	Amilcar Pacheco
303	Amilcar Porto Pimenta
304	Amilcar Tanuri
305	Amilton Sinatora
306	Amin Bassrei
307	Amir Ordacgi Caldeira
308	Ana Acacia Pinheiro Caruso Neves
309	Ana Beatriz Albino de Almeida
310	Ana Beatriz Furlanetto Pacheco
311	Ana Campa
312	Ana Carla Oliveira da Silva Pinhati
313	Ana Carolina Brandao Salgado
314	Ana Carolina Japur de Sá Rosa e Silva
315	Ana Carolina Maisonnave Arisi
316	Ana Carolina Segato Rizzatti
317	Ana Catarina Peregrino Torres Ramos
318	Ana Cecilia Amado Xavier de Oliveira
319	Ana Cecília Ribeiro Cruz
320	Ana Celia Castro
321	Ana Célia Oliveira dos Santos
322	Ana Claudia Bonatto
323	Ana Claudia da Silva Andrade
324	Ana Cláudia de Macêdo Vieira
325	Ana Claudia do Amaral Melo
326	Ana Cláudia Duarte Cardoso
327	Ana Cláudia Fernandes Medeiros Braga
328	Ana Claudia Patrocinio
329	Ana Cláudia Pavarina
330	Ana Claudia Pinto Cottorello
331	Ana Claudia Queiroz Ladeira
332	Ana Cláudia Ruggieri
333	Ana Cristina Benso da Silva
334	Ana Cristina Castro Fontenla Sieira
335	Ana Cristina de Almeida Fernandes
336	Ana Cristina Figueiredo de Melo Costa
337	Ana Cristina Lima Leite
338	Ana Cristina Mendes de Oliveira
339	Ana Cristina Miranda Brasileiro
340	Ana Cristina Simões e Silva
341	Ana Cristina Victorino Krepischi
342	Ana Dóris de Castro
343	Ana Elisa Xavier de Oliveira e Dias
344	Ana Figueiredo Maia
345	Ana Flávia Barros da Silva Lima
346	Ana Flávia Granja e Barros
347	Ana Flávia Nogueira
348	Ana Gabriela Hounie
349	Ana Gisele da Costa Neves Ferreira
350	Ana Helena de Almeida Bressiani
351	Ana Isabela Araújo Cunha
352	Ana Leda de Faria Brino
353	Ana Lucia Abujamra
354	Ana Lúcia Cândido
355	Ana Lúcia da Costa Prudente
356	Ana Lúcia Escobar
357	Ana Lucia Ferreira de Barros
358	Ana Lucia Gomes dos Santos
359	Ana Lucia Kalinin

360	Ana Lúcia Kern
361	Ana Lúcia Mendes Cruz Silvestre da Silva
362	Ana Lucia Moraes Giannini
363	Ana Lucia Nogueira de Paiva Britto
364	Ana Lucia Pereira Schild
365	Ana Lúcia Rodrigues
366	Ana Lúcia Sampaio Sgambatti de Andrade
367	Ana Lúcia Teles Rabello
368	Ana Luisa Kerti Mangabeira Albernaz
369	Ana Luisa Palhares de Miranda
370	Ana Luiza Coelho Netto
371	Ana Luiza Costa Cruz Borges
372	Ana Luiza du Bocage Neta
373	Ana Luiza Queiroz Vilasbôas
374	Ana Luiza Silva Maia
375	Ana Maria Ambrosio
376	Anamaria Aranha Camargo
377	Ana Maria Benko Iseppon
378	Ana Maria Bergold
379	Ana Maria Blanco Martinez
380	Ana Maria Caetano de Faria
381	Ana Maria da Costa Ferreira
382	Ana Maria Dantas Barros
383	Ana Maria de Oliveira
384	Ana Maria de Paula
385	Ana Maria Dreher
386	Ana Maria Hermeto Camilo de Oliveira
387	Anamaria Martins Moreira
388	Ana Maria Massad Costa
389	Ana Maria Matildes dos Santos
390	Ana Maria Moro
391	Ana Maria Nicolaci da Costa
392	Ana Maria Rossini Teixeira
393	Ana Maria Sampaio Assreuy
394	Ana Maria Sell
395	Ana Marisa Chudzinski Tavassi
396	Ana Marisa Fusco Almeida
397	Ana Marli Christovam Sartori
398	Ana Maura Tomesani Marques
399	Anamélia Lorenzetti Bocca
400	Ana Neilde Rodrigues da Silva
401	Ananias Monteiro Mariz
402	Ana Odete Santos Vieira
403	Ana Patricia Yatsuda Natsui
404	Ana Paula Canedo Valente
405	Ana Paula de Azevedo Marques
406	Ana Paula do Nascimento Prata
407	Ana Paula Dutra de Aguiar
408	Ana Paula Fonseca
409	Ana Paula Fontana
410	Ana Paula Junqueira Kipnis
411	Ana Paula Lepique
412	Ana Paula Maielo Silva
413	Ana Paula Marins Chiaradia
414	Ana Paula Mendes de Miranda
415	Ana Paula Oliveira Brum
416	Ana Paula Salles Moura Fernandes
417	Ana Paula Silveira Paim
418	Ana Paula Soares Gondim
419	Ana Paula Terezan

420	Ana Paula Ulian de Araujo
421	Ana Paula Vidal Bastos
422	Ana Paula Vieira Colombo
423	Ana Rita de Araujo Nogueira
424	Ana Silvia Gouvêa Lima Yamada
425	Ana Tereza de Mendonça Viveiros Leal
426	Anatoli Leontiev
427	Ana Valéria Colnaghi Simionato
428	Ana Yoshi Harada
429	Anderson Caproni
430	Anderson de Jesus Gomes
431	Anderson Faustino da Silva
432	Anderson José Ferreira
433	Anderson Rodrigues Lima Caires
434	Anderson Stevens Leonidas Gomes
435	Anderson Zanardi de Freitas
436	Andrea Borghi Moreira Jacinto
437	Andrea Brito Latge
438	Andrea Cheble de Oliveira
439	Andrea Claudia Freitas Ferreira
440	Andréa Cristina Fogaça
441	Andréa Cristina Lima dos Santos
442	Andrea de Oliveira Ribeiro Junqueira
443	Andrea Fachel Leal
444	Andrea Ferraz Young
445	Andrea Ferreira da Costa
446	Andréa Gazzinelli Corrêa de Oliveira
447	Andrea Goncalves Trentin
448	Andréa Inês Horn Adams
449	Andrea Maciel de Oliveira Rossoni
450	Andrea Mara Macedo
451	Andrea Maria Aguilar
452	Andrea Maria Amaral Nascimento
453	Andrea Micke Moreno
454	Andréa Monteiro Santana Silva Brito
455	Andrea Queiroz Maranhao
456	Andrea Silveira de Souza
457	Andrea Siqueira Haibara
458	Andrea Thompson Da Poian
459	André Augusto Ferreira
460	Andre Augusto Gomes Faraco
461	André Auto Moreira
462	André Avelino Pasa
463	Andrea Vidal Ferreira
464	André Castro Lyra
465	André de Souza Avelar
466	André do Amaral Nogueira
467	Andre Galembeck
468	André Gonzaga dos Santos
469	Andreia Carvalho Alzamora
470	Andreia Costa Santos
471	Andréia Cristina Peres Rodrigues da Costa
472	Andréia Machado Leopoldino
473	Andreia Pereira Matos
474	Andréia Schmidt
475	Andréia Silva Flores
476	Andrei Koerner
477	Andreimar Martins Soares
478	Andre Junqueira Caetano
479	André Lopes Carvalho

480	André Luís Alice Raabe
481	Andre Luis Batista Ribeiro
482	Andre Luis da Cruz
483	Andre Luís de Medeiros Santos
484	André Luís dos Santos Silva
485	André Luís Marques Marcato
486	André Luis Willerding
487	Andre Luiz Barbosa Bafica
488	Andre Luiz de Oliveira
489	André Luiz Hemerly Costa
490	André Luiz Jardini Munhoz
491	André Luiz Lourenção
492	Andre Luiz Martinez de Oliveira
493	Andre Marco de Oliveira Gomes
494	Andre Mauricio Conceicao de Souza
495	Andre Nachbin
496	André Olmos Simões
497	André Portela Fernandes de Souza
498	Andre Ricardo Massensini
499	André Ricardo Pereira da Rosa
500	André Rodrigues
501	Andre Santarosa Ferlauto
502	André Soares de Oliveira
503	Andrés Pablo López Barbero
504	Andre Torre Neto
505	André Vargas Abs da Cruz
506	Andris Figueiroa Bakuzis
507	Anelise Beneduzi da Silveira
508	Anete Pereira de Souza
509	Anete Trajman
510	Angela Antonia Sanches Tardivo Delben
511	Angela Cristina Andrade Costa
512	Angela Cristina Krabbe
513	Angela Cristina Malheiros Luzo
514	Angela de Luca Rebello Wagener
515	Angela de Mello Ferreira
516	Angela Hampshire de Carvalho Santos Lopes
517	Angela Kaysel Cruz
518	Ángel Alberto Hidalgo
519	Angela Lucia Bagnatori Sartori
520	Angela Lúcia de Araújo Ferreira
521	Angela Luzia Branco Pinto Duarte
522	Angela Machado de Campos
523	Angela Malheiros
524	Ângela Maria de Miranda Freitas
525	Angela Maria Gordilho Souza
526	Ângela Maria Quintão Lana
527	Angela Maria Vianna Morgante
528	Angela Mehta dos Reis
529	Angélica Baptista Silva
530	Angelica Garcia Couto
531	Angélica Giarolla
532	Angelica Maria Penteado Martins Dias
533	Angelina Xavier Acosta
534	Angelita Cristine de Melo
535	Angelo Amâncio Duarte
536	Angelo Antonio Agostinho
537	Angelo Batista Miralha da Cunha
538	Angelo da Cunha Pinto
539	Angelo Ernani Maia Ciarlini

540	Angelo Gilberto Manzatto
541	Angelo Passaro
542	Angelo Rafael Carpinelli
543	Anibal Eugenio Vercesi
544	Anil Kumar Singh
545	Anke Bergmann
546	Anna Carla Renata Krepel Goldberg
547	Anna Claudia Domingos da Silveira da Luz
548	Anna Laura Lopes da Silva Nunes
549	Anna Luiza Ilkiu Borges Benkendorff
550	Anna Lvovna Okorokova Façanha
551	Anne Drumond Villela
552	Anne Marie Delaunay Maculan
553	Anne-Marie Pessis
554	Annibal Hetem Junior
555	Anselmo Fortunato Ruiz Rodriguez
556	Anselmo Gomes de Oliveira
557	Anselmo Salles Paschoa
558	Antonella Lombardi Costa
559	Antonella Maria Imperatriz Tassinari
560	Antonia Cecilia Zacagnini Amaral
561	Antonia Queiroz Lima de Souza
562	Antonia Railda Roel
563	Antonia Tavares do Amaral
564	Antonino Di Lorenzo
565	Antonio Alberto Fernandes de Oliveira
566	Antonio Alberto Rocha Oliveira
567	Antonio Aldo Melo Filho
568	Antonio Alfredo Ferreira Loureiro
569	Antonio Almeida Silva
570	Antonio André Novotny
571	Antonio Augusto Franco Garcia
572	Antonio Augusto Goncalves
573	Antonio Augusto Lisboa de Souza
574	Antonio Azevedo da Costa
575	Antonio Barros de Castro
576	Antonio Batista Pereira
577	Antonio Bernardo de Carvalho
578	Antonio Caetano Vaz Caltabiano
579	Antonio Candido de Camargo Guimarães Junior
580	Antonio Cardozo dos Santos
581	Antonio Carlos Benassi
582	Antonio Carlos Campos de Carvalho
583	Antônio Carlos de Abreu Mól
584	Antonio Carlos de Barros Correa
585	Antonio Carlos de Freitas
586	Antonio Carlos de Jesus Paes
587	Antonio Carlos de Oliveira
588	Antonio Carlos de Oliveira Barroso
589	Antonio Carlos Doriguetto
590	Antônio-Carlos Guimarães de Almeida
591	Antonio Carlos Hernandes
592	Antonio Carlos Marques Alvim
593	Antônio Carlos Rafael Barbosa
594	Antonio Carlos Roque da Silva Filho
595	Antonio Carlos Sansevero Martins
596	Antonio Carlos Santana dos Santos
597	Antonio Carlos Schneider Beck Filho
598	Antonio Carlos Seabra
599	Antonio Carlos Siqueira de Lima

600	Antonio Carlos Sobieranski
601	Antonio Carlos Zambroni de Souza
602	Antonio Castelo Filho
603	Antonio Celso Dantas Antonino
604	Antonio Cesar de Oliveira
605	Antonio Chalfun Junior
606	Antônio Chaves de Assis Neto
607	Antônio Cícero de Sousa
608	Antonio Claret Soares Sabioni
609	Antonio Claudio Tedesco
610	Antônio da Silva Souza
611	Antonio Donato Nobre
612	Antonio Eduardo Furtini Neto
613	Antonio Eduardo Martinelli
614	Antonio Egidio Nardi
615	Antonio Esio Bresciani
616	Antônio Euzébio Goulart Santana
617	Antonio Expedito Gomes de Azevedo
618	Antonio Fernando Bertachini de Almeida Prado
619	Antonio Flávio Medeiros Dantas
620	Antonio Francisco do Prado
621	Antonio Galina Filho
622	Antonio Giannella Neto
623	Antônio Gilberto Bertechini
624	Antonio Gilberto Ferreira
625	Antônio Gilson Gomes Mesquita
626	Antonio Gomes de Souza Filho
627	Antonio Gouveia de Souza
628	Antonio Helvecio Totola
629	Antonio Henrique da Fontoura Klein
630	Antônio Hugo José Fróes Marques Campos
631	Antonio João Paes de Barros
632	Antonio João Scandolera
633	Antonio Jorge Gomes Abelém
634	Antonio Jose da Costa Filho
635	Antonio Jose do Nascimento Dias
636	Antonio José Farias Nóbrega
637	Antonio José Felix de Carvalho
638	Antonio José Gonçalves
639	Antonio José Lapa
640	Antonio José Palangana
641	Antonio Jose Ramirez Londono
642	Antonio Lopes Apolinario Junior
643	Antonio Lucio Teixeira Junior
644	Antonio Luis Pacheco Rotondaro
645	Antonio Luiz Barbosa Pinheiro
646	Antonio Luiz Braga
647	Antonio Luiz Pinho Ribeiro
648	Antonio Luz Furtado
649	Antonio Macilio Pereira de Lucena
650	Antonio Manuel Alves Morais
651	Antonio Marcio Buainain
652	Antônio Marcos Gonçalves de Lima
653	Antônio Mário Magalhães
654	Antonio Martins Figueiredo Neto
655	Antonio Miguel Vieira Monteiro
656	Antonio Montes Filho
657	Antônio Nazareno Guimarães Mendes
658	Antonio Nemer Kanaan Neto
659	Antonio Ocimar Manzi

660	Antônio Pereira Júnior
661	Antonio Petraglia
662	Antonio Pires de Camargo
663	Antonio Ribeiro de Oliveira Junior
664	Antonio Ricardo Evangelista
665	Antonio Roberto Mury
666	Antônio Rodolfo de Faria
667	Antonio Ruffino Netto
668	Antonio Salvio Mangrich
669	Antonio Sérgio Alfredo Guimarães
670	Antonio Sérgio Magalhães de Castro
671	Antonio Tavares da Costa Junior
672	Antonio Themoteo Varela
673	Antonio Vargas de Oliveira Figueira
674	Antonio Vidiella Barranco
675	Antonio Waldo Zuardi
676	Antonio Walter Ferreira
677	Antonio Zelaquett Khoury
678	Aoi Masuda
679	Aparecida Maria Fontes
680	Aparecida Sadae Tanaka
681	Aparecido Jesuino de Souza
682	Aparecido Ribeiro de Souza
683	Aquilino Senra Martinez
684	Araken Alves de Lima
685	Araken dos Santos Werneck Rodrigues
686	Arandi Ginane Bezerra Junior
687	Arcilan Trevenzoli Assireu
688	Ardala Elisa Breda Andrade
689	Ardiley Torres Avelar
690	Ariane Baffa Lourenco
691	Ariane Luna Peixoto
692	Ari Digiácomo Ocampo Moré
693	Ariete Righi
694	Arildo José Braz de Oliveira
695	Ari Miguel Teixeira Ott
696	Aristides Moysés
697	Aristóbolo Mendes da Silva
698	Arlene de Jesus Mendes Caldas
699	Arlindo Philippi Jr
700	Armando Antonio Maria Laganá
701	Armando da Silva Cunha Júnior
702	Armando Martins Leite da Silva
703	Armando Muniz Calouro
704	Armenio Aguiar dos Santos
705	Armindo Santos
706	Arnaldo César Pereira
707	Arnaldo da Costa Faro Junior
708	Arnaldo de Albuquerque Araújo
709	Arnaldo Gammal
710	Arnaldo Leite Pinto Garcia
711	Arnaldo Rodrigues dos Santos Jr
712	Arndt von Staa
713	Arnildo Pott
714	Arnobio Antonio da Silva Junior
715	Arno Krenzinger
716	Arnola Cecilia Rietzler
717	Arnoldo Rocha Façanha
718	Aroldo Fernando Camargos
719	Aron Simis

720	Artemis Marti Ceschin
721	Arthur Ayres Neto
722	Arthur Giraldi Guimarães
723	Arthur José da Silva Rocha
724	Artur Avila Cordeiro de Melo
725	Artur da Silva Gouveia Neto
726	Artur José Santos Mascarenhas
727	Artur Oscar Lopes
728	Artur Stamford da Silva
729	Artur Ziviani
730	Ary Correa Junior
731	Aryeverton Fortes de Oliveira
732	Asher Kiperstok
733	Augusto César de Queiroz
734	Augusto César Ribeiro Figueiredo
735	Augusto Cezar Alves Sampaio
736	Augusto Damineli Neto
737	Augusto Santiago Cerqueira
738	Augusto Shinya Abe
739	Aura Conci
740	Aurélio Vicente Graça de Souza
741	Auri Marcelo Rizzo Vincenzi
742	Auro Atsushi Tanaka
743	Avelino Francisco Zorzo
744	Aymbiré Francisco Almeida da Fonseca
745	Bacy Fleitlich-Bilyk
746	Bárbara Amélia Aparecida Santana-Lemos
747	Barbara-Christine Marie Nentwig Silva
748	Bárbara Gomes Lupetti Baptista
749	Barbara Vasconcellos da Silva
750	Bartira Rossi Bergmann
751	Bazilio Frasco Vianez
752	Beate Saegesser Santos
753	Beatriz dos Santos Ferreira
754	Beatriz Machado Gomes
755	Beatriz Madalena Januzzi Mendes
756	Belita Koiller
757	Belmira Lara da Silveira Andrade da Costa
758	Benedito Carlos Cordeiro
759	Benedito Cláudio da Silva
760	Benedito de Moraes Purquerio
761	Benedito Dias Baptista Filho
762	Benedito Gomes dos Santos Filho
763	Ben-Hur Viana Borges
764	Benicio de Barros Neto
765	Benício Noronha Frey
766	Benildo Sousa Cavada
767	Benito Soto Blanco
768	Benjamim Bley de Brito Neves
769	Beny Spira
770	Bernadete Miranda dos Santos
771	Bernard Josiah Barlow
772	Bernardo Baldisserotto
773	Bernardo Lanza Queiroz
774	Bernardo Rangel Tura
775	Bernardo Ruegger Almeida Neves
776	Bernardo Stutz Xavier
777	Bernardo Walmott Borges
778	Bernhard Buhn
779	Bernhard Welz

780	Bertha Koiffmann Becker
781	Berthier Ribeiro de Araujo Neto
782	Bethania de Araujo Almeida
783	Bianca Cruz Neves
784	Bianca Gutfilen
785	Bibiana Verlindo de Araujo
786	Blície Jennifer Balisa Rocha
787	Boaventura Freire dos Reis
788	Bogdan Doboszewski
789	Boniek Evangelista Leite
790	Bráulio Batista Soares
791	Brenno Amaro DaSilveira Neto
792	Breno Satler de Oliveira Diniz
793	Britaldo Silveira Soares Filho
794	Bruce Bartholow Duncan
795	Bruce Walker Nelson
796	Bruna de Paula Fonseca e Fonseca
797	Bruno Brito Lisboa
798	Bruno Campello de Souza
799	Bruno de Vasconcelos Cardoso
800	Bruno Feijo
801	Bruno Geloneze Neto
802	Bruno Leonardo Canto Martins
803	Bruno Lobão Soares
804	Bruno Lourenço Diaz
805	Bruno Rezende de Souza
806	Bruno Richard Schulze
807	Bruno Szpoganicz
808	Bruno Vaz Castilho de Souza
809	Cacilda Borges do Valle
810	Cacilda da Silva Souza
811	Caetano Juliani
812	Caetano Traina Junior
813	Caio Julio Martins Veloso
814	Caio Mário Castro de Castilho
815	Caio Parente Barbosa
816	Cairo Lúcio Nascimento Júnior
817	Camila Alves Areda
818	Camila Alves de Rezende
819	Camila Castro Figueiredo
820	Camila Cherem Ribas
821	Camila Domeniconi
822	Camila Gambini Pereira
823	Camila Silva de Magalhães
824	Camilo Daleles Rennó
825	Candido Augusto Veloso Moura
826	Candido Ferreira Xavier de Mendonça Neto
827	Caridad Noda Pérez
828	Carina Friedrich Dorneles
829	Carina Rodrigues Boeck
830	Carísi Anne Polanczyk
831	Carla Alessandra Scorza
832	Carla Beatriz Collares Buzato
833	Carla Beatriz Grespan Bottoli
834	Carla Cecília Rodrigues Almeida
835	Carla Costa Teixeira
836	Carla Cristina Lopes de Azevedo
837	Carla Denise Bonan
838	Carla Gianna Luppi
839	Carla Holandino Quaresma

840	Carla Ines Tasca
841	Carla Lopes de Mendonça
842	Carla Martins Kaneto
843	Carla Máximo Prado
844	Carla Osthoff Ferreira de Barros
845	Carla Pagliari
846	Carla Ribeiro Polycarpo
847	Carla Roberta de Oliveira Carvalho
848	Carla Rodrigues Ribas
849	Carla Rosenberg
850	Carlile Campos Lavor
851	Carlo Requião da Cunha
852	Carlos Abraão Moura Valpassos
853	Carlos Afonso Nobre
854	Carlos Alberto Achete
855	Carlos Alberto Alves de Carvalho
856	Carlos Alberto Brayner de Oliveira Lira
857	Carlos Alberto da Silva Ledo
858	Carlos Alberto de Jesus Martinhon
859	Carlos Alberto Eiras Garcia
860	Carlos Alberto Estombelo Montesco
861	Carlos Alberto Etchevarne
862	Carlos Alberto Fortulan
863	Carlos Alberto Gallo
864	Carlos Alberto Gonçalves Silva Jared
865	Carlos Alberto Gurgel Veras
866	Carlos Alberto Heuser
867	Carlos Alberto Labate
868	Carlos Alberto Longui
869	Carlos Alberto Manssour Fraga
870	Carlos Alberto Montanari
871	Carlos Alberto Mororo Silva
872	Carlos Alberto Ospina Ramirez
873	Carlos Alberto Paskocimas
874	Carlos Alberto Paula Leite
875	Carlos Alberto Pereira Tavares
876	Carlos Alberto Petta
877	Carlos Alberto Rodrigues Costa
878	Carlos Alberto Saraiva Gonçalves
879	Carlos Alberto Spaggiari Souza
880	Carlos Alejandro Figueroa
881	Carlos Alejandro Nome
882	Carlos Alexandre Barboza Plinio dos Santos
883	Carlos Alexandre Borges Garcia
884	Carlos Alexandre Carollo
885	Carlos Alexandre Netto
886	Carlos Alexandre Sanchez Ferreira
887	Carlos Alexandre Wuensche de Souza
888	Carlos Antônio de Morais
889	Carlos Antonio Lopes de Oliveira
890	Carlos Augusto Colombo
891	Carlos Augusto da Silva Peres
892	Carlos Augusto de Oliveira Cavalcanti
893	Carlos Augusto Duque
894	Carlos Augusto França Schettini
895	Carlos Augusto Gomes Soares
896	Carlos Augusto Morales Rodriguez
897	Carlos Augusto Ramos e Silva
898	Carlos Augusto Romero Filho
899	Carlos Augusto Rosa

900	Carlos Aurélio Pimenta de Faria
901	Carlos Barbosa Alves de Souza
902	Carlos Basílio Pinheiro
903	Carlos Cauduro Schirmer
904	Carlos César Bof Bufon
905	Carlos Chesman de Araujo Feitosa
906	Carlos Curti
907	Carlos da Silva Vilar
908	Carlos De Marqui Junior
909	Carlos de Moura Neto
910	Carlos de Oliveira Paiva Santos
911	Carlos Dias Maciel
912	Carlos do Carmo Pagani Júnior
913	Carlos Eduardo Ambrósio
914	Carlos Eduardo Barra Couri
915	Carlos Eduardo Camargo de Albuquerque
916	Carlos Eduardo Cugnasca
917	Carlos Eduardo de Mesquita Barros
918	Carlos Eduardo de Rezende
919	Carlos Eduardo de Souza
920	Carlos Eduardo Ferreira Lopes
921	Carlos Eduardo Fisch de Brito
922	Carlos Eduardo Fortis Kwietniewski
923	Carlos Eduardo Freitas Lemos
924	Carlos Eduardo Frickmann Young
925	Carlos Eduardo Negrao
926	Carlos Eduardo Pellegrino Cerri
927	Carlos Eduardo Pereira
928	Carlos Eduardo Rolfsen Salles
929	Carlos Eduardo Soares Silvado
930	Carlos Eduardo Veiga de Carvalho
931	Carlos Eduardo Viana
932	Carlos Edwar de Carvalho Freitas
933	Carlos Faria Santos Amaral
934	Carlos Farley Herbster Moura
935	Carlos Fernando Teodósio Soares
936	Carlos Francisco Sampaio Bonafe
937	Carlos Frederico de Oliveira Graeff
938	Carlos Galup Montoro
939	Carlos Guillermo Giménez de Castro
940	Carlos Gustavo Brandão Corrêa de Castro
941	Carlos Gustavo Nunes da Silva
942	Carlos Gustavo Tamm de Araujo Moreira
943	Carlos Henrique de Castro
944	Carlos Henrique Inacio Ramos
945	Carlos Henrique Monken
946	Carlos Henrique Salvino Gadelha Meneses
947	Carlos Henrique Siqueira de Carvalho
948	Carlos Henrique Veiga
949	Carlos Jacinto da Silva
950	Carlos Jorge de Abreu
951	Carlos Jorge Logullo de Oliveira
952	Carlos Jorge Simal Rodrigues
953	Carlos José de Araújo
954	Carlos José Leopoldo Constantino
955	Carlos José Pereira de Lucena
956	Carlos José Pimenta
957	Carlos Lenz Cesar
958	Carlos Leomar Zani
959	Carlos Leonidas da Silva Souza Sobrinho

960	Carlos Machado Dias Jr
961	Carlos Magno Chaves Oliveira
962	Carlos Malamut
963	Carlos Manuel Sánchez Tasayco
964	Carlos Marcello Dias Fernandes
965	Carlos Martínez Ruiz
966	Carlos Maurício de Castro Costa
967	Carlos Mauricio Rabello de Sant'Anna
968	Carlos Maximiliano Dutra
969	Carlos Medicis Morel
970	Carlos Moreira de Souza Junior
971	Carlos Rafael García Hernández
972	Carlos Raimundo Ferreira Grosso
973	Carlos Rangel Rodrigues
974	Carlos Renato de Carvalho
975	Carlos Renato Rambo
976	Carlos Roberto Azzoni
977	Carlos Roberto de Mello Rieder
978	Carlos Roberto de Souza Filho
979	Carlos Roberto Ferreira Brandão
980	Carlos Rodrigo de Mello Roesler
981	Carlos Roney Armanini Tagliani
982	Carlos Teobaldo Gutierrez Vidalon
983	Carlos Termignoni
984	Carlos Tomei
985	Carlos William de Araujo Paschoal
986	Carlota de Oliveira Rangel Yagui
987	Carlton Anthony Taft
988	Carmem Aparecida de Paula
989	Carmem Juracy Silveira Gottfried
990	Carmem Lúcia de Paiva e Silva Zanta
991	Carmen Diva Saldiva de André
992	Carmen Eugenia Rodríguez Ortíz
993	Carmen Lúcia Cardoso
994	Carmen Lucia Penteado Lancellotti
995	Carmen Regla Vargas
996	Carmen Silvia Motta Bandini
997	Carmen Silvia Rial
998	Carmen Veríssima Ferreira
999	Carmo Roberto Pelliciari de Lima
1000	Carol Hollingworth Collins
1001	Carolina Alvares da Cunha de Azeredo Braga
1002	Carolina Arruda de Oliveira Freire
1003	Carolina Bhering de Araujo
1004	Carolina Blaya Dreher
1005	Carolina Horta Andrade
1006	Carolina Madeira Lucci
1007	Carolina Sales Vieira
1008	Carolina Weigert Galvão
1009	Caroline Argenta Pescador
1010	Carolyn Elinore Barnes Proenca
1011	Cassiano Monteiro Neto
1012	Cássia Ribeiro Ponciano
1013	Cassio Goncalves do Rego
1014	Cassio Leandro Dal Ri Barbosa
1015	Cassio Machiaveli Oishi
1016	Cássio Maldonado Turra
1017	Cássio Thomé de Faria
1018	Cassio van den Berg
1019	Cassius Vinicius Stevani

1020	Cassyano Januário Correr
1021	Catarina Akiko Miyamoto
1022	Catarina de Fatima Pereira Teixeira
1023	Catarina De Nigris Del Cistia
1024	Catarina Raposo Dias Carneiro
1025	Catari Vilela Chaves
1026	Catia Nunes da Cunha
1027	Cátia Suse de Oliveira Ribeiro
1028	Catia Urbanetz
1029	Cecília Jacques Gonçalves de Almeida
1030	Cecilia Machado
1031	Cecília Mary Fischer Rubira
1032	Cecilia Nahomi Kawagoe Suda
1033	Cecília Nunes Moreira
1034	Celene Tonella
1035	Celia de Andrade Lessa Kerstenetzky
1036	Célia Leite Sant'Anna
1037	Célia Machado Ronconi
1038	Célia Maria Giacheti
1039	Celia Priszkulnik Koiffmann
1040	Celia Regina Araujo Soares
1041	Célia Regina da Silva Garcia
1042	Celina Amalia Ramalho Galvão Lima
1043	Celina Maria Turchi Martelli
1044	Celio Estevan Moron
1045	Celio Pasquini
1046	Celio Ubirajara Magalhaes Filho
1047	Celli Rodrigues Muniz
1048	Celly Mieko Shinohara Izumi
1049	Celmy Maria Bezerra de Menezes Barbosa
1050	Celso Augusto Guimarães Santos
1051	Celso Caruso Neves
1052	Celso da Cruz Carneiro Ribeiro
1053	Celso de Amorim Camara
1054	Celso Eduardo Benedetti
1055	Celso Ferreira Filho
1056	Celso Jorge Villas-Bôas
1057	Celso Luiz Moretti
1058	Celso Luiz Salgueiro Lage
1059	Celso Marcelo Franklin Lapa
1060	Celso Massaki Hirata
1061	Celso Molina
1062	Celso Pinto de Melo
1063	Celso Vataru Nakamura
1064	Celso von Randow
1065	Cely Martins Santos de Alencar
1066	Cerli Rocha Gattass
1067	Cesar Albenes Zeferino
1068	Cesar Augusto Camillo Teixeira
1069	Cesar Augusto Cavalheiro Marcondes
1070	César Augusto Missio Marcon
1071	Cesar Augusto Moraes de Abreu
1072	César Augusto Sales Barbosa
1073	César Augusto Souza de Andrade
1074	Cesar Augusto Tischer
1075	Cesar Barreira
1076	César de Castro Martins
1077	Cesar Jose Deschamps
1078	Cesar Leopoldo Camacho Manco
1079	Cesar Liberato Petzhold

1080	Cesar Martins
1081	Cesar Mello
1082	Cesar Miranda Mendes
1083	César Ricardo Teixeira Tarley
1084	César Serra Bonifácio Costa
1085	Cesar Tadeu Pozzer
1086	César Wilhelm Massen Prieb
1087	Cezar Otaviano Ribeiro Negrão
1088	Chang Chung Yu Dorea
1089	Cháriston André Dal Belo
1090	Charles José Bonatto
1091	Charles Roland Clement
1092	Chin An Lin
1093	Chou Sin Chan
1094	Christian Dennys Monteiro de Oliveira
1095	Christiane Anneliese Gresse von Wangenheim
1096	Christiane de Arruda Rodrigues
1097	Christiane Fernandes Horn
1098	Christiane Meyre da Silva Bittencourt
1099	Christiane Pienna Soares
1100	Christian Haag Kristensen
1101	Christian Johann Losso Hermes
1102	Christian Macagnan Probst
1103	Christianne Bandeira de Melo
1104	Christianne Gazzana Salbego
1105	Christiano de Oliveira Braga
1106	Christiano José Santiago de Matos
1107	Christiano Vieira Pires
1108	Christina Alves Peixoto
1109	Christina von Flach Garcia Chavez
1110	Christina Wyss Castelo Branco
1111	Christine Strüssmann
1112	Christopher Kushmerick
1113	Christoph Friedrich Deneke
1114	Christovam Barcellos
1115	Cibele Marli Cação Paiva Gouvêa
1116	Cibele Rodrigues Bonvicino
1117	Cibele Velloso Rodrigues
1118	Cicera Henrique da Silva
1119	Cicero Brasileiro de Mello Neto
1120	Cicero da Rocha Souto
1121	Cicero Roberto Teixeira Regis
1122	Cid Bartolomeu de Araújo
1123	Cid Carvalho de Souza
1124	Cintia Lugnani Gomes de Amorim
1125	Ciro Abbud Righi
1126	Ciro Alberto de Oliveira Ribeiro
1127	Clarice Izumi
1128	Clarice Madalena Bueno Rolim
1129	Clarissa Damaso
1130	Clarissa Menezes Maya Monteiro
1131	Clarissa Severino Gama
1132	Clarisse Sieckenius de Souza
1133	Clascidia Aparecida Furtado
1134	Claubia Pereira Bezerra Lima
1135	Claudemir Zucareli
1136	Claudete Catanhede do Nascimento
1137	Claudete Esteves Nogueira Pinto Klumb
1138	Claudete Fernandes Pereira
1139	Claudete Justina Valduga

1140	Cláudia Akemi Kodaira Góes
1141	Cláudia Alcaraz Zini
1142	Claudia Alessandra Eckley
1143	Claudia Alessandra Fortes Aiub
1144	Claudia Alves Couto
1145	Claudia Aparecida Rainho
1146	Cláudia Bueno dos Reis Martinez
1147	Cláudia Cândida Silva
1148	Claudia Cardoso Martins
1149	Claudia Carvalhinho Windmoeller
1150	Claudia Cristina Garcia Martin Didonet
1151	Claudia de Alencar Santos Lage
1152	Claudia Domingues Vargas
1153	Claudia do Ó Pessoa
1154	Cláudia Duarte da Cunha
1155	Claudia Esther Alicia Rocio Hassan
1156	Claudia Farias Benjamim
1157	Claudia Fegadolli
1158	Cláudia Franco Corrêa
1159	Claudia Ida Brodskyn
1160	Claudia Ines Chamas
1161	Claudia Keller
1162	Claudia Leonor López Garcés
1163	Claudia Lima Caldeira
1164	Claudia Lima Verde Leal
1165	Claudia Linhares Sales
1166	Claudia Longo
1167	Claudia Lucia Mendes de Oliveira
1168	Claudia Maria Bauzer Medeiros
1169	Cláudia Maria de Andrade Equi
1170	Claudia Maria de Oliveira Raposo
1171	Claudia Maria Lima Werner
1172	Cláudia Maria Oliveira Simões
1173	Claudia Marina Fló
1174	Claudia Moraes de Rezende
1175	Claudia Perrone-Moisés
1176	Cláudia Ramos Rhoden
1177	Cláudia Regina da Silva
1178	Cláudia Rocha Martins
1179	Claudia Salviano Teixeira
1180	Claudia Torres
1181	Claudia Trindade Oliveira
1182	Cláudia Vilega Rodrigues
1183	Cláudia Vitória de Moura Gallo
1184	Claudine Massi Mynssen
1185	Cláudio Antônio Perottoni
1186	Claudio Augusto Oller do Nascimento
1187	Claudio Augusto Uyeda
1188	Claudio Bastos Pereira
1189	Claudio Benedito Silva Furtado
1190	Claudio da Cruz Silveira
1191	Claudio de Morisson Valeriano
1192	Cláudio Eduardo de Oliveira Cavalcanti
1193	Cláudio Estêvão Farias Cruz
1194	Claudio Fernando Mahler
1195	Claudio Gabriel Rodrigues
1196	Claudio Gustavo Stefanoff
1197	Cláudio Henrique Soares Del Menezzi
1198	Cláudio Jerônimo da Silva
1199	Claudio José Cavalcante Blanco

1200	Claudio Jose de Araujo Mota
1201	Claudio Jose Reis de Carvalho
1202	Cláudio Kitano
1203	Claudio Landim
1204	Claudio Lenz Cesar
1205	Cláudio Leonardo Lucchesi
1206	Claudio Luiz de Oliveira
1207	Cláudio Márcio do Nascimento Abreu Pereira
1208	Claudio Marques de Sá Medeiros
1209	Claudio Martin Pereira de Pereira
1210	Cláudio Melo
1211	Claudio Nery Lamarão
1212	Claudionor Gomes Bezerra
1213	Cláudio Radtke
1214	Claudio Roberto Bezerra dos Santos
1215	Claudio Thomas Bornstein
1216	Claudio Viegas Junior
1217	Claudivan Feitosa de Lacerda
1218	Claudson Ferreira Bornstein
1219	Claure Nain Lunardi Gomes
1220	Cleber Dario Pinto Kruel
1221	Cleber Francisco Alves
1222	Cleber Ibraim Salimon
1223	Cleber Nascimento do Carmo
1224	Cleber Palma Silva
1225	Cleber Renato Mendonça
1226	Cleber Rosito Pinto Kruel
1227	Clelia Mara de Paula Marques
1228	Clemente Augusto Souza Tanajura
1229	Clenio Figueiredo Salviano
1230	Cléo Alcantara Costa Leite
1231	Cleoni dos Santos Carvalho
1232	Cleonilson Protásio de Souza
1233	Cleslei Fernando Zanelli
1234	Cleube Andrade Boari
1235	Cleuza Maria de Faria Rezende
1236	Clodomiro Alves Junior
1237	Clodoveu Augusto Davis Junior
1238	Clóvis Antônio Petry
1239	Clovis Caesar Gonzaga
1240	Clovis Lasta Fritzen
1241	Clovis Milton Duval Wannmacher
1242	Clovis Vaz Parente
1243	Colombo Celso Gaeta Tassinari
1244	Concepta Margaret McManus Pimentel
1245	Constantino Tsallis
1246	Consuelo Latorre Fortes Dias
1247	Corina da Costa Freitas
1248	Cosme Roberto Moreira da Silva
1249	Cristiana Ferrari
1250	Cristiana Maria Toscano
1251	Cristian Antonio Rojas
1252	Cristiane Brasil Lima Ulbrich
1253	Cristiane da Fonte Ramos
1254	Cristiane de Bona da Silva
1255	Cristiane de Jesus Barbosa
1256	Cristiane del Corsso
1257	Cristiane Regina Guerino Furini
1258	Cristiane Ritter
1259	Cristiane Silvestre de Paula

1260	Cristian Follmer
1261	Cristiani Bürger
1262	Cristiani Lopes Capistrano Gonçalves de Oliveira
1263	Cristiano Barros de Melo
1264	Cristiano Das Neves Almeida
1265	Cristiano de Mello Gallep
1266	Cristiano Fantini Leite
1267	Cristiano Giacomelli
1268	Cristiano Krug
1269	Cristiano Lacorte
1270	Cristiano Luis Pinto de Oliveira
1271	Cristiano Monteiro de Barros Cordeiro
1272	Cristiano Valentim da Silva Lazoski
1273	Cristiano Valim Bizarro
1274	Cristina Almeida Cunha Filgueiras
1275	Cristina Bonorino
1276	Cristina Carvalho Pacheco
1277	Cristina de Araújo Lima
1278	Cristina Duarte Murta
1279	Cristina Engel de Alvarez
1280	Cristina Eunice Okuyama Costa
1281	Cristina Kurachi
1282	Cristina Márcia Dias
1283	Cristina Maria Assis Lopes Tavares da Mata Hermida Quintella
1284	Cristina Moreira Bonafé
1285	Cristina Northfleet de Albuquerque
1286	Cristina Reinert
1287	Cristina Rossi Nakayama
1288	Cristina Sayuri Maki
1289	Cristina Setim Freitas
1290	Cristina Souza Freire Nordi
1291	Cristina Wayne Nogueira
1292	Cristine Campos de Xavier Pinto
1293	Cristine Martins Gomes de Gusmão
1294	Cristovam Wanderley Picanço Diniz
1295	Cynthia Maria Kyaw
1296	Cyntia Helena Ravena Pinheiro
1297	Cyntia Maria Telles Fadel-Picheth
1298	Cyro Ketzer Saul
1299	Dagmar Ruth Stach - Machado
1300	Daisy Janice Aguilar Netz
1301	Dalci Mauricio Miranda de Oliveira
1302	Dalton Dario Serey Guerrero
1303	Dalton de Faria Lopes
1304	Dalton de Morisson Valeriano
1305	Dalton de Oliveira Fontes
1306	Dalva Cristina Baptista do Lago
1307	Damaris Kirsch Pinheiro
1308	Damião Pergentino de Sousa
1309	Daniela Borges Pavani
1310	Daniela Cisneiros
1311	Daniel Adrian Stariolo
1312	Daniela Lazzaro
1313	Daniela Mara de Oliveira
1314	Daniela Maria do Amaral Ferraz Navarro
1315	Daniela Martí Barros
1316	Daniela Parada Pavoni
1317	Daniel Artur Pinheiro Palma
1318	Daniela Uziel Rozental
1319	Daniela Valadão Freitas Rosa

1320	Daniel Basílio Zandonadi
1321	Daniel Cardoso de Carvalho
1322	Daniel Cardoso de Souza
1323	Daniel Carvalho Pimenta
1324	Daniel de Miranda Silveira
1325	Daniele Barbosa de Almeida Medeiros
1326	Daniele dos Santos Martins
1327	Daniel Eduardo Weibel
1328	Daniel Felinto Pires Barbosa
1329	Daniel Hioki
1330	Daniel Jonathan
1331	Daniel Joseph Hogan
1332	Daniella Castanheira Bartholomeu
1333	Daniel Lázaro Gallindo Borges
1334	Danielle Aparecida Rosa de Magalhães
1335	Danielle Cristhina Melo Ferreira
1336	Danielle da Glória de Souza
1337	Danielle Guimarães Almeida Diniz
1338	Danielle Nogueira Ramos
1339	Danielle Pereira Cavalcanti
1340	Danielle Regina da Silva Guerra
1341	Daniel Müller
1342	Daniel Oliveira Cajueiro
1343	Daniel Prá
1344	Daniel Ratton Figueiredo
1345	Daniel Reinaldo Cornejo
1346	Daniel Rettori
1347	Daniel Santos Mansur
1348	Daniel Schroeter Simião
1349	Daniel Schwabe
1350	Daniel Sigulem
1351	Daniel Soares de Almeida
1352	Daniel Thiele
1353	Daniel Varela Magalhães
1354	Daniel Vidal Pérez
1355	Daniel Weingaertner
1356	Danilo Camargo Igliori
1357	Dan Marchesin
1358	Dante Ferreira Franceschini Filho
1359	Dante Luis Chinaglia
1360	Dany Sanchez Dominguez
1361	Darci Clementino Lopes
1362	Darci Odloak
1363	Dario Abel Palmieri
1364	Dário Santos Junior
1365	Dario Simões Zamboni
1366	David Boris Paul Déharbe
1367	David Braga Fernandes de Oliveira
1368	David Driemeier
1369	David Lima Azevedo
1370	David Lopes de Castro
1371	David Mendes
1372	David Saitovitch
1373	Davies William de Lima Monteiro
1374	Davis Carvalho de Oliveira
1375	Davis Fernandes Ferreira
1376	Dayani Galato
1377	Débora Alves Maciel
1378	Debora Amado Scerni
1379	Debora Christina Muchaluat Saade

1380	Débora Cristina Hipólide
1381	Debora de Hollanda Souza
1382	Débora Figueiredo Mendonça do Prado
1383	Debora Foguel
1384	Debora Gonçalves Xisto
1385	Deborah dos Santos Garruti
1386	Débora Marques de Miranda
1387	Débora Romeo Bertola
1388	Débora Rosana Ribeiro Penido Araujo
1389	Debora Terezia Balogh
1390	Décio Sperandio
1391	Deiby Santos Gouveia
1392	Deise Lucy Oliveira Montardo
1393	Deisy das Graças de Souza
1394	Delano Gobbi
1395	Delano Medeiros Beder
1396	Delton Ricardo Soares Meirelles
1397	Delvio Sandri
1398	Demartonne Ramos França
1399	Demétrio da Silva Mutzenberg
1400	Dênia Antunes Saúde-Guimarães
1401	Denilson Rabelo
1402	Denise Aparecida Andrade de Oliveira
1403	Denise Bueno
1404	Denise Celeste Godoy de Andrade Rodrigues
1405	Denise das Mercês Camarano
1406	Denise Guliato
1407	Denise Maria Zezell
1408	Denise Navia Magalhães Ferreira
1409	Denise Peixoto Guimaraes
1410	Denise Pires de Carvalho
1411	Denise Rivera Tenenbaum
1412	Denise Rocha Gonçalves
1413	Denise Valle
1414	Denise Vilarinho Tambourgi
1415	Denis Fernando Wolf
1416	Denis Otavio Vieira de Andrade
1417	Denivaldo Cicero Pavão Lopes
1418	Denizar Vianna Araujo
1419	Dennis Armando Bertolini
1420	Denusa Wiltgen
1421	Deonisio Cieslinski
1422	Derlene Attili de Angelis
1423	Diana Noronha Nunes
1424	Diana Paula Andrade Pilling Guapyassú de Oliveira
1425	Diego Antonio Falceta Gonçalves
1426	Diego Bonatto
1427	Diego da Silva Alves
1428	Diego Pinheiro Aguiar
1429	Dieter Carl Ernst Heino Muehe
1430	Dimas Agostinho da Silva
1431	Dimas Tadeu Covas
1432	Dinaldo Cavalcanti de Oliveira
1433	Diogo Ferreira da Costa Patrão
1434	Diogo Gonzaga Jayme
1435	Diogo Paschoalini Volanti
1436	Diogo Pinheiro Fernandes Pedrosa
1437	Diogo Rizzato Lara
1438	Diogo Seibert Lüdtke
1439	Dioneia Camilo Rodrigues de Oliveira

1440	Dionne Cavalcante Monteiro
1441	Dirce Fernandes de Melo
1442	Dirce Maria Carraro
1443	Dirceu Bartolomeu Greco
1444	Dirceu Luis Herdies
1445	Dirse Clara Kern
1446	Diva Anelie de Araujo Guimaraes
1447	Divaldo de Almeida Sampaio
1448	Divaldo Pereira de Lyra Junior
1449	Divanizia do Nascimento Souza
1450	Diva Sonaglio
1451	Djairo Guedes de Figueiredo
1452	Dmitriev Victor
1453	Domingos Alves Rade
1454	Domingos de Jesus Rodrigues
1455	Domingos Fernandes Urbano Neto
1456	Domingos Savio Queiroz
1457	Domingos Tabajara de Oliveira Martins
1458	Dominique Corinne Hermine Fischer
1459	Domitila Pascoaloto
1460	Donato Alexandre Gomes Aranda
1461	Dora Ann Lange Canhos
1462	Dorgival Olavo Guedes Neto
1463	Doriane Picanço Rodrigues
1464	Dorotéa de Fátima Lobato da Silva
1465	Doroty Mesquita Dourado
1466	Dory Hélio Aires de Lima Anselmo
1467	Dosil Pereira de Jesus
1468	Douglas D'Assunção
1469	Douglas do Nascimento Silva
1470	Douglas dos Santos Pina
1471	Douglas Francisco Marcolino Gherardi
1472	Douglas Galante
1473	Doumit Camilios Neto
1474	Draulio Barros de Araujo
1475	Duane Barros da Fonseca
1476	Duarte Lopes de Oliveira
1477	Dulce Helena Siqueira Silva
1478	Dulce Maria de Araújo Melo
1479	Dulce Maria Tourinho Baptista
1480	Dulciene Maria de Magalhães Queiroz
1481	Dulcinea Maria Barbosa Campos
1482	Dulcineia Saes Parra Abdalla
1483	Durcilene Alves da Silva
1484	Durval Dourado Neto
1485	Durvanei Augusto Maria
1486	Ebenezer de Oliveira Silva
1487	Edcleide Maria Araújo
1488	Edecio Cunha Neto
1489	Edelclaiton Daros
1490	Edélti Faria Albertoni
1491	Edelto dos Santos Antunes
1492	Edenilson Eduardo Calore
1493	Edenio Detmann
1494	Edenir Rodrigues Pereira Filho
1495	Éder Paulo Vendrasco
1496	Edgard de Faria Corrêa
1497	Edgar de Alencar Teixeira
1498	Edgard Morya
1499	Edgardo Omar Taroco Aliano

1500	Edgar Marcelino de Carvalho Filho
1501	Edgar Merchan-Hamann
1502	Edgar Nunes de Moraes
1503	Edilane Aparecida da Silva
1504	Edila Vilela de Resende Von Pinho
1505	Edilene Oliveira da Silva
1506	Edilson Ferneda
1507	Edilson Márcio Almeida da Silva
1508	Edilson Reis Rodrigues Kato
1509	Edimar José de Oliveira
1510	Edinei Koester
1511	Edinilsa Ramos de Souza
1512	Edison da Rosa
1513	Edison Roberto Parise
1514	Edivaldo Herculano Correa de Oliveira
1515	Edivaldo Ximenes Ferreira Filho
1516	Edleno Silva de Moura
1517	Edmar Avellar Soares
1518	Edmar Chartone de Souza
1519	Edmilson Dias de Freitas
1520	Edmo José Dias Campos
1521	Edmundo Albuquerque de Souza e Silva
1522	Edmundo da Silva Braga
1523	Edmundo Marinho do Monte
1524	Edmundo Roberto Mauro Madeira
1525	Edna Alves dos Anjos Valotta
1526	Edna Maria Ramos de Castro
1527	Edna Tomiko Myiake Kato
1528	Ednéia Casagranda Bueno
1529	Ednildo Andrade Torres
1530	Ednildo de Alcantara Machado
1531	Edson Amaro Junior
1532	Edson Cocchieri Botelho
1533	Edson da Costa Bortoni
1534	Edson dos Santos Moreira
1535	Edson Guimarães Lo Turco
1536	Edson Holanda Teixeira
1537	Edson Ifarraguirre Moreno
1538	Edson José Paulino da Rocha
1539	Edson Kassar
1540	Edson Luiz da Silva
1541	Edson Massayuki Huziwara
1542	Edson Moleta Colodel
1543	Edson Norberto Cáceres
1544	Edson Noriyuki Ito
1545	Edson Paulo da Silva
1546	Edson Paulo Domingues
1547	Edson Perito Amorim
1548	Edson Roberto Leite
1549	Edson Rodrigues Filho
1550	Edson Rondinelli
1551	Eduardo Adriano Cotta
1552	Eduardo Alberto de Souza Neto
1553	Eduardo Alberto Fancello
1554	Eduardo Amaral Haddad
1555	Eduardo Araújo de Oliveira
1556	Eduardo Arruda Teixeira Lanna
1557	Eduardo Augusto Bezerra
1558	Eduardo Barbosa Coelho
1559	Eduardo Brandt de Oliveira

1560	Eduardo Carasek da Rocha
1561	Eduardo Carlos Bianca Bittar
1562	Eduardo Ceretta Moreira
1563	Eduardo Cesar Leão Marques
1564	Eduardo Cesar Zarzana
1565	Eduardo da Motta e Albuquerque
1566	Eduardo de Campos Valadares
1567	Eduardo de Jesus Oliveira
1568	Eduardo de Matos Nogueira
1569	Eduardo de Sá Mendonça
1570	Eduardo de Sequeira Esteves
1571	Eduardo do Valle Simões
1572	Eduardo Enrique Castilla
1573	Eduardo Geraldo Alves Coelho
1574	Eduardo Gomes Dutra do Carmo
1575	Eduardo Guimarães Couto
1576	Eduardo Henrique Bevitori Kling de Moraes
1577	Eduardo Henrique da Silva Aranha
1578	Eduardo Inacio Duzzioni
1579	Eduardo Isidoro Carneiro Beltrão
1580	Eduardo Janot Pacheco
1581	Eduardo Jorge da Silva Fonseca
1582	Eduardo Jorge Feres Filho
1583	Eduardo José Marandola Junior
1584	Eduardo José Melo dos Santos
1585	Eduardo Jose Viola
1586	Eduardo Landulfo
1587	Eduardo Leme Alves da Motta
1588	Eduardo Luiz Damiani Bica
1589	Eduardo Magalhães Rego
1590	Eduardo Maldonado Turra
1591	Eduardo Marques
1592	Eduardo Monteiro Martins
1593	Eduardo Moraes Rego Reis
1594	Eduardo Morgado Belo
1595	Eduardo Nicolau dos Santos
1596	Eduardo Nóbrega Pereira Lima
1597	Eduardo Peres Novais de Sá
1598	Eduardo Radovanovic
1599	Eduardo Ribeiro de Azevedo
1600	Eduardo Ribeiro dos Santos
1601	Eduardo Ricci Júnior
1602	Eduardo Sanches Stuchi
1603	Eduardo Santana de Almeida
1604	Eduardo Sávio Passos Rodrigues Martins
1605	Eduardo Seperuelo Duarte
1606	Eduardo Serra Cypriano
1607	Eduardo Siegle
1608	Eduardo Simões de Almeida
1609	Eduardo Tavares Paes
1610	Eduardo Todt
1611	Eduardo Vieira Martins
1612	Eduardo Winter
1613	Edvaldo Aparecido Amaral da Silva
1614	Edvaldo da Nobrega Gaiao
1615	Edvaldo Sabadini
1616	Edvani Curti Muniz
1617	Edviges Marta Ioris
1618	Edwil Aparecida de Lucca Gattas
1619	Edy Sousa de Brito

1620	Efrem Jorge Gondim Ferreira
1621	Egberto Pereira
1622	Egberto Reis Barbosa
1623	Elaine Bortoleti de Araújo
1624	Elaine Cristina Gavioli
1625	Elaine Gomes Quintana
1626	Elaine Malosso
1627	Elaine Maria Frade Costa
1628	Elaine Raniero Fernandes
1629	Elaine Tomasi
1630	Elcio Abdalla
1631	Elder Moreira Hemerly
1632	Eldo Campos
1633	Elen Aquino Perpetuo
1634	Elenara Maria Teixeira Lemos Senna
1635	Elena Vitalievna Goussevskaia
1636	Elene Cristina Pereira Maia
1637	Elenice Seixas Hanna
1638	Eleni Gomes
1639	Eleonora Kurtenbach
1640	Elfriede Marianne Bacchi
1641	Eliana Aparecida de Rezende Duek
1642	Eliana Elisabeth Diehl
1643	Eliana Feldberg
1644	Eliana Gertrudes de Macedo Lemos
1645	Eliana Lima da Fonseca
1646	Eliana Maria Beluzzo Dessen
1647	Eliana Martins Lima
1648	Eliana Navarro dos Santos Muccillo
1649	Eliana Reiko Matushima
1650	Eliana Rodrigues
1651	Eliana Saul Furquim Werneck Abdelhay
1652	Eliane Aparecida Campesatto
1653	Eliane Candiani Arantes
1654	Eliane Correa Miotto
1655	Eliane Cristina Locali
1656	Eliane Gasparino
1657	Eliane Ribeiro
1658	Elias Tadeu Fialho
1659	Elias Teodoro da Silva Júnior
1660	Eli Diniz
1661	Eliezer Jesus de Lacerda Barreiro
1662	Elina Bastos Caramao
1663	Elisabete de Santis Braga
1664	Elisabete Maria de Gouveia Dal Pino
1665	Elisabete Maria Zanin
1666	Elisabete Pereira dos Santos
1667	Elisabeth Aparecida Audi
1668	Elisabeth Mateus Yoshimura
1669	Elisabeth Neumann
1670	Elisa Helena Leão Fernandes
1671	Elisa Maria da Conceicao Pereira Reis
1672	Elisa Maria de Sousa Russo
1673	Elisa Napolitano e Ferreira
1674	Elisardo Corral Vasquez
1675	Elisa Yumi Nakagawa
1676	Elisete Aparecida Batista
1677	Elisete da Conceição Quintaneiro Aubin
1678	Eliseu José Guedes Pereira
1679	Elisiana Pereira de Oliveira

1680	Elissandra Nascimento de Moura Lima
1681	Elita Fontenele Urano de Carvalho
1682	Elizabeth da Silveira Neves OU Elizabeth Neves de Melo
1683	Elizabeth Franklin Chilson
1684	Elizabeth Igne Ferreira
1685	Elizabeth Pacheco Batista Fontes
1686	Elizabeth Pereira Mendes
1687	Elizabeth Salbé Travassos da Rosa
1688	Elizabeth Stankiewicz Machado
1689	Elizeu Antonio Rossi
1690	Elke Bromberg
1691	Ellen de Nazaré Souza Gomes
1692	Ellen Francine Barbosa
1693	Elliot Watanabe Kitajima
1694	Elma Regina Silva de Andrade Wartha
1695	Elmo Salomão Alves
1696	Eloi de Souza Garcia
1697	Eloisa de Oliveira Simões Saliba
1698	Elon Lages Lima
1699	Elsa Masae Mamizuka
1700	Elson Longo da Silva
1701	Elson Paiva de Oliveira
1702	Elton Luiz Dantas
1703	Elton Pinto Colares
1704	Elvio Carlos Moreira
1705	Ely Antonio Tadeu Dirani
1706	Ely Vieira Cortez
1707	Elza Berger Salema Coelho
1708	Elza Conceição de Oliveira Sebastião
1709	Elza Fernandes de Araujo
1710	Emanuel Carrilho
1711	Emanuel Giarolla
1712	Emanuel Maltempi de Souza
1713	Emanuel Negrão Macêdo
1714	Emanuel Sávio Cavalcanti Sarinho
1715	Emerson Guedes Pontes
1716	Emerson Jose Veloso de Passos
1717	Emerson Luís Lemos Marinho
1718	Emerson Marcelo Girotto
1719	Emerson Mariano da Silva
1720	Emerson Rodrigues de Camargo
1721	Emerson Silva Lima
1722	Emerson Ticona Fioretto
1723	Emico Okuno
1724	Emidio Vasconcelos Leitão da Cunha
1725	Emilia Celma de Oliveira Lima
1726	Emilia Correia
1727	Emilia Villani
1728	Emilie Suzanne Coudel
1729	Emilio Luiz Streck
1730	Emilson Pereira Leite
1731	Emmanoel Vieira da Silva-Filho
1732	Emmanuel Dias-Neto
1733	Emmanuel Pacheco Rocha Lima
1734	Enaldo Silva Vergasta
1735	Enderson Petrônio de Brito Ferreira
1736	Enéas Gomes Filho
1737	Ene Glória da Silveira
1738	Enio Bueno Pereira
1739	Ênio Farias de França e Silva

1740	Enio Frota da Silveira
1741	Ennio Marques Palmeira
1742	Enrico Jardim Clemente Santos
1743	Enrique Mario Boccardo Pierulivo
1744	Eraldo Rodrigues de Lima
1745	Erb Ferreira Lins
1746	Ercules Epaminondas de Sousa Teotonio
1747	Erica Etelvina Viana de Jesus
1748	Erica Freire Antunes
1749	Eric Alberto de Mello Fagotto
1750	Eric Ericson Fabris
1751	Erich Talamoni Fonoff
1752	Erich Vinicius de Paula
1753	Érico Marlon de Moraes Flores
1754	Erika Maria Monteiro Santos
1755	Erika Rosa Maria Kedor
1756	Erik Muxagata
1757	Erikson Felipe Furtado
1758	Erivelton Geraldo Nepomuceno
1759	Erna Geessien Kroon
1760	Ernesto Chaves Pereira de Souza
1761	Ernesto Fagundes Galvão
1762	Ernesto Pinheiro Borges
1763	Ernesto Torres de Azevedo Marques Jr
1764	Ernesto Vieira Neto
1765	Erno Harzheim
1766	Eros Comunello
1767	Ervin Kaminski Lenzi
1768	Eryvaldo Sócrates Tabosa do Egito
1769	Esper Abrao Cavalheiro
1770	Esper Georges Kallás
1771	Estela Maria Souza Costa Neves
1772	Estela Maris Freitas Muri
1773	Ester Cerdeira Sabino
1774	Ester Massae Okamoto Dalla Costa
1775	Estevam Rafael Hruschka Júnior
1776	Estevão Vicente Cavalcante Monteiro de Paula
1777	Esther Jean Langdon
1778	Esther Margarida Alves Ferreira Bastos
1779	Etel Rocha Vieira
1780	Etel Rodrigues Pereira Gimba
1781	Etelvino José Henriques Bechara
1782	Euclydes Marega Junior
1783	Eudenilson Lins de Albuquerque
1784	Eudes da Silva Velozo
1785	Eudes Eterno Fileti
1786	Eugene Francis Vinod Rebello
1787	Eugênio Ramos Bezerra de Mello
1788	Eunezio Antonio de Souza
1789	Eunice André
1790	Eunice Maia de Andrade
1791	Êurica Adélia Nogueira Ribeiro
1792	Euripedes Constantino Miguel
1793	Eustógio Wanderley Correia Dantas
1794	Euzi Conceição Fernandes da Silva
1795	Evaldo Gonçalves Pelaes
1796	Evaldo Jose Corat
1797	Evaldo Mendonca Fleury Curado
1798	Evandrino Gomes Barros
1799	Evandro Conforti

1800	Evandro Luiz Klein
1801	Evandro Sobroza de Mello
1802	Evandson José dos Anjos Silva
1803	Evaristo de Castro Junior
1804	Evelize Folly das Chagas
1805	Evelyn Martina Schuler Zea
1806	Everaldo Arashiro
1807	Everaldo Barreiros de Souza
1808	Everaldo Paulo de Medeiros
1809	Everton Ferreira Lima
1810	Ewerton Wagner Santos Caetano
1811	Expedito Carlos Lopes
1812	Eymar Silva Sampaio Lopes
1813	Ezzat Selim Chalhoub
1814	Fabiana Borges Teixeira dos Santos
1815	Fabiana Farias de Lima Guimarães
1816	Fabiana Leão Lopes
1817	Fabiana Villela da Motta
1818	Fabiane Caxico de Abreu Galdino
1819	Fabiane Gomes de Moraes Rego
1820	Fabiane Hiratsuka Veiga de Souza
1821	Fabiano André Narciso Fernandes
1822	Fabiano Cesarino
1823	Fabiano Elias Xavier
1824	Fabiano Fruett
1825	Fabiano Passuelo Hessel
1826	Fabiano Perin Gasparin
1827	Fabiano Severo Rodembusch
1828	Fabiano Silva
1829	Fábio Alan Carqueija Amorim
1830	Fabio Andre Machado Porto
1831	Fabio Augusto
1832	Fabio Bessa Lima
1833	Fábio Branco Vaz de Oliveira
1834	Fabio Ceneviva Lacerda Almeida
1835	Fabio Cesar Gozzo
1836	Fábio de Oliveira
1837	Fábio de Oliveira Ferreira
1838	Fábio de Oliveira Fialho
1839	Fabio de Oliveira Pedrosa
1840	Fábio de Souza Mendonça
1841	Fábio Dondeo Origo
1842	Fabio Fernandes Morato Castro
1843	Fábio Henrique Tavares de Oliveira
1844	Fábio Hissa Vieira Hazin
1845	Fábio Klamt
1846	Fábio Kummrow
1847	Fábio Kurt Schneider
1848	Fabíola Attié de Castro
1849	Fabiola Mariana Aguiar Ribeiro
1850	Fabíola Traina
1851	Fabio Lopes Olivares
1852	Fábio Luiz Buranelo Toral
1853	Fábio Luiz Teixeira Gonçalves
1854	Fábio Merçon
1855	Fabio Moyses Lins Dantas
1856	Fabio Murilo DaMatta
1857	Fábio Protti
1858	Fábio Rodrigo Piovezani Rocha
1859	Fabio Roland

1860	Fabio Rubio Scarano
1861	Fabio Trindade Maranhão Costa
1862	Fabrício Benevenuto de Souza
1863	Fabricio Carneiro Linhares
1864	Fabrício de Novaes Kucinskis
1865	Fabricio Ferrari
1866	Fabricio Guimarães Baptista
1867	Fabricio Machado Silva
1868	Fabrício Ronil Sensato
1869	Fabrício Simeoni de Sousa
1870	Fan Hui Wen
1871	Farid Chemale Junior
1872	Faruk Jose Nome Aguilera
1873	Fátima Aparecida Böttcher Luiz
1874	Fatima Buchele
1875	Fátima de Campos Buzzi
1876	Fátima de Lourdes dos Santos Nunes Marques
1877	Fatima Maria Zanon Zotin
1878	Fatima Regina Mena Barreto Silva
1879	Felicio Garino Junior
1880	Felipe Antonio de Lima Toledo
1881	Felipe Berocan Veiga
1882	Felipe Bohn
1883	Felipe Dal Pizzol
1884	Felipe Fernandes Fanchini
1885	Felipe Maia Galvão França
1886	Felipe Perecin
1887	Felipe Rudge Barbosa
1888	Felipe Santiago Chambergo Alcalde
1889	Félix Alexandre Antunes Soares
1890	Fenelon Martinho Lima Pontes
1891	Fernanda Antunes
1892	Fernanda Araujo Baião Amorim
1893	Fernanda Araújo Honorato
1894	Fernanda Borges de Araujo Paula
1895	Fernanda Carvalho de Queiroz Mello
1896	Fernanda Chiarello Stedile
1897	Fernanda Duarte Lopes Lucas da Silva
1898	Fernanda Faria
1899	Fernanda Freire Tovar Moll
1900	Fernanda Guarino De Felice
1901	Fernanda Gusmão de Lima Kastensmidt
1902	Fernanda Maria Pereira Raupp
1903	Fernanda Nervo Raffin
1904	Fernanda Vasconcelos de Almeida
1905	Fernando Afonso Salla
1906	Fernando Alves de Azevedo
1907	Fernando Antonio Crocomo
1908	Fernando Antonio Figueiredo Cardoso da Silva
1909	Fernando Antônio Pinto Barúqui
1910	Fernando Aparecido Sigoli
1911	Fernando Araujo Monteiro
1912	Fernando Ariel Genta
1913	Fernando Artur Brasil Danziger
1914	Fernando Augusto Bozza
1915	Fernando Augusto Soares
1916	Fernando Barbosa Júnior
1917	Fernando Batista da Costa
1918	Fernando Benetti
1919	Fernando Campos Mendonca

1920	Fernando Carlos Pagnocca
1921	Fernando Carvalho da Silva
1922	Fernando César Weber Rosas
1923	Fernando Claudio Zawislak
1924	Fernando Codá dos Santos Cavalcanti Marques
1925	Fernando Costa e Silva Filho
1926	Fernando Cristovam da Silva Jardim
1927	Fernando Dantas Nobre
1928	Fernando de Holanda Barbosa Filho
1929	Fernando de Magalhães Papaterra Limongi
1930	Fernando de Queiroz Cunha
1931	Fernando de Sá Del Fiol
1932	Fernando de Souza Costa
1933	Fernando Ely
1934	Fernando Fabriz Sodré
1935	Fernando Fachini Filho
1936	Fernando Felipe Ferreyra Hernandez
1937	Fernando Ferreira Costa
1938	Fernando Galembeck
1939	Fernando Gama de Miranda Netto
1940	Fernando Garcia de Mello
1941	Fernando Gehm Moraes
1942	Fernando Guadalupe dos Santos Lins Brandão
1943	Fernando Hallwass
1944	Fernando Henrique Furlan Gouvêa
1945	Fernando Jacques Althoff
1946	Fernando Jorge da Paixao Filho
1947	Fernando José da Silva Moreira
1948	Fernando Josepetti Fonseca
1949	Fernando Jose Spanhol
1950	Fernando Kok
1951	Fernando Lázaro Freire Junior
1952	Fernando Lessa Tofoli
1953	Fernando Magno Quintão Pereira
1954	Fernando Manuel Carvalho da Silva Santos
1955	Fernando Marcos dos Reis
1956	Fernando Martins Carvalho
1957	Fernando Menezes Campello de Souza
1958	Fernando Pedroni
1959	Fernando Rangel de Sousa
1960	Fernando Regla Vargas
1961	Fernando Roberto de Andrade Lima
1962	Fernando Roberto de Luna Parisio Filho
1963	Fernando Salgueiro Perobelli
1964	Fernando Santos Osório
1965	Fernando Schnaid
1966	Fernando Sérgio Castilhos Karam
1967	Fernando Sérgio Escócio Drummond Viana de Faria
1968	Fernando Soares Lameiras
1969	Fernando Thomé Kreutz
1970	Fernando Virgilio Roig
1971	Fernando Zagury Vaz de Mello
1972	Fernão Castro Braga
1973	Filipe Almeida do Prado Mendonça
1974	Filipe de Carvalho Victoria
1975	Flamarion Borges Diniz
1976	Flaminio Levy Neto
1977	Flávia Almeida Santos
1978	Flavia Borges Mury
1979	Flavia Carvalho Alcantara Gomes

1980	Flavia de Campos Mello
1981	Flavia de Paula
1982	Flavia Imbroisi Valle Errera
1983	Flavia Maria Avelar Goncalves
1984	Flavia Maria Netto
1985	Flávia Maria Santoro
1986	Flávia Regina Capellotto Costa
1987	Flavia Regina Souza Lima
1988	Flavia Sant'Anna Rios
1989	Flávia Thomaz Verechia Pereira
1990	Flávio Ataliba Flexa Daltro Barreto
1991	Flavio Augusto de Souza Berchez
1992	Flávio Barbosa Justino
1993	Flavio Caldas da Cruz
1994	Flávio Costa Miguens
1995	Flavio D'Amico
1996	Flavio Danni Fuchs
1997	Flavio da Silva Emery
1998	Flávio de Lemos Carsalade
1999	Flávio Donizeti Marques
2000	Flávio Guimarães da Fonseca
2001	Flávio Henrique Miranda de Araújo Freire
2002	Flávio Henrique Reginatto
2003	Flavio Jesus Luizão
2004	Flavio Jose Vieira Hasselmann
2005	Flavio Leandro de Souza
2006	Flavio Maggessi Viola
2007	Flávio Meira Borém
2008	Flavio Orlando Plentz Filho
2009	Flavio Pereira Kapczinski
2010	Flavio Ricardo Liberali Magajewski
2011	Flavio Santos Damos
2012	Flávio Vieira Meirelles
2013	Flavo Elano Soares de Souza
2014	Florian Karl Wittmann
2015	Francilene Amaral da Silva
2016	Francine Santos de Paula
2017	Francinete Francis Lacerda
2018	Francisca Cléa Florenço de Sousa
2019	Francisca Dionízia de Almeida Matos
2020	Francisca Helena Muniz
2021	Francisca Maria Alves Pinheiro
2022	Francisca Soares de Araújo
2023	Francisco Anacleto Barros Fidelis de Moura
2024	Francisco Antonio Rodrigues Barbosa
2025	Francisco Bruno Souza Oliveira
2026	Francisco Carlos de Carvalho Marinho
2027	Francisco Carlos Faria Lobato
2028	Francisco Carlos Fernandes De Paula
2029	Francisco Carlos Rocha de Barros Junior
2030	Francisco Carlos Rocha Fernandes
2031	Francisco Cézar Costa Nogueira
2032	Francisco Chagas da Silva Filho
2033	Francisco das Chagas Marques
2034	Francisco das Chagas Mota
2035	Francisco de Assis Alves Mourão Filho
2036	Francisco de Assis Aquino Gondim
2037	Francisco de Assis Leone
2038	Francisco de Assis Marques
2039	Francisco de Assis Rocha Neves

2040	Francisco de Assis Tavares Ferreira da Silva
2041	Francisco Eduardo Gontijo Guimaraes
2042	Francisco Eliseu Aquino
2043	Francisco Erivan de Abreu Melo
2044	Francisco Ernesto Moreno Bernal
2045	Francisco Eugenio Mendonça da Silveira
2046	Francisco Fernando Lamego Simões Filho
2047	Francisco Gerson Araújo
2048	Francisco Hélio Rola
2049	Francisco Heron de Carvalho Junior
2050	Francisco Hilario Rego Bezerra
2051	Francisco Inacio Pinkusfeld Monteiro Bastos
2052	Francisco Jaime Bezerra Mendonca Junior
2053	Francisco Jose Alves Lemos
2054	Francisco Jose Barcellos Sampaio
2055	Francisco Jose de Paula Filho
2056	Francisco José Lima Aragão
2057	Francisco José Monaco
2058	Francisco José Penteado Aranha
2059	Francisco José Roma Paumgartten
2060	Francisco Marcos de Assis
2061	Francisco Moura Filho
2062	Francisco Murilo Zerbini Junior
2063	Francisco Oscar de Siqueira Franca
2064	Francisco Radler de Aquino Neto
2065	Francisco Rafael Martins Laurindo
2066	Francisco Ricardo da Cunha
2067	Francisco Rolfsen Belda
2068	Francisco Savio Mendes Sinfronio
2069	Francisco Sircilli Neto
2070	Francisco Tadeu Rantin
2071	Francisco Vilar Brasileiro
2072	Francis Wagner Silva Correia
2073	François Christophe Cuisinier
2074	Françoise Toledo Reis
2075	Francois Germain Noel
2076	Frank Herbert Quina
2077	Franklin da Costa Silva
2078	Franklin David Rumjanek
2079	Franklin Massami Matinaga
2080	Franklin Riet Correa Amaral
2081	Frank Nelson Crespilho
2082	Frank Sill Torres
2083	Frantomé Bezerra Pachêco
2084	Frederic Gerard Christian Valentin
2085	Frederic Jean Georges Frezard
2086	Frederico Borges de Brito
2087	Frederico Caetano Jandre de Assis Tavares
2088	Frederico Costa Beber Vieira
2089	Frederico Dias Nunes
2090	Frederico Duarte Garcia
2091	Frederico Guilherme de Carvalho Cunha
2092	Frederico Rosa Borges de Holanda
2093	Fuad Kassab Junior
2094	Fulvio Alexandre Scorza
2095	Fulvio Andres Callegari
2096	Gabriela Alves Macedo
2097	Gabriela de Oliveira Paiva e Silva
2098	Gabriela Riet Correa Rivero
2099	Gabriel Armando Pellegatti Franco

2100	Gabriel Coutinho Barbosa
2101	Gabriel Eduardo Schütz
2102	Gabriel Grimaldi Filho
2103	Gabriel Rodrigues Hickel
2104	Gabriel Rodríguez de Freitas
2105	Gabriel Vieira Soares
2106	Galo Antonio Carrillo Le Roux
2107	Gandhi Rádis Baptista
2108	Gannabathula Sree Vani
2109	Gastão Cesar Bierrenbach Lima Neto
2110	Gean Vitor Salmoria
2111	Geber Barbosa de Albuquerque Moura
2112	Geciane Silveira Porto
2113	Gefeson Mendes Pacheco
2114	Gélio Mendes Ferreira
2115	Genylton Odilon Rêgo da Rocha
2116	George Alexandre DosReis
2117	George Carlos do Nascimento
2118	George Marconi de Araújo Lima
2119	George Sand Leão Araújo de França
2120	George Santos Marinho
2121	Georges Gérard Flexor
2122	Georg Friedrich Irion
2123	Georgia Correa Atella
2124	Geovanni Dantas Cassali
2125	Geraldo Alves da Silva
2126	Geraldo Barroso Cavalcanti Júnior
2127	Geraldo Bonorino Xexéo
2128	Geraldo César de Oliveira
2129	Geraldo Galdino de Paula Junior
2130	Geraldo Gileno de Sa Oliveira
2131	Geraldo Magela da Costa
2132	Geraldo Nagib Zahran Filho
2133	Geraldo Roberto Carvalho Cernicchiaro
2134	Gerald Weber
2135	Gerardo Cristino Filho
2136	Gerd Bruno da Rocha
2137	Gerhard Wunderlich
2138	Gerluce Alves Pontes da Silva
2139	Gerly Anne de Castro Brito
2140	Gerson Antônio Pianetti
2141	Gerson Aparecido Yukio Tomanari
2142	Gerson Oliveira Penna
2143	Gerusa da Silva Salles Corrêa
2144	Gervásio Protásio dos Santos Cavalcante
2145	Gesse Eduardo Calvo Nogueira
2146	Ghislaine Miranda Bonduelle
2147	Giácomo Balbinotto Neto
2148	Gifone Aguiar Rocha
2149	Gilberto Abate
2150	Gilberto Barbosa Domont
2151	Gilberto Batista de Souza
2152	Gilberto Camara Neto
2153	Gilberto Carlos Sanzovo
2154	Gilberto Corso Pereira
2155	Gilberto Fernandes de Sá
2156	Gilberto Fernando Fisch
2157	Gilberto Jose de Moraes
2158	Gilberto Lucio Benedito de Aquino
2159	Gilberto Marrega Sandonato

2160	Gilberto Pechoto de Melo
2161	Gilberto Petraconi Filho
2162	Gilberto Sachetto Martins
2163	Gilberto Schwartsmann
2164	Gilberto Úbida Leite Braga
2165	Gilberto Weissmuller
2166	Gil de Aquino Farias
2167	Gilmara Gonzaga Pedrosa
2168	Gilmara Regina Lima Feio
2169	Gilmar Silva Beserra
2170	Gilson Antonio Giraldi
2171	Gilson Inacio Wirth
2172	Gilson Rogério Zeni
2173	Gilton Mendes dos Santos
2174	Gilvan Sampaio de Oliveira
2175	Ginette Jalbert de Castro Faria
2176	Gino de Assis
2177	Giovana Krempel Fonseca Merighe
2178	Giovanna Machado
2179	Giovanni Dolif Neto
2180	Gisela Maria Dellamora Ortiz
2181	Gisele Daltrini Felice
2182	Gisele Fonseca Chagas
2183	Gisele Gus Manfro
2184	Gisele Lobo Pappa
2185	Gisele Olímpio da Rocha
2186	Gisele Picolo
2187	Gisele Ramos de Oliveira
2188	Gisele Silva Araújo
2189	Gisele Zapata-Sudo
2190	Giseli Klassen
2191	Gislaine Ribeiro Pereira
2192	Gislene Almeida Carvalho-Zilse
2193	Gislene de Fátima Pereira
2194	Giuliano Arns Rampinelli
2195	Giuliano Cesar Clososki
2196	Giuseppe Antonio Cirino
2197	Gladstone Alves da Silva
2198	Gladyston Rodrigues Carvalho
2199	Glauber Acunha Goncalves
2200	Glaucia de Oliveira Assis
2201	Glaucia Maria Moraes de Oliveira
2202	Glaucia Maria Pontes Mouzinho
2203	Glaucia Mendes Souza
2204	Glaucia Noeli Maroso Hajj
2205	Glaucia Regina Martinez
2206	Glaucio Lima Siqueira
2207	Glaucius Oliva
2208	Glauco Antonio Truzzi Arbix
2209	Glauco Augusto de Paula Caurin
2210	Glauco Fontgalland
2211	Glêdson Elias da Silveira
2212	Gleim Dias de Souza
2213	Glenda Mezarobba
2214	Gleyci Aparecida Oliveira Moser
2215	Gloria Emilia Petto de Souza
2216	Gloria Isolina Boente Pinto Duarte
2217	Gloria Regina Cardoso Braz
2218	Gonçalo Rendeiro
2219	Gorki Mariano

2220	Grace Gosmann
2221	Grace Schenatto Pereira Moraes
2222	Graciela Ines Bolzon de Muniz
2223	Grasiela Lopes Leães Pinho
2224	Graziela Heberlé
2225	Graziela Pereira Casali
2226	Graziella Anselmo Joanitti
2227	Gregory Thomas Kitten
2228	Greice Andreotti de Molfetta
2229	Griselda Esther Jara de Garrido
2230	Guadalupe Edilma Licona de Macedo
2231	Guilherme Augusto Limeira Araujo
2232	Guilherme Augusto Magalhães Junior
2233	Guilherme Becker Sander
2234	Guilherme Borges Fernandez
2235	Guilherme Corrêa de Oliveira
2236	Guilherme Franco Netto
2237	Guilherme Frederico Bernardo Lenz e Silva
2238	Guilherme Frederico Marranghello
2239	Guilherme Gonçalves Sotelo
2240	Guilherme Horta Travassos
2241	Guilherme Loureiro Werneck
2242	Guilherme Penello Temporão
2243	Guilherme Vanoni Polanczyk
2244	Gulnar Azevedo e Silva
2245	Gunter Ebeling
2246	Gunther Brucha
2247	Günther Fleck
2248	Gustavo Adolfo Saavedra Pinto
2249	Gustavo Alberto Perla Menzala
2250	Gustavo Amadeu Micke
2251	Gustavo Amaral Lanfranchi
2252	Gustavo Arantes Rosa Maciel
2253	Gustavo Barbosa Lima da Silva
2254	Gustavo Carlos Buscaglia
2255	Gustavo Conde Menezes
2256	Gustavo de Araujo Rojas
2257	Gustavo Fernandes Souza Andrade
2258	Gustavo Frederico Porto de Mello
2259	Gustavo Garcia Rigolin
2260	Gustavo Henrique Bianco de Souza
2261	Gustavo Henrique de Frias Castro
2262	Gustavo Henrique Goldman
2263	Gustavo Lazzaro Rezende
2264	Gustavo Manzon Nunes
2265	Gustavo Neuberger
2266	Gustavo Reis Wilke
2267	Gustavo Ribeiro Xavier
2268	Gustavo Soares Vieira
2269	Gustavo Sousa Pavani
2270	Haidi Dálida Lentz Fiedler Nome
2271	Hamer Nastasy Palhares Alves
2272	Hamilton Barbosa Napolitano
2273	Hamilton Cabral
2274	Hamilton Germano Pavao
2275	Handerson Jorge Dourado Leite
2276	Hans Raj Gheyi
2277	Hans Rogério Zimermann
2278	Haroldo Cesar Beserra de Paula
2279	Haroldo Fraga de Campos Velho

2280	Haroldo Naoyuki Nagashima
2281	Haroldo Ramanzini Júnior
2282	Haroldo Silveira Dorea
2283	Harold William Rosenberg
2284	Hatisaburo Masuda
2285	Hatsumi Mukai
2286	Haydée Glória Cruz Caruso
2287	Hector Nicolas Seuánez Abreu
2288	Hedison Kiuity Sato
2289	Heinsten Frederich Leal dos Santos
2290	Heitor Evangelista da Silva
2291	Heitor Franco de Andrade Junior
2292	Hektor Sthenos Alves Monteiro
2293	Helaine Carrer
2294	Helano de Sousa Castro
2295	Helder Barbieri Lacerda
2296	Helder Ferreira Teixeira
2297	Helder Lima de Queiroz
2298	Helder Louvandini
2299	Helder Nunes da Cunha
2300	Helder Rolim Florentino
2301	Helena Barreto dos Santos
2302	Helena Camarao Telles Ribeiro
2303	Helena Carla Castro
2304	Helena Coutinho Franco de Oliveira
2305	Helena Cristina da Silva de Assis
2306	Helena Judith Nussenzveig Lopes
2307	Helena Keiko Toma
2308	Helena Lobo Borges
2309	Helena Lutescia Luna Coelho
2310	Helena Maria Marcolla Araujo
2311	Helena Maria Petrilli
2312	Helena Passeri Lavrado
2313	Helena Paula Brentani
2314	Helena Serra Azul Monteiro
2315	Helenice Vital
2316	Helen Jamil Khoury
2317	Hélen Julie Laure
2318	Helen Maria Pontes Sotao
2319	Helga Cristina Almeida da Silva
2320	Helia Kawa
2321	Heliana Maria Ceballos Aguilar
2322	Hélida Monteiro de Andrade
2323	Helinando Pequeno de Oliveira
2324	Helio Alexandre Stefani
2325	Hélio Anderson Duarte
2326	Helio Chacham
2327	Hélio Crestana Guardia
2328	Hélio de Mattos Alves
2329	Helio dos Santos Migon
2330	Helio Jaques Rocha Pinto
2331	Helio José Corrêa Barbosa
2332	Hélio Pedrini
2333	Helio Raymundo Santos Silva
2334	Helio Waldman
2335	Helma Pinchemel Cotrim
2336	Helma Ventura Guedes
2337	Heloisa Almeida Cunha Filgueiras Pinède
2338	Heloisa de Arruda Camargo
2339	Heloisa de Oliveira Beraldo

2340	Heloisa Helena Motta Bandini
2341	Heloisa Maria Boechat-Roberty
2342	Heloisa Pacheco-Ferreira
2343	Heloisa Soares de Moura Costa
2344	Heloisa Sobreiro Selistre de Araujo
2345	Heloiza Helena Ribeiro Schor
2346	Helotonio Carvalho
2347	Heloysa Martins Carvalho Andrade
2348	Helton da Costa Santiago
2349	Helton José dos Reis
2350	Helvecio Della Coletta Filho
2351	Henriette Monteiro Cordeiro de Azeredo
2352	Henri Ivanov Boudinov
2353	Henri Michel Pierre Plana
2354	Henrique Bursztyn
2355	Henrique Cesar Pereira Figueiredo
2356	Henrique de Melo Jorge Barbosa
2357	Henrique Duarte da Fonseca Filho
2358	Henrique Eduardo Bezerra da Silva
2359	Henrique Krieger
2360	Henrique Marcelo Gualberto Pereira
2361	Henrique Olavo de Olival Costa
2362	Henrique Takachi Moriya
2363	Henrique Zeferino de Menezes
2364	Henry Pablo Lopes Campos e Reis
2365	Henry Socrates Lavalle Sullasi
2366	Herbert Toledo Martins
2367	Herch Moyses Nussenzveig
2368	Hérida Regina Nunes Salgado
2369	Herman Augusto Lepikson
2370	Hermano Frid Neto
2371	Hermes Alves Filho
2372	Hermi Felinto de Brito
2373	Hernan Armando Mamani
2374	Hernandes Faustino de Carvalho
2375	Hernan Francisco Terenzi
2376	Hernani Aquini Fernandes Chaves
2377	Heroldo Weber
2378	Hilário Alencar da Silva
2379	Hillegonda Maria Dutilh Novaes
2380	Hilma Lúcia Tavares Dias
2381	Hilton de Castro Chaves Júnior
2382	Hilton Tulio Costi
2383	Hipócrates de Menezes Chalkidis
2384	Hiram Larangeira de Almeida Junior
2385	Hiro Goto
2386	Homero Marinho Teixeira Leite Junior
2387	Homero Santiago Maciel
2388	Honoria de Fatima Gorgulho
2389	Horacio Alberto Dottori
2390	Horacio Santiago Rostagno
2391	Hosana Maria Debonsi
2392	Hossein Movasati
2393	Hougelle Simplício Gomes Pereira
2394	Hudson de Sousa Buck
2395	Hugo Alejandro Gallardo Olmedo
2396	Hugo Enrique Hernández Figueroa
2397	Hugo Fuks
2398	Hugo Luis Fragnito
2399	Hugo Vicente Capelato

2400	Hugo Vieira Neto
2401	Humber Furlan
2402	Humberto César Chaves Fernandes
2403	Humberto Gomes Ferraz
2404	Humberto Gracher Riella
2405	Humberto Josué de Oliveira Ramos
2406	Humberto Márcio Santos Milagre
2407	Humberto Marotta Ribeiro
2408	Humberto Prates da Fonseca Alves
2409	Humberto Reis Matos
2410	Humberto Remigio Gamba
2411	Humberto Ribeiro da Rocha
2412	Humberto Tonhati
2413	Hypolito Jose Kalinowski
2414	Iakov Veniaminovitch Kopelevitch
2415	Ian Castro-Gamboa
2416	Icaro Vitorello
2417	Ieda Lucia Viana Rosa
2418	Iêda Maria Garcia dos Santos
2419	Iêda Maria Novaes Ilha
2420	Ieda Maria Orioli
2421	Ieda Spacino Scarminio
2422	Ignacio José Godinho Delgado
2423	Ignacio Larrabide
2424	Igor Anatolievich Degterev
2425	Igor Polikarpov
2426	Igor Rodrigues de Assis
2427	Ilana Elazari Klein Coaracy Wainer
2428	Ilana Pinsky Streinger
2429	Ilana Zalcberg Renault
2430	Ilara Hämmerli Sozzi de Moraes
2431	Ilce Mara de Syllos Cólus
2432	Ildeberto Aparecido Rodello
2433	Ilka Boaventura Leite
2434	Ilmar Bernardo Graebner
2435	Ilya Lvovich Shapiro
2436	Ilza Araujo Leao de Andrade
2437	Ilza Maria Urbano Monteiro
2438	Ima Célia Guimarães Vieira
2439	Inácio de Loiola Meirelles Junqueira de Azevedo
2440	Iná Elias de Castro
2441	Inaia Maria Moreira de Carvalho
2442	Indianara Maria Araujo do Nascimento
2443	Ing Hwie Tan
2444	Ingrid Dragan Taricano
2445	Ingrid Oliveira de Nunes
2446	Ingrid Tavora Weber
2447	Ioav Waga
2448	Iolanda Margherita Fierro
2449	Iolanda Midea Cuccovia
2450	Ione Salgado
2451	Ioshiaki Doi
2452	Iracema Fonseca de Albuquerque Cavalcanti
2453	Iracema Maria Castro Coimbra Cordeiro
2454	Iracilda Zeppone Carlos
2455	Iran Borges
2456	Iranderly Fernandes de Fernandes
2457	Iran Pereira Veiga Junior
2458	Irapuan Rodrigues de Oliveira Filho
2459	Irene da Silva Soares

2460	Irene Fernandes
2461	Irina Kerkis
2462	Irismar Reis de Oliveira
2463	Irving Foster Brown
2464	Irwin Rose Alencar de Menezes
2465	Isaac Costa Lázaro
2466	Isabela Heineck
2467	Isabel Altenfelder Santos Bordin
2468	Isabel Cristina Sales Fontes Jardim
2469	Isabel Harb Manssour
2470	Isabella Bias Fortes
2471	Isabella D'Andrea Meira
2472	Isabella Nascimento
2473	Isabel Oliveira de Oliveira
2474	Isabel Tourinho Salamoni
2475	Isac Almeida de Medeiros
2476	Ise de Goreth Silva
2477	Isis Maria Quezado Soares Magalhães
2478	Isolde Terezinha Santos Previdelli
2479	Israel Felzenszwalb
2480	Israel Jacob Rabin Baumvol
2481	Israel Vainsencher
2482	Itabajara da Silva Vaz Junior
2483	Italo Marcos Nunes de Oliveira
2484	Italo Odone Mazali
2485	Itamar Soares de Melo
2486	Itana Maria de Souza Gimenes
2487	Itzhak Roditi
2488	Iva Carneiro Leao Barros
2489	Ivair Aparecido dos Santos
2490	Ivaldo da Silva
2491	Ivaldo Rodrigues da Trindade
2492	Ivana Beatrice Mânica Da Cruz
2493	Ivana Cristina de Holanda Cunha Barreto
2494	Ivana Lucia de Oliveira Nascimento
2495	Ivan Antônio Izquierdo
2496	Ivana Veraldo
2497	Ivana Zanella da Silva
2498	Ivan Chestakov
2499	Ivan Cruz
2500	Ivan da Rocha Pitta
2501	Ivan dos Santos Oliveira Júnior
2502	Ivan Goncalves de Souza
2503	Ivan Guillermo Solórzano-Naranjo
2504	Ivan Helmuth Bechtold
2505	Ivani Malvestiti
2506	Ivano Damião Soares
2507	Ivan Sebastião de Souza e Silva
2508	Ivan Torres Pisa
2509	Ivete de Araújo Roland
2510	Ivo Antonio Dussin
2511	Ivo Bussoloti Filho
2512	Ivo Chaves da Silva Junior
2513	Ivo Lebrun
2514	Ivo Milton Raimundo Junior
2515	Ivone Carvalho
2516	Ivonete Batista de Araújo
2517	Izabel Cristina Riegel-Vidotti
2518	Izabelle Auxiliadora Molina de Almeida Teixeira
2519	Izabel Vianna Villela

2520	Izaltina Silva Jardim Cavalli
2521	Izan de Castro Leao
2522	Izeni Pires Farias
2523	Izilda Marcia Ranieri
2524	Izildinha de Souza Miranda
2525	Jacks Jorge Junior
2526	Jackson Antônio Marcondes de Souza
2527	Jackson Braz Marcinichen
2528	Jackson de Souza Menezes
2529	Jackson Fernando Rego Matos
2530	Jacob Palis Junior
2531	Jacobus Willibrordus Swart
2532	Jacqueline Albino
2533	Jacqueline Arguello Da Silva
2534	Jacqueline de Souza
2535	Jacqueline Nelisis Zanoni
2536	Jacqueline Sinhoretto
2537	Jacques Marcovitch
2538	Jacques Raymond Daniel Lépine
2539	Jacques Robert Nicoli
2540	Jacques Wainer
2541	Jacques Waldmann
2542	Jacyra Ramos Soares
2543	Jader Morais Borges
2544	Jader Riso Barbosa Junior
2545	Jael Soares Batista
2546	Jaidete Monteiro de Souza
2547	Jailson Bittencourt de Andrade
2548	Jailson Souza de Alcaniz
2549	Jaime Bruck Ripoll
2550	Jaime Cesar Coelho
2551	Jaime de Liege Gama Neto
2552	Jaime Edilberto Munoz Rivera
2553	Jaime Eduardo Cecilio Hallak
2554	Jaime Fernando Villas da Rocha
2555	Jaime Wilson Vargas de Mello
2556	Jair Carlos Checon de Freitas
2557	Jair Cavalcanti Leite
2558	Jair de Jesus Mari
2559	Jairo Kenupp Bastos
2560	Jairo Panetta
2561	Jairo Pereira Neves
2562	Jair Putzke
2563	Jairton Dupont
2564	Jamal da Silva Chaar
2565	Jamary Oliveira Filho
2566	Jamile Dehaini
2567	Janaina Carla dos Santos
2568	Janaina da Silva Crespo
2569	Janaina Fernandes
2570	Janaina Goncalves Guimaraes
2571	Janaina Ribeiro Costa Rouws
2572	Janaína Versiani dos Anjos
2573	Janaina Viana de Melo
2574	Jan Bitoun
2575	Jander Moreira
2576	Jandir Miguel Hickmann
2577	Jandyr de Menezes Travassos
2578	Jane Cristina de Oliveira Faria
2579	Jane Cristina Gregorio-Hetem

2580	Jane de Jesus da Silveira Moreira
2581	Janete Eliza de Sá Soares
2582	Janete Eunice Zorzi
2583	Jan Frans Willem Slaets
2584	Jânia Perla Diógenes de Aquino
2585	Janilo Santos
2586	Janine Inez Rossato
2587	Janne Cavalcante Monteiro
2588	Jannifer Oliveira Chiang
2589	Jano Moreira de Souza
2590	Jansen Alfredo Sampaio Zuanon
2591	Jaquelline Germano de Oliveira
2592	Jaques Kerstenetzky
2593	Jarbas Caiado de Castro Neto
2594	Jarbas Honorio de Miranda
2595	Jarbas Jose Rodrigues Rohwedder
2596	Jauvane Cavalcante de Oliveira
2597	Javier Alcides Ellena
2598	Javier Tomasella
2599	Javier Walter Ghibaudi
2600	Jayme Luiz Szwarcfiter
2601	Jay Wallace da Silva e Mota
2602	Jean Carlos Cardozo da Silva
2603	Jeane Eliete Laguila Visentainer
2604	Jeanine Maria Felfili Fagg
2605	Jean Luiz Simões de Araújo
2606	Jean Michel Lafon
2607	Jean Nunes dos Santos
2608	Jeanny da Silva Maciel
2609	Jean Pierre Henry Balbaud Ometto
2610	Jean Pierre Oses
2611	Jean Pierre von der Weid
2612	Jean Remy Davee Guimaraes
2613	Jeferson Jacob Arenzon
2614	Jeferson Klein
2615	Jeferson Luis Franco
2616	Jeferson Vieira Ramos
2617	Jefferson Antonio Galves
2618	Jefferson Bettini
2619	Jefferson Cardia Simões
2620	Jefferson Luiz Gomes Correa
2621	Jefferson Soares da Costa
2622	Jenner Karlisson Pimenta dos Reis
2623	Jennifer Lowe
2624	Jeremy Paul Jean Loup Deturche
2625	Jeroniza Nunes Marchaukoski
2626	Jerson Lima da Silva
2627	Jerson Rogério Pinheiro Vaz
2628	Jerusa Simone Garcia
2629	Jesse Carvalho Costa
2630	Jesus Antonio Berrocal Gomez
2631	Jez Willian Batista Braga
2632	Joanis Tilemahos Zervoudakis
2633	João Alberto Passos Filho
2634	Joao Alfredo Pinto de Magalhaes
2635	Joao Andrade de Carvalho Junior
2636	João Angelo Martini
2637	Joao Antonio Belmino dos Santos
2638	Joao Antonio Lorenzzetti
2639	João Antonio Martino

2640	Joao Antonio Pegas Henriques
2641	João Antonio Pereira
2642	João Aristeu da Rosa
2643	Joao Baptista Baldo
2644	Joao Batista Baitello
2645	Joao Batista Calixto
2646	João Batista Camargo Júnior
2647	João Batista de Matos
2648	João Batista de Pinho
2649	Joao Batista Fernandes
2650	João Batista Garcia Canalle
2651	João Batista Lopes Martins
2652	João Batista Neves da Costa
2653	Joao Batista Ribeiro da Silva Reis - Bolsista FAPEMIG
2654	João Batista Tavares da Silva
2655	Joao Batista Teixeira da Rocha
2656	João Bento Torres Neto
2657	João Bosco Lopes Botelho
2658	Joao Bosco Paraiso da Silva
2659	João Bosco Verçosa Leal Junior
2660	João Braga
2661	Joao Candido Lima Dovicchi
2662	João Carlos Bespalhok Filho
2663	Joao Carlos Campanharo
2664	João Carlos Palazzo de Mello
2665	Joao Crisóstomo Weyl Albuquerque Costa
2666	João do Espirito Santo Batista Neto
2667	João dos Santos Carmo
2668	João Eduardo de Morais Pinto Furtado
2669	João Evangelista Steiner
2670	João Farias Rovati
2671	Joao Francisco Coelho dos Santos Junior
2672	Joao Francisco Galera Monico
2673	João Garcia Caramori Júnior
2674	Joao Gustavo Pessini Amarante Mendes
2675	Joao Henrique Ghilardi Lago
2676	João Henrique Zimnoch dos Santos
2677	João Lima Sant'Anna Neto
2678	João Luciano de Quevedo
2679	João Lúcio de Azevedo
2680	Joao Luis Callegari Lopes
2681	Joao Luiz Baptista de Carvalho
2682	João Luiz Kohl Moreira
2683	Joao Marcal Bode de Moraes
2684	Joao Marcos Bastos Cavalcanti
2685	Joao Marcos Bezerra do O
2686	João Marcos Salvi Sakamoto
2687	Joao Marcos Travassos Romano
2688	João Maria da Silva
2689	Joao Mário Santos de França
2690	João Medeiros de Araújo
2691	João Navarro Soares Júnior
2692	João Paes Vieira Sobrinho
2693	Joao Paulo de Biaso Viola
2694	João Paulo Figueiró Longo
2695	Joao Paulo Machado Torres
2696	Joao Paulo Viana Leite
2697	João Renato Stehmann
2698	João Ricardo de Freitas Oliveira
2699	Joao Roberto Moreira Neto

2700	Joao Tavares Pinho
2701	Joao Ubiratan Moreira dos Santos
2702	Joao Vasconcellos Neto
2703	Joao Vianei Soares
2704	João Victor Issler
2705	João Vital da Cunha Júnior
2706	Joao Xavier da Cruz Neto
2707	João Xavier de Araújo Júnior
2708	Joaquim Albenisio Gomes da Silveira
2709	Joaquim Carlos Rodrigues
2710	Joaquim Corsino
2711	Joaquim de Araújo Nóbrega
2712	Joaquim dos Santos
2713	Joaquim Eneas Filho
2714	Joaquim Fernando Mendes da Silva
2715	Joaquim José Barroso de Castro
2716	Joaquim José Martins Guilhoto
2717	Joaquim Manoel Goncalves
2718	Joaquim Mendes Ferreira
2719	Joaquim Procopio de Araujo Filho
2720	Jochen Junker
2721	Jochen Schongart
2722	Jociane de Carvalho Myskiw
2723	Joel Batista da Fonseca Neto
2724	Joel Buenano Macambira
2725	Joel Camara de Carvalho Filho
2726	Joel Camargo Rubim
2727	Joel Campos de Paula
2728	Joel da Silva Cordeiro
2729	Joel Machado Junior
2730	Joerg Dietrich Wilhelm Schleicher
2731	John Ethan Householder
2732	John Fontenele Araujo
2733	Johnny Ferraz Dias
2734	John Weiner
2735	Joice Mara Cruciol
2736	Joice Nunes Ferreira
2737	Joilson de Oliveira Martins
2738	Jonas Enrique Aguilar Perales
2739	Jonatas Ferreira
2740	Jonice de Oliveira Sampaio
2741	Jordan Del Nero
2742	Jorge Alberto Bustamante Becerra
2743	Jorge Alberto Martins
2744	Jorge Alexander Sosa Cardoza
2745	Jorge Arigony Neto
2746	Jorge Cesar Abrantes de Figueiredo
2747	Jorge da Silva
2748	Jorge Eduardo Lins Oliveira
2749	Jorge Ernesto Horvath
2750	Jorge Fernando Silva de Menezes
2751	Jorge Guillermo Hounie
2752	Jorge Hernandez Fernandez
2753	Jorge Iulek
2754	Jorge Ivan Rebelo Porto
2755	Jorge Juarez Vieira Teixeira
2756	Jorge Kalil
2757	Jorge Luis López Aguilar
2758	Jorge Luis Monteiro de Matos
2759	Jorge Luis Nabarrete

2760	Jorge Luis Padovan
2761	Jorge Luiz da Cunha Moraes
2762	Jorge Luiz de Almeida Ferreira
2763	Jorge Luiz e Silva
2764	Jorge Luiz Nessimian
2765	Jorge Luiz Piccinin
2766	Jorge Manuel Sotomayor Tello
2767	Jorge Marcio Ferreira Carvano
2768	Jorge Mauricio David
2769	Jorge Mendes de Oliveira-Castro Neto
2770	Jorge Neval Moll Neto
2771	Jorge Pablo Castello
2772	Jorge Paes Barreto Marcondes de Souza
2773	Jorge Passamani Zubelli
2774	Jorge Pinto Ribeiro
2775	Jorge Rady de Almeida Junior
2776	Jorge Ramiro de La Reza
2777	Jorge Ricardo Ducati
2778	Jorge Simões de Sá Martins
2779	Jorge Vicente Lopes da Silva
2780	Jorge Willian Leandro Nascimento
2781	Jorge Zaverucha
2782	Jörg Johannes Ohly
2783	Jose Ademir Sales de Lima
2784	José Agustín Pablo Quincoces Suárez
2785	José Alberto Cuminato
2786	José Alberto Fracassi da Silva
2787	Jose Alberto Giacometti
2788	José Alberto Nicolau de Oliveira
2789	José Aldemir de Oliveira
2790	Josealdo Tonholo
2791	Jose Alexandre de Jesus Perinotto
2792	José Alexandre de Souza Crippa
2793	Jose Alexandre Diniz
2794	Jose Almir Cirilo
2795	José Alves de Siqueira Filho
2796	José Alzamir Pereira da Costa
2797	José Américo de Miranda Neto
2798	José Andrés Morgado Díaz
2799	José Ângelo Rizzo
2800	José Angelo Silveira Zuanazzi
2801	Jose Antonio Alves Gomes
2802	José Antonio Aravéquia
2803	Jose Antonio Carlos Canedo Medeiros
2804	Jose Antonio de Azevedo Magalhaes
2805	Jose Antônio Frizzone
2806	Jose Antonio Hernandes
2807	José Antonio Huamaní Coaquira
2808	José Antonio Kelly Luciani
2809	Jose Antonio Marengo Orsini
2810	Jose Antonio Morais Moreira
2811	José Antônio Nunes de Mello
2812	José Antonio Picanço Diniz Junior
2813	Jose Antonio Rocha Gontijo
2814	Jose Antonio Roversi
2815	Jose Antonio Scotti Fontoura
2816	Jose Arana Varela
2817	José Augusto Baranauskas
2818	José Augusto Gomes Azevêdo
2819	Jose Augusto Jorge Rodrigues

2820	Jose Augusto Oliveira Huguenin
2821	José Augusto Paixão Veiga
2822	José Augusto Suruagy Monteiro
2823	José Barreto Campello Carvalheira
2824	José Borzacchiello da Silva
2825	Jose Camapum de Carvalho
2826	Jose Camargo da Costa
2827	José Carlos Becceneri
2828	José Carlos Bressiani
2829	Jose Carlos da Silva
2830	José Carlos Ferreira da Rocha
2831	José Carlos Gesser
2832	Jose Carlos Maldonado
2833	José Carlos Pareja
2834	José Carlos Verle Rodrigues
2835	José César Rosa
2836	Jose Cipolla Neto
2837	José Dalton Cruz Pessoa
2838	José Daniel Diniz Melo
2839	Jose Daniel Figueroa-Villar
2840	José da Rocha Carvalheiro
2841	José de Arimatéia Costa de Almeida
2842	José de Jesús Pérez Alcázar
2843	José de Jesús Rivero Oliva
2844	José de Sousa e Silva Júnior
2845	José Dias do Nascimento Júnior
2846	Jose Diomedes Barbosa Neto
2847	José Divino dos Santos
2848	Jose Djair Vendramim
2849	Jose Donizeti Alves
2850	Jose Edson Rodrigues Pereira
2851	José Eduardo Azevedo Fiates
2852	Jose Eduardo da Silveira Costa
2853	José Eduardo de Carvalho
2854	José Eduardo Levi
2855	José Eduardo Pereira Soares
2856	Jose Eduardo Telles
2857	Jose Eluf Neto
2858	Jose Ermirio Ferreira de Moraes
2859	José Exequiel Basini Rodrigues
2860	José Ferreira de Rezende
2861	Jose Francisco Comenalli Marques Junior
2862	José Francisco de Carvalho Gonçalves
2863	Jose Francisco Ribeiro
2864	José Francismar de Medeiros
2865	Josefredo Rodriguez Pliego Junior
2866	José Galberto Martins da Costa
2867	José Galizia Tundisi
2868	Jose Garcia Ribeiro Abreu Junior
2869	Jose Geraldo de Andrade Pacheco Filho
2870	Jose Gildo de Lima
2871	José Gustavo Féres
2872	Jose Hamilton Matheus Nascimento
2873	Jose Heleno Faro
2874	Jose Helio Costa
2875	Jose Henrique Muelbert
2876	José Henrique Pedrosa Macedo
2877	Jose Herskovits Norman
2878	Jose Hilton Gomes Rangel
2879	José Irineu Rangel Rigotti

2880	José Ivo Baldani
2881	Jose Jaime da Cruz
2882	José Jaime Vasconcelos Cavalcanti
2883	José Joaquim Ferreira
2884	José Joatan Rodrigues Júnior
2885	Jose Jorge de Carvalho
2886	Jose Lamartine Soares Sobrinho
2887	José Laurindo Campos dos Santos
2888	Joselene de Oliveira
2889	José Lúcio dos Santos
2890	José Luís Almada Güntzel
2891	Jose Luis Camargo Zambon
2892	José Luís Campana Camargo
2893	Jose Luiz Aarestrup Alves
2894	Jose Luiz Bezerra
2895	José Luiz de Souza Pio
2896	José Luiz Horacio Faccini
2897	José Luiz Jivago de Paula Rôlo
2898	José Luiz Lima de Azevedo
2899	Jose Luiz Rezende Pereira
2900	Jose Luiz Stech
2901	Jose Manuel Riveros Nigra
2902	Jose Marcos Andrade Figueiredo
2903	Jose Marcos Pinto da Cunha
2904	Jose Marcus de Oliveira Godoy
2905	José Maria Barbosa Filho
2906	José Maria Correia da Costa
2907	José Maria de Carvalho Filho
2908	Jose Maria de Jesus Izquierdo Villota
2909	Jose Maria de Lima
2910	José Maria Landim Dominguez
2911	José María Monserrat
2912	Jose Maria Soares Junior
2913	José Maria Villas Bôas
2914	Jose Mario Barichello
2915	Jose Mario Martinez Perez
2916	José Marques de Brito Neto
2917	Jose Maurício de Souza Campos
2918	Jose Mauricio Rosolen
2919	José Maurício Simões Bento
2920	Jose Miguel Malacarne
2921	Josemir da Cruz Alexandrino
2922	Jose Nelson dos Santos Silva Couceiro
2923	Jose Neuman de Souza
2924	José Odair Pereira
2925	Jose Osvaldo Previato
2926	José Otávio Carréra Silva Júnior
2927	Jose Palazzo Moreira de Oliveira
2928	Jose Paulo Bonatti
2929	Jose Paulo Molin
2930	Joseph Harari
2931	José Reinaldo Mendes Ruas
2932	José Reinaldo Pacheco Peleja
2933	José Renan de Medeiros
2934	José Renato Moreira da Silva de Oliveira
2935	José Ricardo Bergmann
2936	Jose Ricardo Sabino
2937	José Ricardo Siqueira
2938	José Roberto Botelho de Souza
2939	José Roberto Casarini

2940	José Roberto Castilho Piqueira
2941	José Roberto da Silva
2942	José Roberto de Souza de Almeida Leite
2943	José Roberto Figaro Caldeira
2944	José Roberto Goldim
2945	José Roberto Gonçalves de Azevedo
2946	Jose Roberto Iglesias
2947	Jose Roberto Lapa e Silva
2948	Jose Roberto Meyer Fernandes
2949	José Roberto Postali Parra
2950	Jose Ronaldo Pereira da Silva
2951	Jose Rubens Pirani
2952	Jose Salvador Lepera
2953	José Tadeu Garcia Tommaselli
2954	José Tarquinio Prisco
2955	José Vicente Elias Bernardi
2956	Jose Vicente Tavares dos Santos
2957	José Waldo Martínez Espinosa
2958	Jose Wanderley Marangon Lima
2959	Jose Wellington Rocha Tabosa
2960	Josiane Tavares de Abreu
2961	Josianne Nicácio Silveira
2962	Josias Correa de Faria
2963	Josicelia Dumet Fernandes
2964	Josiel Urbaninho de Arruda
2965	Josue Junior Guimaraes Ramos
2966	Josué Maldonado Ferreira
2967	Josué Mendes Filho
2968	Joyce Maria Annichino-Bizzacchi
2969	Jozue Vieira Filho
2970	Juan Carlos Torres Fernández
2971	Juan Clinton Llerena Junior
2972	Juan Miguel Villalobos-Salcedo
2973	Juarez Lopes Donzele
2974	Juceni Pereira de Lima David
2975	Judes Goncalves dos Santos
2976	Judith Pessoa de Andrade Feitosa
2977	Julia Clarinda Paiva Cohen
2978	Juliana Batista da Silva
2979	Juliana da Silva
2980	Juliana da Silva Bernardes
2981	Juliana de Freitas Astúa
2982	Juliana Feijó de Souza Daniel
2983	Juliana Ferreira de Souza
2984	Juliana Gonzaga Jayme
2985	Juliana Gurgel Giannetti
2986	Juliana Lopes Rangel Fietto
2987	Juliana Maldonado Marchetti
2988	Juliana Miranda da Silveira
2989	Juliana Nobre Vieira
2990	Juliana Pavan Zuliani
2991	Juliana Pereira
2992	Juliana Saraiva do Val
2993	Juliana Sartori Bonini
2994	Juliana Targino Silva Almeida e Macêdo
2995	Julianna Ferreira Cavalcanti de Albuquerque
2996	Julianne Milléo
2997	Juliano Bordignon
2998	Juliano de Carvalho Cury
2999	Juliano Ferreira

3000	Juliano Pamplona Ximenes Ponte
3001	Julieta Schachter
3002	Julio Antonio Lombardi
3003	Julio Cesar Coelho de Rose
3004	Julio Cesar de Souza Tavares
3005	Júlio Cesar Góes Ferreira
3006	Julio Cesar Jose da Silva
3007	Julio Cesar Lima D'Alge
3008	Julio Cesar Sampaio do Prado Leite
3009	Julio Cesar Santos Chagas
3010	Júlio César Sczancoski
3011	Julio Cesar Stacchini de Souza
3012	Julio Cesar Voltarelli
3013	Julio Cesar Walz
3014	Julio José Máximo de Carvalho
3015	Julio Miranda Pureza
3016	Júlio Onésio Ferreira Melo
3017	Julio Ricardo Sambrano
3018	Julio Scharfstein
3019	Jupira Gomes de Mendonça
3020	Jürgen Fritz Stilck
3021	Jussara Angélica Durães
3022	Jussara Lopes de Miranda
3023	Jussara Marques de Almeida
3024	Jussara Rocha Ferreira
3025	Kaled Dechoum
3026	Kalil Skeff Neto
3027	Kalinka Regina Lucas Jaquie Castelo Branco
3028	Karen Badaraco Costa
3029	Karen Fernandez Costa
3030	Karen Oppermann
3031	Karen Wohnrath
3032	Karina de Cássia Braga Ribeiro
3033	Karina Mayumi Tsuruta
3034	Karina Peres Gramacho
3035	Karin Argenti Simon
3036	Karin da Costa Calaza
3037	Karin do Amaral Riske
3038	Karine Bonucielli Brum
3039	Karin Koogan Breitman
3040	Karla Maria Longo de Freitas
3041	Karl Heinz Kienitz
3042	Karl-L Schuchmann
3043	Karlo Queiroz da Costa
3044	Karl Otto Stohr
3045	Kathleen Fernandes Grego
3046	Katia Borgia Barbosa Pagnano
3047	Katia Calp Gondim
3048	Katia Castanho Scortecci
3049	Kátia Cavalcanti Pôrto
3050	Kátia Cilene do Couto
3051	Katia Cristina Barbaro Nogueira
3052	Kátia De Angelis
3053	Katia Jasbinschek dos Reis Pinheiro
3054	Katia Kellem da Rosa
3055	Kátia Messias Bichinho
3056	Katia Regina dos Santos Teixeira
3057	Katia Sabrina Paludo
3058	Kátia Sento Sé Mello
3059	Katiucia Ticila de Souza Eduvirgens Ferreira

3060	Katiuscia Nadyne Cassemiro
3061	Keila Maria Mendes Ceresér
3062	Kelli Cristina Aparecida Munhoz Moreira
3063	Kelly Beatriz Vieira Torres Dozinel
3064	Kelly Cristiane da Silva
3065	Kelly Roberta Francisco Muruci de Paula
3066	Kelvin Lopes Dias
3067	Kengo Imakuma
3068	Kênia Cardoso Bícego
3069	Kenneth John Gollob
3070	Kennia Rocha Rezende
3071	Kenya Moore de Almeida Dias da Cunha
3072	Kepler de Souza Oliveira Filho
3073	Keti Tenenblat
3074	Ketty Abaroa de Rezende
3075	Kildare Rocha de Miranda
3076	Klaus Werner Capelle
3077	Kleber Augusto Lisboa Castão
3078	Kleber Campos Miranda Filho
3079	Kleber Gomes Franchini
3080	Kléber Tomás de Resende
3081	Koji Kawashita
3082	Konradin Metze
3083	Krishnamurti de Morais Carvalho
3084	Ladislau Marcelino Rabello
3085	Laécio Santos Cavalcante
3086	Laélia Pumilla Botêlho Campos dos Santos
3087	Laercio Duarte Souza
3088	Laercio Gomes
3089	Laercio Massaru Namikawa
3090	Laercio Zambolim
3091	Laerte Brandão Paes de Andrade
3092	Laerte Guimaraes Ferreira Junior
3093	Laerte Sodré Junior
3094	Laís do Nascimento Salvador
3095	Laise de Holanda Cavalcanti Andrade
3096	Laiza Canielas Krause
3097	Lalgudi Venkataraman Ramanathan
3098	Lana Lage da Gama Lima
3099	Lara Durães Sette
3100	Laura Bannach Jardim
3101	Laura Cristina Jardim Pôrto
3102	Laura Cristina Simoes Viana
3103	Laura De Simone Borma
3104	Laura Graziela Figueiredo Fernandes Gomes
3105	Laura Hecker de Carvalho
3106	Laura Helena Vega Gonzales Gil
3107	Laura Natal Rodrigues
3108	Laurent Emmanuel Dardenne
3109	Laurício Endres
3110	Lauro Antonio Saint Pastous Madureira
3111	Lauro Julio Calliari
3112	Lauro June Queiroz Maia
3113	Lauro Tatsuo Kubota
3114	Lauro Valentim Stoll Nardi
3115	Lavinia Schüler Faccini
3116	Lawrence Charles Smith
3117	Lázara Silveira Castrillo
3118	Léa Cristina de Carvalho Castellucci
3119	Lea Guimaraes Souki

3120	Léa Mirian Barbosa da Fonseca
3121	Leandra Franciscato Campo
3122	Leandra Náira Zambelli Ramalho
3123	Leandro Batista Costa
3124	Leandro Coser
3125	Leandro de Oliveira Kerber
3126	Leandro Fernandes Malloy-Diniz
3127	Leandro Franco de Souza
3128	Leandro Juen
3129	Leandro Krug Wives
3130	Leandro Leite Antonio
3131	Leandro Malard Moreira
3132	Leandro Ramos de Araujo
3133	Leandro Tasso
3134	Leandro Valle Ferreira
3135	Lêda Maria Barreto Fraga
3136	Leda Quercia Vieira
3137	Leda Satie Chubatsu
3138	Leda Viegas de Carvalho
3139	Leida Maria Botion
3140	Leila Aparecida Chiavacci
3141	Leila Beltrami Moreira
3142	Leila da Costa Ferreira
3143	Leila de Souza Fonseca
3144	Leila Maciel de Almeida e Silva
3145	Leila Maria Beltramini
3146	Leila Maria Cardao Chimelli
3147	Leila Maria Garcia Fonseca
3148	Leila Maria Moreira Beltrão Pereira
3149	Leiliane Coelho André Amorim
3150	Leizer Schnitman
3151	Lena Virginia Soares Monteiro
3152	Leni Campos Akcelrud
3153	Lenin dos Santos Pires
3154	Leo Afraneo Hartmann
3155	Leoberto Costa Tavares
3156	Leonardo Bandeira Rezende
3157	Leonardo Barbosa Koerich
3158	Leonardo Bidese de Pinho
3159	Leonardo Bruno de Sá
3160	Leonardo da Silva
3161	Leonardo De Bona Becker
3162	Leonardo de Mello Honório
3163	Leonardo de Souza Menezes
3164	Leonardo dos Santos Sena
3165	Leonardo Franklin da Costa Fontenelle
3166	Leonardo Gobbo Neto
3167	Leonardo Gregory Brunnet
3168	Leonardo José Amaral de Siqueira
3169	Leonardo Kleber Castelano
3170	Leonardo Magalhães Cruz
3171	Leonardo Magalhães Macarini
3172	Leonardo Mesquita
3173	Leonardo Pessoa Felix
3174	Leonardo Pinheiro da Silva
3175	Leonardo Régis Leira Pereira
3176	Leonardo Ribeiro de Carvalho e Fonseca
3177	Leonardo Rodrigues Araujo Xavier de Menezes
3178	Leonardo Sena Gomes Teixeira
3179	Leonardo Silva Boiteux

3180	Leonardo Varuzza
3181	Leonidas Chaves de Resende
3182	Leonilda Stanziola
3183	Leoni Villano Bonamin
3184	Leonora Mansur Mattos
3185	Leonor Costa Maia
3186	Leopoldo Pisanelli Rodrigues de Oliveira
3187	Leticia de Luna Freire
3188	Leticia Lucente Campos Rodrigues
3189	Letícia Regina de Souza Teixeira
3190	Letícia Scherer Koester
3191	Leticia Veras Costa Lotufo
3192	Lewis Joel Greene
3193	Lia Cardoso Rocha Saraiva Teixeira
3194	Liacir dos Santos Lucena
3195	Liane Marcia Rossi
3196	Lia Queiroz do Amaral
3197	Lia Rejane Muller Bevilaqua
3198	Lia Sumie Nakao
3199	Licio Augusto Velloso
3200	Lidia Andreu Guillo
3201	Lidia Moreira Lima
3202	Lidia Vasconcellos de Sá
3203	Ligia Andrade da Silva Telles Mathias
3204	Lígia Ferreira Gomes
3205	Lígia Furlan
3206	Ligia Mori Madeira
3207	Lígia Queiroz Matias
3208	Lilia Gomes Willadino
3209	Liliam Fernandes
3210	Liliana de Fatima Bezerra Lira de Pontes
3211	Liliana Yolanda Ancalla Davila
3212	Liliane dos Santos Machado
3213	Lilian Ferreira de Senna
3214	Lílian Lefol Nani Guarieiro
3215	Lilian Milnitsky Stein
3216	Lilian Noindorf
3217	Lilian Padilha
3218	Lilian Piñero Marcolin Eça
3219	Lilian Viana Teixeira
3220	Lincoln Muniz Alves
3221	Linda Viola Ehlin Caldas
3222	Lindijane de Souza Bento Almeida
3223	Lindsay Unno Gimenes
3224	Linnyer Beatrys Ruiz
3225	Lino Misoguti
3226	Lionel Fernel Gamarra Contreras
3227	Liovando Marciano da Costa
3228	Lirlândia Pires de Sousa
3229	Lisandro Juno Soares Vieira
3230	Lísia Mônica de Souza Gestinari
3231	Lisiane da Silveira Ev
3232	Lisiane dos Santos Freitas
3233	Lis Ribeiro do Valle Antonelli
3234	Liu Un Rigo
3235	Lívia Caricio Martins
3236	Livia Izabel Bezerra de Miranda
3237	Livio Amaral
3238	Lizit Alencar da Costa
3239	Liz Maria de Almeida

3240	Loana Tito Nogueira
3241	Loïc Pascal Gilles Cerf
3242	Loraine Campanati Araujo de Andrade
3243	Lorenzo Justiniano Diaz Casado
3244	Lourdes Conceição Martins
3245	Lourdes Mattos Brasil
3246	Lourenildo Williame Barbosa Leite
3247	Lourivaldo da Silva Santos
3248	Lourival Ferreira Cavalcante
3249	Luana Cassandra Breitenbach Barroso Coelho
3250	Luana Mahé Costa Gomes
3251	Lucas Antonio Miranda Ferreira
3252	Lucas Fugikawa Santos
3253	Lucas Pedreira de Carvalho
3254	Lucelia Donatti
3255	Lucélia Nobre Carvalho
3256	Luc Felicianus Marie Rouws
3257	Lúcia da Costa Ferreira
3258	Lúcia de Siqueira Campos
3259	Lucia Eilbaum
3260	Lúcia Fernanda Cavalcanti da Costa Leite
3261	Lucia Galvão de Albuquerque
3262	Lucia Garcez Lohmann
3263	Lucia Helena Boddey
3264	Lucia Helena Garófalo Chaves
3265	Lucia Libanez Bessa Campelo Braga
3266	Lúcia Maria Carareto Alves
3267	Lucia Maria Jaeger de Carvalho
3268	Lucia Maria Machado Bógus
3269	Lucia Mendonça Previato
3270	Luciana Almeida da Silva
3271	Luciana Amaral Haddad
3272	Luciana Andreia Fondazzi Martimiano
3273	Luciana Barros de Arruda
3274	Luciana Biagini Lopes
3275	Luciana Bolsoni Lourenço
3276	Luciana Castro Geraseev
3277	Luciana Chagas Caperuto
3278	Luciana Correa do Lago
3279	Luciana Costa Faria
3280	Luciana Dias Thomaz
3281	Luciana Jesus da Costa
3282	Luciana Keiko Hatamoto Zervoudakis
3283	Luciana Maria Pedreira Ramalho
3284	Luciana Pizzatti Barboza
3285	Luciana Pompeia Cavalcanti
3286	Luciana Retz de Carvalho
3287	Luciana Reyes Pires Kassab
3288	Luciana Rossini Pinto
3289	Luciana Sianto
3290	Luciana Simon Pereira Crott
3291	Luciana Teixeira de Andrade
3292	Luciana Tricai Cavalini
3293	Luciana Wasnievski da Silva
3294	Luciane Cruz Lopes
3295	Luciane Helena Gargaglioni Batalhão
3296	Luciane Maria Pereira Passaglia
3297	Luciane Pinto Gaspar
3298	Lucianna da Gama Fernandes Vieira
3299	Luciano Alves Favorito

3300	Luciano Andrade Moreira
3301	Luciano Aparecido Meireles Grillo
3302	Luciano Avallone Bueno
3303	Luciano Bastos Lopes
3304	Luciano da Fontoura Costa
3305	Luciano da Silva Cabral
3306	Luciano da Silva Souza
3307	Luciano Farias de Almeida
3308	Luciano Fernandes Huergo
3309	Luciano Gomes Fietto
3310	Luciano Hauschild
3311	Luciano Joel Fedozzi
3312	Luciano Kayser Vargas
3313	Luciano Morais Liao
3314	Luciano Nakazato
3315	Luciano Paganucci de Queiroz
3316	Luciano Pasqualoto Canellas
3317	Luciano Paulino da Silva
3318	Luciano Ponzi Pezzi
3319	Luciano Rodrigues da Silva
3320	Luciano Silva
3321	Luciano Soares da Cruz
3322	Luciano Tavares da Costa
3323	Luciano Vieira Dutra
3324	Luciano Vilela Paiva
3325	Lúcia Travassos da Rosa Costa
3326	Lúcia Vieira Santos
3327	Luci de Senna-Valle
3328	Lucídia Fonseca Santiago
3329	Luciene Alves Moreira Marques
3330	Luciene Batista da Silveira
3331	Luciene Ferreira Gaspar Amaral
3332	Lucila Helena Deliesposte Cescato
3333	Lucildes Pita Mercuri
3334	Lucilia Dias Pacobahyba
3335	Lucille Marilyn May Kriger d'Amorim Antony
3336	Lucimara Pires Martins
3337	Lucimara Stolz Roman
3338	Luci Maria Sant Ana Dusse
3339	Lucimeris Ruaro
3340	Lucindo José Quintans Júnior
3341	Lúcio Cardozo Filho
3342	Lucio Carlos Gonçalves
3343	Lucio Hora Acioli
3344	Lucio Mendes Cabral
3345	Lucio Tunes dos Santos
3346	Lucymara Fassarella Agnez
3347	Luena Nascimento Nunes Pereira
3348	Luerbio Faria
3349	Luidi Gelabert Simonetti
3350	Luis Adrian Florit
3351	Luisa Karla de Paula Arruda
3352	Luís Alberto dos Santos
3353	Luis Alberto Peche Puertas
3354	Luis Alberto Vieira de Carvalho
3355	Luis Alexandre Muehlmann
3356	Luis Alfredo Vidal de Carvalho
3357	Luisa Lina Villa
3358	Luis Antonio Brasil Kowada
3359	Luis Antônio Lacerda Aímola

3360	Luis Augusto Paim Rohde
3361	Luis Aureliano Imbiriba Silva
3362	Luis Carlos Erpen de Bona
3363	Luis Carlos Macedo Blasques
3364	Luis Carlos Trevelin
3365	Luis da Silva Zambom
3366	Luis David Solis Murgas
3367	Luis de Almeida Prado Bacellar
3368	Luis Eduardo Evangelista de Araujo
3369	Luis Eduardo Paim Rohde
3370	Luis Eduardo Soares Netto
3371	Luis Eugenio Portela Fernandes de Souza
3372	Luís Felipe Leite Martins
3373	Luis Felipe Ribeiro Pinto
3374	Luis Fernando Ayerbe
3375	Luis Fernando da Costa Medina
3376	Luis Fernando Fernandes Marins
3377	Luís Fernando Pascholati Gusmão
3378	Luis Guillermo Bahamondes
3379	Luis Gustavo Marcassa
3380	Luis Gustavo Nonato
3381	Luís Gustavo Tavares Braga
3382	Luis Henrique Bertolino Braido
3383	Luís Henrique De Barros Soares
3384	Luis Henrique Mendes da Silva
3385	Luís Marcelo Aranha Camargo
3386	Luis Parente Maia
3387	Luís Presley Serejo dos Santos
3388	Luis Raul Weber Abramo
3389	Luis Renato Bezerra Pequeno
3390	Luis Roberto Cardoso de Oliveira
3391	Luiza Antas Rabelo
3392	Luiza Carnicero de Castro
3393	Luiza Guilherme Guglielmi
3394	Luiz Alberto Amador Pereira
3395	Luiz Alberto Cury
3396	Luiz Alberto Lira Soares
3397	Luiz Alberto Macedo
3398	Luiz Alencar Reis da Silva Mello
3399	Luíza Maria de Araújo
3400	Luiz Antonio Candido
3401	Luiz Antônio da Fonseca Manso
3402	Luiz Antonio de Oliveira Nunes
3403	Luiz Antonio Ferreira Coelho
3404	Luiz Antonio Gioielli
3405	Luiz Antonio Machado da Silva
3406	Luiz Antônio Pereira Neves
3407	Luiz Antonio Ribeiro da Rosa
3408	Luiz Armando Cunha De Marco
3409	Luiza Rosaria Sousa Dias
3410	Luiz Augusto Corrêa Passos
3411	Luiz Augusto Toledo Machado
3412	Luiz Carlos Barbosa
3413	Luiz Carlos da Silveira Fontes
3414	Luiz Carlos de Sá-Rocha
3415	Luiz Carlos Dias
3416	Luiz Carlos Donizetti Goncalves
3417	Luiz Carlos Fazuoli
3418	Luiz Carlos Moreira
3419	Luiz Carlos Pacheco Rodrigues Velho

3420	Luiz Carlos Sampaio Lima
3421	Luiz Carlos Sandoval Góes
3422	Luiz Carlos Santana da Silva
3423	Luiz Cesar de Queiroz Ribeiro
3424	Luiz Claudio Costa
3425	Luiz Claudio Lazzarini de Oliveira
3426	Luiz Cláudio Meira Belo
3427	Luiz Claudio Santos Thuler
3428	Luiz Claudio Schara Magalhaes
3429	Luiz Cláudio Villar dos Santos
3430	Luiz Cosme Cotta Malaquias
3431	Luiz Davidovich
3432	Luiz de Paula do Nascimento
3433	Luiz de Sousa Santos Júnior
3434	Luiz Dione Barbosa de Melo
3435	Luiz Drude de Lacerda
3436	Luiz Eduardo de Macedo Cardoso
3437	Luiz Eduardo Maia Nery
3438	Luiz Eduardo Oliveira e Cruz de Aragão
3439	Luiz Elídio Gregório
3440	Luiz Fabrizio Stoppiglia
3441	Luiz Felipe de Souza Nobre
3442	Luiz Fernando Alves Rodrigues
3443	Luiz Fernando Dias Probst
3444	Luiz Fernando Ferrari
3445	Luiz Fernando Rocha Ferreira da Silva
3446	Luiz Fernando Teixeira Albino
3447	Luiz Fernando Wurdig Roesch
3448	Luiz Filipe Protasio Pereira
3449	Luiz Francisco Ditzel Faraco
3450	Luiz Gilberto Barreta
3451	Luiz Gonçalves Neto
3452	Luiz Gonzaga de Castro Junior
3453	Luiz Gonzaga de França Lopes
3454	Luiz Guilherme Machado de Macedo
3455	Luiz Gustavo Almeida Martins
3456	Luiz Gustavo de Oliveira Lopes Cançado
3457	Luiz Henrique Amaral
3458	Luiz Henrique Carvalho Batista
3459	Luiz Henrique Florindo
3460	Luiz Henrique Rosa
3461	Luiz Hildebrando Pereira da Silva
3462	Luiz Juliano Neto
3463	Luiz Marcos da Fonseca
3464	Luiz Mariano Paes de Carvalho Filho
3465	Luiz O M Kobayashi
3466	Luiz Orlando Ladeira
3467	Luiz Oswaldo Carneiro Rodrigues
3468	Luiz Paulo Kowalski
3469	Luiz Paulo Ribeiro Vaz
3470	Luiz Pereira Ramos
3471	Luiz Pinguelli Rosa
3472	Luiz Rijo
3473	Luiz Roberto Guimarães Guilherme
3474	Luiz Roberto Leão Ferreira
3475	Luiz Roberto Nunes
3476	Luiz Roberto Ribeiro Castello Branco
3477	Luiz Satoru Ochi
3478	Luiz Sidney Longo Junior
3479	Luiz Tadeu Moraes Figueiredo

3480	Luiz Valcov Loureiro
3481	Luiz Vicente Rizzo
3482	Luke Thomas Wyn Parry
3483	Luquesio Petrola de Melo Jorge
3484	Luz Adriana Cuartas Pineda
3485	Luzia Kalyne Almeida Moreira Leal
3486	Luzineide Wanderley Tinoco
3487	Lycia Mara Jenné Mimica
3488	Lygia da Veiga Pereira
3489	Lygia Maria Friche Passos
3490	Lys Mary Bileski Cândido
3491	Madalena Carneiro da Cunha Areias
3492	Magali Benjamim de Araújo
3493	Magaly Girão Albuquerque
3494	Magda Fraguas Serra
3495	Magdala de Araujo Novaes
3496	Magda Lahorgue Nunes
3497	Magda Maria Bello de Almeida Neves
3498	Magna Suzana Alexandre Moreira
3499	Magnolia de Araujo Campos PfenninG
3500	Magnos Martinello
3501	Magnus Ake Gidlund
3502	Mahir Saleh Hussein
3503	Maique Weber Biavatti
3504	Maíra Baumgarten Corrêa
3505	Maira Galdino da Rocha Pitta
3506	Maira Graeff Burin
3507	Maira Martins da Silva
3508	Maísa Ribeiro Pereira Lima Brigagão
3509	Manfredo Perdigao do Carmo
3510	Manfred Willy Müller
3511	Mangalathayil Ali Abdu
3512	Manoela Ferreira Fernandes da Silva
3513	Manoel Barral Netto
3514	Manoel Bezerra Campêlo Neto
3515	Manoel do Couto Fernandes
3516	Manoel Fernandes Martins Nogueira
3517	Manoel Ferreira Cardoso
3518	Manoel Gomes de Mendonça Neto
3519	Manoel Jacobsen Teixeira
3520	Manoel Martins Dias Filho
3521	Manoel Odorico de Moraes Filho
3522	Manoel Pereira Filho
3523	Manoel Ribeiro Filho
3524	Manoel Silva de Vasconcelos
3525	Manoel Souza D'Agrella Filho
3526	Manoel Valnir Júnior
3527	Manuela dos Santos Carvalho
3528	Manuel de Jesus Simões
3529	Manuel Simoes Filho
3530	Mara Rejane Ritter
3531	Mara Silvia Pinheiro Arruda
3532	Marbey Manhães Mosso
3533	Marcal Jose Rodrigues Pires
3534	Marccus Vinícius da Silva Alves
3535	Marcela de Freitas Lopes
3536	Marcela Elena Fejes
3537	Marcele Regine de Carvalho
3538	Marcelino Pereira dos Santos Silva
3539	Marcella Barbosa Faria de Almeida Prado

3540	Marcella Beraldo de Oliveira
3541	Marcello Garcia Trevisan
3542	Marcello Luiz Rodrigues de Campos
3543	Marcello Thiry Comicholi da Costa
3544	Marcellus Henrique Loiola Ponte de Souza
3545	Marcelo Addas de Carvalho
3546	Marcelo Afonso Vallim
3547	Marcelo Alex de Carvalho
3548	Marcelo Alves Soares
3549	Marcelo Amorim Savi
3550	Marcelo Andrade Macêdo
3551	Marcelo Antonio Pavanello
3552	Marcelo Areias Trindade
3553	Marcelo Augusto Gutierrez Carnelossi
3554	Marcelo Banik de Pádua
3555	Marcelo Barbio Rosa
3556	Marcelo Bastos Mazza
3557	Marcelo Bittencourt Ivair Pinto
3558	Marcelo Borges Mansur
3559	Marcelo Braga dos Santos
3560	Marcelo Byrro Ribeiro
3561	Marcelo Cohen
3562	Marcelo Corrêa Bernardes
3563	Marcelo Daniel Brito Faria
3564	Marcelo da Rosa Alexandre
3565	Marcelo de Andrade Ferreira
3566	Marcelo de Castro Costa
3567	Marcelo de Macedo Brigido
3568	Marcelo de Oliveira Gonzaga
3569	Marcelo de Oliveira Johann
3570	Marcelo de Oliveira Terra Cunha
3571	Marcelo de Pádula
3572	Marcelo Dias Baruffi
3573	Marcelo dos Santos
3574	Marcelo dos Santos Guerra Filho
3575	Marcelo Eduardo Huguenin Maia da Costa
3576	Marcelo Ehlers Loureiro
3577	Marcelo Einicker Lamas
3578	Marcelo Emilio
3579	Marcelo Esteves Almeida
3580	Marcelo Farina
3581	Marcelo Fernandes Camargos
3582	Marcelo Frota Benvenuti
3583	Marcelo Galeazzi Caxambú
3584	Marcelo Ganzarolli de Oliveira
3585	Marcelo Gattass
3586	Marcelo Giordan Santos
3587	Marcelo Gustavo Lorenzo
3588	Marcelo Henrique dos Santos
3589	Marcelo Henrique Gehlen
3590	Marcelo Henrique Mamede Lewer
3591	Marcelo Henrique Ongaro Pinheiro
3592	Marcelo Henrique Sousa
3593	Marcelo Hermes Lima
3594	Marcelo Lacerda Vasquez
3595	Marcelo Leite Lyra
3596	Marcelo Luís Francisco Abbade
3597	Marcelo Magalhães Fares Saba
3598	Marcelo Martinelli
3599	Marcelo Martins de Sena

3600	Marcelo Medeiros Guimarães
3601	Marcelo Menin
3602	Marcelo Menossi Teixeira
3603	Marcelo Miranda Viana da Silva
3604	Marcelo Moizinho Oliveira
3605	Marcelo Müller dos Santos
3606	Marcelo Navarro
3607	Marcelo Nazareno Vallinoto de Souza
3608	Marcelo Ornaghi Orlandi
3609	Marcelo Paleologo Elefteriadis de França Santos
3610	Marcelo Peres Rocha
3611	Marcelo Queiroz Hoexter
3612	Marcelo Resende de Souza
3613	Marcelo Ribeiro de Araújo
3614	Marcelo Rosado Fantappié
3615	Marcelo Salabert Gonzalez
3616	Marcelo Sampaio
3617	Marcelo Sandin Dourado
3618	Marcelo Santos
3619	Marcelo Santos Castilho
3620	Marcelo Silva Pinho
3621	Marcelo Silva Sarandy
3622	Marcelo Soares Lubaszewski
3623	Marcelo Sobral da Silva
3624	Marcelo Sousa de Assumpção
3625	Marcelo Tabarelli
3626	Marcelo Teixeira Rodrigues
3627	Marcelo Torres Bozza
3628	Marcelo Yoshimoto
3629	Marcelo Zaldini Hernandes
3630	Marcelo Zoéga Maialle
3631	Marcelo Zugaib
3632	Marcel Vinicius Medeiros Oliveira
3633	Márcia Akemi Yamasoe
3634	Márcia Almeida de Araújo Alexandre
3635	Marcia Almeida de Melo
3636	Marcia Aparecida de Sant'Ana Barros
3637	Marcia Attias
3638	Márcia Caruso Bícego
3639	Marcia Chame
3640	Marcia Consentino Kronka Sosthenes
3641	Marcia Cristina Bernardes Barbosa
3642	Márcia Cristina Costa de Azevedo
3643	Márcia Cristina França Ferreira
3644	Marcia Cristina Paes
3645	Marcia da Silva Pereira Leite
3646	Márcia de Freitas Lenzi
3647	Marcia Gomes de Oliveira
3648	Marcia Gomide da Silva Mello
3649	Marcia Grisotti
3650	Marcia Helena Costa Fampa
3651	Marcia Helena Miranda Cardoso Podestá
3652	Márcia Kauer Sant´Anna
3653	Marcia Laudelina Arruda Temperini
3654	Marcia Lorena Fagundes Chaves
3655	Márcia Maria Barros dos Passos
3656	Márcia Maria de Oliveira Bezerra
3657	Márcia Maria de Souza
3658	Marcia Maria Rippel
3659	Marcia Marques Gomes

3660	Marcia Mendonca Carneiro
3661	Márcia Messias da Silva
3662	Márcia Motta Maués
3663	Márcia Muller
3664	Marcia Paranho Veloso
3665	Márcia Queiroz Latorraca
3666	Márcia Regina Calderipe Farias Rufino
3667	Marcia Regina Machado dos Santos
3668	Márcia Regina Pereira Attie
3669	Marcia Regina Piuvezam
3670	Marcia Rodrigues Amorim dos Santos
3671	Márcia Rosana Cerioli
3672	Márcia Soares Vidal
3673	Márcia Tsuyama Escote
3674	Márcia Vetromilla Fuentes
3675	Marcilio Sérgio Soares da Cunha Filho
3676	Marcio Andre Rodrigues Cavalcanti de Alencar
3677	Marcio Arab Murad
3678	Marcio Argollo Ferreira de Menezes
3679	Marcio Assolin Corrêa
3680	Márcio Augusto Ernesto de Moraes
3681	Márcio Botelho de Castro
3682	Marcio Cherem Schneider
3683	Márcio das Virgens Rebouças
3684	Marcio de Castro Silva Filho
3685	Márcio de Matos Coelho
3686	Márcio de Oliveira Barros
3687	Márcio De Paula Filgueiras
3688	Marcio de Souza Soares de Almeida
3689	Marcio Dias Santos
3690	Márcio Eduardo Delamaro
3691	Márcio Eduardo Kreutz
3692	Marcio Flavio Dutra Moraes
3693	Marcio Gilberto Cardoso Costa
3694	Marcio Gomes Soares
3695	Marcio Jose Coelho de Pontes
3696	Marcio José Poças Fonseca
3697	Márcio Lazzarotto
3698	Márcio Lopes Cornélio
3699	Marcio Luiz de Oliveira
3700	Márcio Luiz dos Santos
3701	Marcio Luiz Moore Nucci
3702	Marcio Machado Ladeira
3703	Márcio Martins Pimentel
3704	Marcio Merino Fernandes
3705	Marcio Moraes Valenca
3706	Marcio Murilo Barboza Tenorio
3707	Márcio Pereira da Rocha
3708	Márcio Reis Custódio
3709	Marcio Roberto Costa Martins
3710	Marcio Roberto Teixeira Nunes
3711	Márcio Roberto Viana dos Santos
3712	Marcio Rocha Francelino
3713	Marcio Sarroglia Pinho
3714	Márcio Weber Paixão
3715	Marcius da Silva Almeida
3716	Marco Alberto Medeiros
3717	Marco Andre Urbach Mezzasalma
3718	Marco Antonio Alves Brasil
3719	Marco Antonio Casanova

3720	Marco Antonio Cesar Bonomo
3721	Marco Antonio Chaer Nascimento
3722	Marco Antônio da Silva Campos
3723	Marco Antonio da Silva Mello
3724	Marco Antonio Ferreira Randi
3725	Marco Antonio Galarza Toro
3726	Marco Antonio Gaya de Figueiredo
3727	Marco Antonio Gutierrez
3728	Marco Antonio Nogueira
3729	Marco Antônio Peliky Fontes
3730	Marco Antonio Perruso
3731	Marco Antônio Pinheiro de Cristo
3732	Marco Antonio Silveira
3733	Marco Antonio Stefani
3734	Marco Antonio Stephano
3735	Marco Antônio Vasconcelos Rêgo
3736	Marco Antonio Zago
3737	Marco Aurelio Cavalcanti Pacheco
3738	Marco Aurélio de Moura Freire
3739	Marco Aurélio de Oliveira Schroeder
3740	Marco Aurélio de Sousa Lacerda
3741	Marco Aurelio do Rego Monteiro
3742	Marco Aurélio Ferreira
3743	Marco Aurélio Gonçalves Ferreira
3744	Marco Aurelio Krieger
3745	Marco Aurélio Martins
3746	Marco Aurelio Romano-Silva
3747	Marco Aurelio Takita
3748	Marco Aurelio Zezzi Arruda
3749	Marco Cremona
3750	Marco Flôres Ferrão
3751	Marco José de Sousa
3752	Marcondes Lima da Costa
3753	Marconi Gomes da Silva
3754	Marcos Abdo Arbex
3755	Marcos Alexandre Castilho
3756	Marcos André Barros Galhardo
3757	Marcos André de Carvalho
3758	Marcos André Gonçalves
3759	Marcos Andre Vannier dos Santos
3760	Marcos Antonio Couto dos Santos
3761	Marcos Antonio Ruggieri Franco
3762	Marcos Antônio Soares
3763	Marcos Antonio Tambascia
3764	Marcos Antonio Zen Vasconcellos
3765	Marcos Assuncao Pimenta
3766	Marcos Barbosa Ferreira
3767	Marcos Bryan Heinemann
3768	Marcos César Alvarez
3769	Marcos Cesar de Oliveira
3770	Marcos Daisuke Oyama
3771	Marcos Dajczer
3772	Marcos da Silva Freire
3773	Marcos David Ferreira
3774	Marcos de Almeida Bezerra
3775	Marcos Duarte Maia
3776	Marcos Eduardo Coutinho
3777	Marcos Felipe Silva de Sa
3778	Marcos Heil Costa
3779	Marcos Henrique Degani

3780	Marcos Henrique Ferreira Sorgine
3781	Marcos Hiroiuqui Kunita
3782	Marcos Horacio Pereira
3783	Marcos Inácio Marcondes
3784	Marcos Jose Salgado Vital
3785	Marcos José Santana
3786	Marcos Lázaro Moreli
3787	Marcos Lordello Chaim
3788	Marcos Luciano Bruschi
3789	Marcos Massao Futai
3790	Marcos Massi
3791	Marcos Nogueira Eberlin
3792	Marcos Paulo Fuck
3793	Marcos Perez Diaz
3794	Marcos Pérsio Dantas Santos
3795	Marcos Rincon Voelzke
3796	Marcos Roberto de Mattos Fontes
3797	Marcos Rogério Tótola
3798	Marcos Silveira
3799	Marcos Silveira Buckeridge
3800	Marcos Venício Alves Lima
3801	Marcos Vinicius Dias Vermelho
3802	Marcos Vinicius Folegatti
3803	Marcos Welby Correa Silva
3804	Marco Tadeu Grassi
3805	Marco Túllio Menna Barreto de Vilhena
3806	Marc Roger Jean Marie Henry
3807	Marcus Alberto Nadruz Coelho
3808	Marcus Fernandes de Oliveira
3809	Marcus Mandolesi Sá
3810	Marcus Peigas Pacheco
3811	Marcus Polette
3812	Marcus Theodor Schilling
3813	Marcus Vinicius Aparecido Gomes de Lima
3814	Marcus Vinicius Bahia
3815	Marcus Vinicius Batistuta
3816	Marcus Vinicius Fontana Copetti
3817	Marcus Vinicius Gomez
3818	Marcus Vinícius Guimarães de Lacerda
3819	Marcus Vinicius Sampaio
3820	Marcus Vinicius Sarkis Martins
3821	Marcus Vinícius Teixeira Navarro
3822	Marcus Vinícius Vaughan Jennings Licínio
3823	Mareni Rocha Farias
3824	Margaret Cristina da Silva Boguszewski
3825	Margarete Cristiane de Costa Trindade Amorim
3826	Margarete de Macedo Monteiro
3827	Margaret Haiganouch Magdesian
3828	Margareth Castro Ozelo
3829	Margareth Cizuka Toyama Udo
3830	Margareth de Fátima Formiga Melo Diniz
3831	Margareth de Lara Capurro-Guimarães
3832	Maria Alice dos Santos Alves
3833	Maria Alice Neves
3834	Maria Amélia de Sousa Mascena Veras
3835	Maria Amélia Novais Schleicher
3836	Maria Angela de Almeida Souza
3837	Maria Angelica Miglino
3838	Maria Angélica Pereira de Carvalho Costa
3839	Maria Angélica Vergara Wasserman

3840	Maria Anita Mendes
3841	Maria Antonieta da Conceição Rodrigues
3842	Maria Antonieta Longo Galvão da Silva
3843	Maria Antonieta Parahyba Leopoldi
3844	Maria Aparecida Azevedo Pereira da Silva
3845	Maria Aparecida de Jesus
3846	Maria Aparecida Godoy Soler Pajanian
3847	Maria Aparecida Juliano
3848	Maria Aparecida Neves Jardini
3849	Maria Aparecida Soares Ruas
3850	Maria Aparecida Zaghete Bertochi
3851	Maria Augusta Borges Cursino de Freitas Arruda
3852	Maria Auxiliadora Fortini Veloso
3853	Maria Bellio
3854	Maria Berenice Reynaud Steffens
3855	Maria Bernadete de Sousa Maia
3856	Maria Bernadete Riemma Pierre
3857	Maria Betânia Galvão dos Santos Freire
3858	Maria Candida Henrique Mamede
3859	Maria Carolina da Silva Leme
3860	Maria Carolina Nemes
3861	Maria Cascao Ferreira de Almeida
3862	Maria Cecilia Bevilacqua
3863	Maria Cecilia Calani Baranauskas
3864	Maria Cecilia de Souza Minayo
3865	Maria Cecília Fonsêca Azoubel
3866	Maria Celi Ramos da Cruz Scalon
3867	Maria Celuta Machado Viana
3868	Maria Christina Fialho de Mello
3869	Maria Claudia Costa Irigoyen
3870	Maria Claudia França da Cunha Felinto
3871	Maria Cleide Carlos Bernal
3872	Maria Clorinda Soares Fioravanti
3873	Maria Conceicao do Rosário
3874	Maria Conceição Soares Meneses Lage
3875	Maria Cristina Canela
3876	Maria Cristina de Oliveira Izar
3877	Maria Cristina Dias Lay
3878	Maria Cristina Fedrizzi
3879	Maria Cristina Ferreira de Oliveira
3880	Maria Cristina Forti
3881	Maria Cristina Machado Motta
3882	Maria Cristina Marcucci Ribeiro
3883	Maria Cristina Nonato
3884	Maria Cristina Silva Boeres
3885	Maria Cristina Soares Guimarães
3886	Maria Cristina Solci
3887	Maria Cristina Thomaz
3888	Maria Cristina Trindade Terra
3889	Maria Crystianne Fonseca Rosal
3890	Maria Cynthia Braga
3891	Maria da Conceição Klaus Vanderley Ramos
3892	Maria da Conceicao Nascimento Costa
3893	Maria da Glória Lima Cruz Teixeira
3894	Maria da Graca Campos Pimentel
3895	Maria da Graça Figueiredo Pereira Dutra
3896	Maria da Graça Morais Martin
3897	Maria da Graca Naffah Mazzacoratti
3898	Maria da Graça Nascimento
3899	Maria da Paz Lima

3900	Maria da Penha Albuquerque Potiens
3901	Maria das Graças Almeida
3902	Maria das Graças Andrade Korn
3903	Maria das Gracas Carvalho
3904	Maria das Gracas Leopardi Goncalves
3905	Maria das Gracas Machado de Souza
3906	Maria das Gracas Muller de Oliveira Henriques
3907	Maria de Fátima Agra
3908	Maria de Fatima Andrade
3909	Maria de Fatima de Albuquerque Rangel Moreira
3910	Maria de Fátima Dias Costa
3911	Maria de Fátima Leite
3912	Maria de Fátima M Almeida Santos
3913	Maria de Fátima Oliveira
3914	Maria de Fátima Oliveira Saraiva
3915	Maria de Fátima Vanderlei de Souza
3916	Maria de Jesus Coutinho Varejão
3917	Maria de los Angeles Gasalla
3918	Maria de Lourdes Correa Figueiredo
3919	Maria de Lourdes da Costa Soares Morais
3920	Maria de Lourdes Leite de Moraes
3921	Maria de Lourdes Moreira
3922	Maria de Lourdes Pinheiro Ruivo
3923	Maria de Lourdes Teixeira de Moraes Polizeli
3924	Maria de Nazaré do Carmo Bastos
3925	Maria Denise Feder
3926	Maria do Carmo Alberto-Rincon
3927	Maria do Carmo Alves de Lima
3928	Maria do Carmo Esteves da Costa
3929	Maria do Carmo Gonçalves
3930	Maria do Carmo Hespanhol da Silva
3931	Maria do Carmo Rangel Santos Varela
3932	Maria do Carmo Ruaro Peralba
3933	Maria do Livramento Miranda Clementino
3934	Maria Domingues Vargas
3935	Maria do Rosario Dias de Oliveira Latorre
3936	Maria Dorothea Post Darella
3937	Maria do Socorro Nogueira
3938	Maria do Socorro Pombo-de-Oliveira
3939	Maria Dulce Picanço Bentes Sobrinha
3940	Maria Edileuza Felinto de Brito
3941	Maria Elisa Zanella
3942	Maria Elizabeth Bandeira-Pedrosa
3943	Maria Elvira Pizzigatti Correa
3944	María Esperanza Cortés Segura
3945	Maria Esther de Noronha Fonseca
3946	Maria Esther Soares Marques
3947	Maria Eulalia Vares
3948	Maria Fernanda do Carmo Gurgel
3949	Maria Fernanda Pimentel Avelar
3950	Maria Fernanda Tourinho Peres
3951	Maria Florice Raposo Pereira
3952	Maria Gabriela Martin Ávila
3953	Maria Goreti de Almeida Oliveira
3954	Maria Goreti Rodrigues Vale
3955	Maria Guadalupe Medina
3956	Maria Helena Bezerra Maia de Hollanda
3957	Maria Helena Gaíva Gomes-da-Silva
3958	Maria Helena Ortolan Matos
3959	Maria Ignez Capella Gaspar Elsas

3960	Maria Ignez Leão
3961	Maria Ilma Andrade Santos Araujo
3962	Maria Ines Basso Bernardi
3963	Maria Ines Costa Dourado
3964	Maria Inês da Rosa
3965	Maria Inês de Toledo
3966	Maria Ines Rocha Miritello Santoro
3967	Maria Inês Schmidt
3968	Maria Inês Smiljanic Borges
3969	Maria Iracema Bezerra Loiola
3970	Maria Irma Seixas Duarte
3971	Maria Isabel Alves de Souza Waddington Achatz
3972	Maria Isabel Felisberti
3973	Maria Isabel Pereira Vianna
3974	Maria Isabel Sobral Escada
3975	Maria Izabel Gallão
3976	Maria Jaqueline Vasconcelos
3977	Maria José Araújo Sales
3978	Maria José Baldessar
3979	Maria Jose Campagnole dos Santos
3980	Maria José Carvalho Carmona
3981	Maria José do Nascimento Ferreira
3982	Maria Josefina Gabriel Sant'Anna
3983	Maria José Martins Duarte Osis
3984	Maria Jose Pacifico
3985	Maria José Pontes
3986	Maria José Soares Mendes Giannini
3987	Maria Laene Moreira de Carvalho
3988	Maria Letícia Cintra
3989	Maria Lidia Medeiros Vignol Lelarge
3990	Maria Lourdes Barjas-Castro
3991	Maria Lourdes Farre Vallve
3992	Maria Lourdes Peris Barbo
3993	Maria Luísa Penteado Natividade Targon
3994	Maria Luiza Franceschi Nicodemo
3995	Maria Luiza Garnelo Pereira
3996	Maria Luiza Macedo Silva
3997	Maria Luiza Videira Marceliano
3998	Maria Madalena Rinaldi
3999	Maria Manuela da Fonseca Moura
4000	Maria Marluce dos Santos Vilela
4001	Maria Marta Loddi
4002	Maria Martha Bernardi
4003	Maria Martha Campos
4004	Maria Martha Costa Hübner
4005	Mariana Lima Vale
4006	Mariana Matera Veras
4007	Maria Nazareth Ferreira da Silva
4008	Mariane Campelo Koslinski
4009	Mariane Rembold Petraglia
4010	Mariângela Cristofani-Yaly
4011	Mariângela de Oliveira Abans
4012	Mariangela Macchione
4013	Mariângela Menezes
4014	Marianna Vieira Sobral Castello Branco
4015	Marianne Pinotti
4016	Mariano Francisco Laplane
4017	Mariano Gustavo Zalis
4018	Maria Olívia Amado Ramos Bacellar
4019	Maria Osvalneide Lucena Sousa

4020	Maria Palmira Daflon Gremião
4021	Maria Paula Curado
4022	Maria Paula Gomes Mourão
4023	Maria Raquel Alcântara de Miranda
4024	Maria Regina Alves Cardoso
4025	Maria Regina de Vasconcellos Barbosa
4026	Maria Regina Fernandes de Oliveira
4027	Maria Rejane Souza de Britto Lyra
4028	Maria Risoleta Freire Marques
4029	Maria Rita de Morais Chaves Santos
4030	Maria Rita dos Santos e Passos Bueno
4031	Maria Rita Rodrigues
4032	Maria Salete Marcon Gomes Vaz
4033	Maria Salete Martins
4034	María Segunda Aurora Prado
4035	Maria Stela Grossi Porto
4036	Maria Stella Coutinho de Alcantara Gil
4037	Maria Suely Soares Leonart
4038	Maria Sylvia Silva Dantas
4039	Maria Teresa Anselmo Olinto
4040	Maria Teresa Fernandez Piedade
4041	Maria Teresa Pepato
4042	Maria Teresa Salles Trevisan
4043	Maria Teresa Vieira Sanseverino
4044	Maria Tereza de Araujo
4045	Maria Tereza dos Santos Correia
4046	Maria Tereza Ferreira Duenhas Monreal
4047	Maria Tereza Nunes
4048	Maria Urbana Pinto Brandão Rondon
4049	Maria Valdrinez Campana Lonardoni
4050	Maria Victoria Ramos Ballester
4051	Maria Virginia Petry
4052	Maria Vitória Lopes Badra Bentley
4053	Maria Zélia Aguiar de Sousa
4054	Marie-Anne Van Sluys
4055	Marilda de Souza Gonçalves
4056	Marilda Mendonça Guazzelli Ramos Vianna
4057	Marildo Geraldête Pereira
4058	Marileia Scartezini
4059	Marilene Demasi
4060	Marilene Hohmuth Lopes
4061	Marília Caixeta Franco Ariosa
4062	Marília Dalva Turchi
4063	Marília Jobim Sartori
4064	Marilia Junqueira Caldas
4065	Marilia Lopes da Costa Faco Soares
4066	Marilia Martins Melo
4067	Marilia Sa Carvalho
4068	Marília Zaluar Passos Guimarães
4069	Marilis Dallarmi Miguel
4070	Marilvia Dansa de Alencar
4071	Marina de Fátima de Sá Rebelo
4072	Marina Franco Maggi Tavares
4073	Marina Galdino da Rocha Pitta
4074	Marinaldo Ferreira Pinto
4075	Marina Rodrigues de Aguiar
4076	Marinella Machado Araujo
4077	Marinês Bastianel
4078	Marinêz Isaac Marques
4079	Marino Muxfeldt Bianchin

4080	Mario Alberto Cardoso da Silva Neto
4081	Mario Antonio Ribeiro Dantas
4082	Mario Augusto Gonçalves Jardim
4083	Mário Cesar do Nascimento Bevilaqua
4084	Mario Cesar Ugulino de Araujo
4085	Mario Cilense
4086	Mario Cohn-Haft
4087	Mario Engelsberg
4088	Mario Ernesto Giroldo Valerio
4089	Mario Fonseca Paulino
4090	Mario Godinho Junior
4091	Mario Hiroyuki Hirata
4092	Mário Jorge Dias Carneiro
4093	Mario Jose de Oliveira
4094	Mário Lopes da Silva Júnior
4095	Mário Lúcio Vilela de Resende
4096	Mário Luís Ribeiro Cesaretti
4097	Mario Luiz Chizzotti
4098	Mário Luiz Gomes Soares
4099	Mario Olimpio de Menezes
4100	Mário Pereira da Silva
4101	Mario Ricardo Gongora Rubio
4102	Mario Roberto Folhadela Benevides
4103	Mario Roberto Meneghetti
4104	Mario Sergio de Carvalho Mazzoni
4105	Mario Sergio Palma
4106	Marisa Carvalho Suarez
4107	Marisa Cristina Guimaraes Rocha
4108	Marisa do Espírito Santo Borin
4109	Marisa Dolhnikoff
4110	Marisa Helena Gennari de Medeiros
4111	Marisa Narciso Fernandes
4112	Marisa Ramos Barbieri
4113	Marisa Rangel
4114	Marisa Semprini
4115	Marisa Vieira de Queiroz
4116	Marise Pinheiro Nunes
4117	Maristela Delgado Orellana
4118	Maristela Pereira
4119	Marivaldo dos Santos Nascimento
4120	Marivânia Conceição de Araujo
4121	Marize Campos Valadares Bozinis
4122	Marize Varella de Oliveira
4123	Mariz Vainzof
4124	Markus Endler
4125	Marlene Benchimol
4126	Marlete Pereira Meira de Assuncao
4127	Marley Maria Bernardes Rebuzzi Vellasco
4128	Marlia Regina Coelho-Ferreira
4129	Marli Gerenutti
4130	Marli Pires Morim
4131	Marlise Miriam de Matos Almeida
4132	Marliton Rocha Barreto
4133	Marlos Alves Bezerra
4134	Marlos Pereira de Araujo Goes
4135	Marlucia Bonifacio Martins
4136	Marly Babinski
4137	Marne Carvalho de Vasconcellos
4138	Marnio Teixeira-Pinto
4139	Marrielle Maia Alves Ferreira

4140	Marson Bruck Warpechowski
4141	Marta Cristina Corsi de Filippi
4142	Marta de Azevedo Irving
4143	Marta do Nascimento Cordeiro
4144	Marta Margarete Cestari
4145	Marta Maria Cassiano
4146	Marta Maria de Franca Fonteles
4147	Marta Maria Duarte Carvalho Vila
4148	Marta Maria Menezes Bezerra Duarte
4149	Marta Marques de Souza
4150	Marta Teresa da Silva Arretche
4151	Martha Meriwether Sorenson
4152	Martha Simões Ribeiro
4153	Martin Alejandro Musicante
4154	Martin Christoph Curi Spörl
4155	Martin Eduardo Poletti
4156	Martinho de Almeida e Silva
4157	Martín Pablo Cammarota
4158	Martin Roffé
4159	Martin Tygel
4160	Márvio Lobão Teixeira de Abreu
4161	Mary Anne Heidi Dolder
4162	Maryland Sanchez Lacerda
4163	Marysabel Pinto Telis Silveira
4164	Marystela Ferreira
4165	Massayoshi Yoshida
4166	Mateus Batistella
4167	Matheus de Freitas Fernandes Pedrosa
4168	Matheus Lavorenti Rocha
4169	Mathias Henrique Weber
4170	Matthias Rudolf Brust
4171	Mauricio Almeida Noernberg
4172	Mauricio Cunha Escarpinati
4173	Mauricio de Souza Bologna
4174	Maurício Kunz
4175	Maurício Lacerda Nogueira
4176	Mauricio Lanznaster
4177	Mauricio Lima Barreto
4178	Mauricio Lisboa Nobre
4179	Mauricio Magalhães Mata
4180	Mauricio Martins Rodrigues
4181	Mauricio Massazumi Oka
4182	Mauricio Moraes Victor
4183	Mauricio Ortiz Calvao
4184	Mauricio Pamplona Pires
4185	Mauricio Porto Pato
4186	Mauricio Reis Bogo
4187	Maurício Ribeiro Baldan
4188	Mauricio Santana Moreau
4189	Mauricio Silva de Lima
4190	Mauricio Simoes Abrao
4191	Mauricio Veloso Brant Pinheiro
4192	Maurício Vicente Donadon
4193	Mauricio Yonamine
4194	Mauro Aquiles La Scalea
4195	Mauro Carlos Costa Ribeiro
4196	Mauro Cesar Geraldes
4197	Mauro Cirano
4198	Mauro Coelho dos Santos
4199	Mauro Copelli Lopes da Silva

4200	Mauro de Lima Santos
4201	Mauro Eloi Nappo
4202	Mauro José Costa Salles
4203	Mauro Martins Teixeira
4204	Mauro Pereira Soares
4205	Mauro Rocha Côrtes
4206	Mauro Silveira de Castro
4207	Mauro Sola-Penna
4208	Mauro Zackiewicz
4209	Maximiliano Loiola Ponte de Souza
4210	Maximiliano Luis Faundez-Abans
4211	Maximiliano Luis Munford
4212	Max Mauro Dias Santos
4213	Mayana Zatz
4214	Mayra Kassawara Martins
4215	Melissa Ameloti Gomes Avelino
4216	Melissa de Mattos Pimenta
4217	Melissa Izabel Hannas
4218	Mercedes Maria da Cunha Bustamante
4219	Mércia de Sousa Galvão
4220	Mércia Liane de Oliveira
4221	Mércia Tancredo Toledo
4222	Messias Guimarães Bandeira
4223	Michael Anthony Stanton
4224	Michael John Brennan
4225	Michelangelo Durazzo
4226	Michele Christine Landemberger
4227	Michele Tomoko Sato
4228	Michele Vitolo
4229	Michel Georges Albert Vincentz
4230	Michel Michaelovitch de Mahiques
4231	Michel Misse
4232	Miguel Alfredo Carid Naveira
4233	Miguel Angelo Laporta Nicolelis
4234	Miguel Angelo Martins Moreira
4235	Miguel Ângelo Nobre e Souza
4236	Miguel Angelo Stipp Basei
4237	Miguel Ángel Ramírez Gil
4238	Miguel Franklin de Castro
4239	Miguel Gustavo de Campos Batista
4240	Miguel Murat Vasconcellos
4241	Miguel Soriano Balparda Caro
4242	Milane de Souza Leite
4243	Miled Hassan Youssef Moussa
4244	Militao Vieira Figueredo
4245	Milton da Costa Lopes Filho
4246	Milton José Porsani
4247	Milton Kampel
4248	Milton Luiz Horn Vieira
4249	Milton Nascimento da Silva
4250	Milton Pires Ramos
4251	Milton Roque Bugs
4252	Mirella Moura Moro
4253	Miriam de Barcellos Falkenberg
4254	Miriam Dupas Hubinger
4255	Miriam Furtado Hartung
4256	Miriam Garcia Mijares
4257	Miriam Lemos
4258	Miriam Pillar Grossi
4259	Míriam Silva Rafael

4260	Miriana da Silva Machado
4261	Miriani Griselda Pastoriza
4262	Mirian Marubayashi Hidalgo
4263	Mirian Parente Monteiro
4264	Mirian Ribeiro Leite Moura
4265	Mirian Salvador
4266	Mirna Wetters Portuguez
4267	Mitermayer Galvão dos Reis
4268	Mitre Costa Dourado
4269	Mitsue Haraguchi
4270	Moacir de Souza Dias Junior
4271	Moacir José Buenano Macambira
4272	Moacir Lacerda
4273	Moacir Rossi Forim
4274	Moacyr Cunha de Araujo Filho
4275	Moacyr Jesus Barreto de Melo Rêgo
4276	Moema Ferreira Soares Britto
4277	Mohammed Saad Lahlou
4278	Mohand Benachour
4279	Moises Goldbaum
4280	Moisés Renato Nunes Ribeiro
4281	Moises Vidal Ribeiro
4282	Monalisa Sampaio Carneiro
4283	Moni Behar
4284	Mônica Angélica Varella Petti
4285	Mônica Aragona
4286	Mônica Barbosa de Melo
4287	Monica Costa Padilha
4288	Mônica Cristina de Oliveira
4289	Monica da Costa Pereira Lavalle Heilbron
4290	Mônica de Avelar Figueiredo Mafra Magalhães
4291	Monica de Mesquita Lacerda
4292	Mônica Desiderio
4293	Monica Dias de Souza
4294	Mônica Ferreira Moreira Carvalho Cardoso
4295	Monica Fontes Caetano
4296	Mônica Freiman de Souza Ramos
4297	Monica Giannoccaro Von Huelsen
4298	Monica Jones Costa
4299	Monica Lucia Gomes
4300	Monica Parente Ramos
4301	Monica Pereira Garcia
4302	Mônica Regina da Costa Marques
4303	Mônica Ryff Moreira Roca Vianna
4304	Monica Stropa Ferreira Nozawa
4305	Monica Talarico Duailibi
4306	Monique Azevedo Esperidião
4307	Moysés Alberto Navarro
4308	Moysés Szklo
4309	Mozart Neves Ramos
4310	Mucio Flavio Barbosa Ribeiro
4311	Munir Salomao Skaf
4312	Murilo Francisco Tome
4313	Murilo Rezende Melo
4314	Myanna Hvid Lahsen
4315	Myrna Cristina Bonaldo
4316	Nabor da Silveira Pio
4317	Nadia Monesi
4318	Nadir Francisca Sant'Anna
4319	Nadja Cristhina de Souza Pinto

4320	Nadja Schroder
4321	Nadja Simão Magalhães
4322	Nadya Araujo Guimarães
4323	Nágila Maria Pontes Silva Ricardo
4324	Nairam Félix de Barros
4325	Nair Honda Kawashita
4326	Nair Otaviano Aguiar
4327	Najeh Maissar Khalil
4328	Nalayne Mendonça Pinto
4329	Nance Beyer Nardi
4330	Nanci do Nascimento
4331	Nancy das Gracas Cardia
4332	Nancy dos Santos Barbi
4333	Nancy Mieko Abe
4334	Nara Lins Meira Quintão
4335	Narendra Narain
4336	Narrúbia Oliveira de Almeida
4337	Natália Gindri Fiorenza
4338	Natalia Rezende Landin
4339	Natália Valenga Parizotto
4340	Natáli Valim Oliver Bento Torres
4341	Nathalie de Lourdes Souza Dewulf
4342	Nathan Bessa Viana
4343	Nathan Jacob Berkovits
4344	Naylor Bastiani Perez
4345	Nazareth Rocha de Magalhaes Barros
4346	Neemias Alves de Lima
4347	Neftalí Lenin Villarreal Carreño
4348	Neidenêi Gomes Ferreira
4349	Neife Lilian Zalloum
4350	Nei Pereira Junior
4351	Neiva Vieira da Cunha
4352	Nelder de Figueiredo Gontijo
4353	Neliana Buzi Figlie
4354	Nelilma Correia Romeiro
4355	Nelio Alessandro Azevedo Cacho
4356	Nelma Regina Segnini Bossolan
4357	Nelson Albuquerque de Souza e Silva
4358	Nelson Carlos Rosot
4359	Nelson Eduardo Duran Caballero
4360	Nelson Giordano Delgado
4361	Nelson Goldenstein
4362	Nelson Horacio Gabilan
4363	Nelson Luis Saldanha da Fonseca
4364	Nelson Maculan Filho
4365	Nelson Rodrigo da Silva Martins
4366	Nelson Studart Filho
4367	Nelson Vani Leister
4368	Nelson Velho de Castro Faria
4369	Nelson Wanderley Perioto
4370	Nelson Yoshihiro Nakajima
4371	Nereide Stela Santos Magalhães
4372	Neres de Lourdes da Rosa Bitencourt
4373	Neri dos Santos
4374	Nestor Cortez Saavedra Filho
4375	Neusa de Lima Nogueira
4376	Neusa Hamada
4377	Neusa Maria Paes Leme
4378	Neuza Maria de Castro
4379	Newton Andréo Filho

4380	Newton Cesario Frateschi
4381	Newton de Figueiredo Filho
4382	Newton Gonçalves de Castro
4383	Newton Martins Barbosa Neto
4384	Newton Paulo de Souza Falcao
4385	Ney Laert Vilar Calazans
4386	Nicim Zagury
4387	Nicolas Carels
4388	Nicolaus Albert Borges Schriefer
4389	Nicolina Silvana Romano Lieber
4390	Niede Guidon
4391	Niédson José da Silva
4392	Niege Araçari Jacometti Cardoso Furtado
4393	Niels Olsen Saraiva Câmara
4394	Nikifor Rakov Gomez
4395	Niklaus Ursus Wetter
4396	Nikolai Alexandrovitch Goussevskii
4397	Nilce Santos de Melo
4398	Nilda Berenice de Vargas Barbosa
4399	Nildo da Silva Dias
4400	Nilo Cesar Consoli
4401	Nils Erik Svensjö
4402	Nilson Costa Roberty
4403	Nilson Dias Vieira Junior
4404	Nilson Nunes Tavares
4405	Nilson Sant'Anna
4406	Nilton Alves de Rezende
4407	Nilton Itiro Morimoto
4408	Nilton Rosembach Junior
4409	Nilva Kazue Sakomura
4410	Nilza Pires
4411	Niraldo Paulino
4412	Niro Higuchi
4413	Nisia Krusche
4414	Nito Angelo Debacher
4415	Nivaldo Costa Muniz
4416	Nivaldo da Silva
4417	Nivaldo Lucio Speziali
4418	Nivaldo Silveira Ferreira
4419	Nivan Bezerra da Costa Jr
4420	Nivio Ziviani
4421	Nobuo Oki
4422	Noemi Costa dos Santos
4423	Noemi de La Rocque Rodriguez
4424	Norberto Dani
4425	Norberto Mario Rodriguez
4426	Norberto Peporine Lopes
4427	Norma Gouvêa Rumjanek
4428	Norton Gomes de Almeida
4429	Nylane Maria Nunes de Alencar
4430	Obdulio Gomes Miguel
4431	Octavio Augusto Ceva Antunes
4432	Octavio Rossi de Morais
4433	Odair Correa Bueno
4434	Odair Giraldin
4435	Odair Pastor Ferreira
4436	Odilon Giovannini Junior
4437	Odilon Gomes Pereira
4438	Odylio Denys de Aguiar
4439	Ohara Augusto

4440	Olaf Malm
4441	Olavo de Faria Galvão
4442	Oldair Donizeti Leite
4443	Oleg Vladimirovich Krasilnikov
4444	Olga Balachova
4445	Olga Lúcia Castreghini de Freitas Firkowski
4446	Olga Nikolaevna Goussevskaia
4447	Olga Regina Pereira Bellon
4448	Olga Tiemi Sato
4449	Olga Zazuco Higa
4450	Olimpio Hiroshi Miyagaki
4451	Olinda Maria Gomes da Costa Vilas Boas
4452	Olivar Antônio Lima de Lima
4453	Omar Paranaiba Vilela Neto
4454	Onofre Trindade Junior
4455	Orides Morandin Junior
4456	Oriel Herrera Bonilla
4457	Orlando Alves dos Santos Junior
4458	Orlando David Henrique dos Santos
4459	Osame Kinouchi Filho
4460	Osamu Saotome
4461	Oscar Calavia Saez
4462	Oscar Endrigo Dorneles Rodrigues
4463	Oscar Jesus Choque Fernandez
4464	Oscar Manoel Loureiro Malta
4465	Oscar Nassif de Mesquita
4466	Osman Rosso Nelson
4467	Osmar Abílio de Carvalho Junior
4468	Osmar Avanzi
4469	Osmar Malaspina
4470	Osmar Olinto Möller Junior
4471	Osmar Pinto Junior
4472	Osvaldo Albuquerque Cavalcanti
4473	Osvaldo Antonio Serra
4474	Osvaldo Augusto Brazil Esteves Sant'Anna
4475	Osvaldo Chiavone Filho
4476	Osvaldo de Lazaro Casagrande Junior
4477	Osvaldo Ferrarese-Filho
4478	Osvaldo Massaiti Takayanagui
4479	Osvaldo Novais de Oliveira Junior
4480	Osvaldo Rettore Neto
4481	Oswaldo Luiz Alves
4482	Otávio Berwanger
4483	Otavio Costa Acevedo
4484	Otavio Mitio Ohashi
4485	Othon Cabo Winter
4486	Otília Deusdênia Loiola Pessoa
4487	Otoni Cardoso do Vale
4488	Otto Mack Junqueira
4489	Pablo Agustin Vargas
4490	Pablo Alejandro Fiorito
4491	Pablo Augusto Ferrari
4492	Pablo Elías Martínez
4493	Pablo Javier Grunmann
4494	Pablo Jenner Paredez Angeles
4495	Pablo Machado
4496	Pablo Riul
4497	Pabricio Marcos Oliveira Lopes
4498	Palloma Vieira Muterlle
4499	Paloma Lys de Medeiros

4500	Paola Corio
4501	Paola Zucchi
4502	Paolo Bartolini
4503	Paolo Crivelli
4504	Paolo Di Mascio
4505	Patricia Andreia Paiola Scalco
4506	Patricia Angélica Alves Marques
4507	Patricia Ashton Prolla
4508	Patrícia Benedini Martelli
4509	Patricia Coelho de Soárez
4510	Patricia Constantino
4511	Patricia Cristina Baleeiro Beltrão Braga
4512	Patricia da Silva Melo
4513	Patrícia de Araujo Brandão Couto
4514	Patricia de Azambuja Penna
4515	Patricia Dias Fernandes
4516	Patricia Duarte de Lima Machado
4517	Patricia Fernanda do Pinho
4518	Patricia Fonseca Ferreira Arienti
4519	Patricia Franca Gardino
4520	Patricia Gomes Cardoso
4521	Patricia Klarmann Ziegelmann
4522	Patricia Lustoza de Souza
4523	Patricia Machado Rodrigues e Silva Martins
4524	Patrícia Maria Albuquerque de Farias
4525	Patricia Maria Bergamo Favaro
4526	Patrícia Maria Guedes Paiva
4527	Patricia Maria Melo Sampaio
4528	Patricia Melchionna Albuquerque
4529	Patrícia Melo Aguiar
4530	Patricia Moriel
4531	Patrícia Nicolucci
4532	Patrícia Pestana Garcez
4533	Patricia Pintor dos Reis
4534	Patrícia Pommé Confessori Sartoratto
4535	Patricia Rieken Macedo Rocco
4536	Patricia Rosa Vanderborght
4537	Patricia Sampaio Tavares Veras
4538	Patricia Sartorelli
4539	Patricia Savio de Araujo Souza
4540	Patrícia Silva Lessa
4541	Patricia Souza dos Santos
4542	Patricia Teresa Monteiro Cunningham
4543	Patricia Torres Bozza
4544	Patrícia Waltz Schelini
4545	Patrícia Zancan
4546	Patrízia Raggi Abdallah
4547	Paula Adriane Perez Ribeiro
4548	Paula Debert
4549	Paula Diniz Galera
4550	Paula Fernandes de Brito
4551	Paul Alexander Schweitzer
4552	Paula Montero
4553	Paula Nishiyama
4554	Paula Regina Costa Ribeiro
4555	Paula Regina Fortes
4556	Paula Rodrigues Teixeira Coelho
4557	Paulina Maria Maia Barbosa
4558	Paulo Afonso de André
4559	Paulo Afranio Augusto Lopes

4560	Paulo Americo Maia Neto
4561	Paulo Anselmo Ziani Suarez
4562	Paulo Antunes Horta Junior
4563	Paulo Arruda
4564	Paulo Augusto Berquo de Sampaio
4565	Paulo Augusto de Lima Pontes
4566	Paulo Augusto Nepomuceno Garcia
4567	Paulo Benigno Pena Batista
4568	Paulo Borges Rodrigues
4569	Paulo Caramelli
4570	Paulo Cavalcanti Gomes Ferreira
4571	Paulo Celso Pardi
4572	Paulo César Brustolini
4573	Paulo César da Rocha Poppe
4574	Paulo Cesar de Carvalho Alves
4575	Paulo Cesar de Jesus
4576	Paulo Cesar de Lima Nogueira
4577	Paulo Cesar de Morais
4578	Paulo César Mappa
4579	Paulo Cesar Masiero
4580	Paulo Cesar Meletti
4581	Paulo Cesar Muniz de Lacerda Miranda
4582	Paulo Cesar Serafini
4583	Paulo Cezar Gomes
4584	Paulo Cezar Vieira
4585	Paulo da Cunha Lana
4586	Paulo de Barros Correia
4587	Paulo de Figueiredo Pires
4588	Paulo de Tarso Cavalcante Freire
4589	Paulo Domingos Cordaro
4590	Paulo Dornelles Picon
4591	Paulo dos Santos Pompeu
4592	Paulo Eduardo Artaxo Netto
4593	Paulo Eduardo Fornasari Farinas
4594	Paulo Eduardo Martins Ribolla
4595	Paulo Eduardo Mayorga Borges
4596	Paulo Eduardo Narcizo de Souza
4597	Paulo Emílio Lovato
4598	Paulo Estefano Dineli Bobrowiec
4599	Paulo Estevão Cruvinel
4600	Paulo Eurico Pires Ferreira Travassos
4601	Paulo Eustáquio Duarte Pinto
4602	Paulo Fernando Ferreira Frutuoso e Melo
4603	Paulo Fernando Papaleo Fichtner
4604	Paulo Firmino Moreira Junior
4605	Paulo Gabriel Hilu da Rocha Pinto
4606	Paulo Giácomo Milani
4607	Paulo Henrique Gorgatti Zarbin
4608	Paulo Henrique Labiak Evangelista
4609	Paulo Henrique Menezes da Silva
4610	Paulo Henrique Monteiro Borba
4611	Paulo Henrique Portela de Carvalho
4612	Paulo Henrique Schneider
4613	Paulo Henrique Souto Ribeiro
4614	Paulo Hilário Nascimento Saldiva
4615	Paulo Ivo Homem de Bittencourt Júnior
4616	Paulo Justiniano Ribeiro Junior
4617	Paulo Lee Ho
4618	Paulo Márcio da Silveira
4619	Paulo Marcio Leal de Menezes

4620	Paulo Marcondes Carvalho Junior
4621	Paulo Marcos Fernandes Boa Sorte
4622	Paulo Mascarello Bisch
4623	Paulo Maurício Lima de Alencastro Graça
4624	Paulo Mazzafera
4625	Paulo Mazzoncini de Azevedo Marques
4626	Paulo Mota Bandarra
4627	Paulo Murilo Castro de Oliveira
4628	Paulo Negrais Carneiro Seabra
4629	Paulo Negreiros de Figueiredo
4630	Paulo Nobre
4631	Paulo Pereira Christo
4632	Paulo Rebelles Reis
4633	Paulo Renato Zuquim Antas
4634	Paulo Ricardo Garcia Fernandes
4635	Paulo Ricardo Petter Medeiros
4636	Paulo Roberto Adona
4637	Paulo Roberto Bueno
4638	Paulo Roberto Caixeta Junior
4639	Paulo Roberto Costa
4640	Paulo Roberto de Lima Lopes
4641	Paulo Roberto Ferreira Louzada Junior
4642	Paulo Roberto Grossi Sad
4643	Paulo Roberto Lima Machado
4644	Paulo Roberto Mei
4645	Paulo Roberto Oliveira
4646	Paulo Roberto Ribeiro Costa
4647	Paulo Roberto Rodrigues Soares
4648	Paulo Roberto Stefani Sanches
4649	Paulo Rogério Carrara Couto
4650	Paulo Roney Kilpp Goulart
4651	Paulo Rossi Junior
4652	Paulo Rubens Guimarães Barrocas
4653	Paulo Seleghim Júnior
4654	Paulo Sérgio Bernarde
4655	Paulo Sérgio Cugnasca
4656	Paulo Sérgio de Moraes Sarmento Pinheiro
4657	Paulo Sérgio de Paula Herrmann Junior
4658	Paulo Sergio de Paula Vargas
4659	Paulo Sergio de Sousa Gorayeb
4660	Paulo Sérgio Lacerda Beirão
4661	Paulo Sérgio Lopes de Souza
4662	Paulo Sergio Lourenço de Freitas
4663	Paulo Sergio Marcellini
4664	Paulo Sérgio Martins de Carvalho
4665	Paulo Sergio Silva Rodrigues
4666	Paulo Sérgio Soares Guimarães
4667	Paulo Sergio Varoto
4668	Paulo Silva Belmonte de Abreu
4669	Paulo Simionatto Polito
4670	Paulo Tácito Gontijo Guimarães
4671	Paulo Takeo Sano
4672	Paulo Teixeira de Sousa Jr
4673	Paulo Wilson Mauriz
4674	Paulo Yoshio Kubota
4675	Pedro Afonso de Paula Pereira
4676	Pedro Cavalcanti Gomes Ferreira
4677	Pedro Dantas Fernandes
4678	Pedro de Alcantara dos Santos Neto
4679	Pedro de Lima Neto

4680	Pedro de Magalhães Padilha
4681	Pedro Fernandes Ribeiro Neto
4682	Pedro Fernando da Costa Vasconcelos
4683	Pedro Francisco Giavina-Bianchi Júnior
4684	Pedro Geraldo Pascutti
4685	Pedro Gomes de Alvarenga
4686	Pedro Heitor Barros Geraldo
4687	Pedro Jorge Caldas Magalhães
4688	Pedro José de Castro
4689	Pedro José Rolim Neto
4690	Pedro Lagerblad de Oliveira
4691	Pedro Leite da Silva Dias
4692	Pedro Leme Silva
4693	Pedro Luis Grande
4694	Pedro Luiz Guzzo
4695	Pedro Manuel Calas Lopes Pacheco
4696	Pedro Marcos Gomes Soares
4697	Pedro Michaluart Junior
4698	Pedro Miguel Ocampos Pedroso
4699	Pedro Moacyr Pinto Coelho Mota
4700	Pedro Muanis Persechini
4701	Pedro Paglione
4702	Pedro Paulo Chieffi
4703	Pedro Paulo de Mello Venezuela
4704	Pedro Paulo Martins de Oliveira
4705	Pedro Paulo Thiago de Mello
4706	Pedro Roitman
4707	Pedro Ros Petrovick
4708	Pedro Takao Yamamoto
4709	Pedro Teixeira Lacava
4710	Pedro Veiga Rodrigues Paulino
4711	Pedro Vieira da Silva Magalhães
4712	Pedro Vitoriano de Oliveira
4713	Pedro Yoshito Noritomi
4714	Péricles Assad Hassun Filho
4715	Perola de Castro Vasconcellos
4716	Pérola de Oliveira Magalhães Dias Batista
4717	Person Pereira Neves
4718	Peter Herman May
4719	Peter Jürgen Tatsch
4720	Peter Lees Pearson
4721	Peter Mann de Toledo
4722	Petrônio Filgueiras de Athayde Filho
4723	Petrus Agrippino de Alcantara Junior
4724	Petrus D Amorim Santa Cruz Oliveira
4725	Phabyanno Rodrigues Lima
4726	Philip Martin Fearnside
4727	Pierre Girard
4728	Pierre Kaufmann
4729	Pierre Mothé Esteves
4730	Pieter Willem Westera
4731	Pietro Sampaio Baruselli
4732	Pio Colepicolo Neto
4733	Plínio Barbosa de Camargo
4734	Plínio Carlos Alvalá
4735	Plinio Delatorre
4736	Plinio de Sa Leitao Junior
4737	Plinio Ricardo Ganime Alves
4738	Poliane Alfenas Zerbini
4739	Poli Mara Spritzer

4740	Polinaya Muralikrishna
4741	Polyana Albino Silva Machado
4742	Pompeu Pereira de Abreu Filho
4743	Poty Rodrigues de Lucena
4744	Primavera Borelli
4745	Priscila Braun-Grabolle
4746	Priscila Faulhaber Barbosa
4747	Priscila Gava Mazzola
4748	Priscila Machado Vieira Lima
4749	Priscila Rohem dos Santos
4750	Priscila Vanessa Zabala Capriles Goliatt
4751	Priscila Vieira Rosa
4752	Priscilla Christina Olsen
4753	Quezia Bezerra Cass
4754	Qu Fanyao
4755	Rachel Oliveira Castilho
4756	Rafaela Campostrini Forzza
4757	Rafaela Fernandes da Silva
4758	Rafael Andrade
4759	Rafael Castro de Andrade
4760	Rafael de Almeida Tubino
4761	Rafael de Paiva Salomão
4762	Rafael Dhalia
4763	Rafael Dias Mesquita
4764	Rafael Duarte Coelho dos Santos
4765	Rafael Haag
4766	Rafael José de Menezes Bastos
4767	Rafaella Costa Bonugli Santos
4768	Rafael Malagoli Rocha
4769	Rafael Mazer Etto
4770	Rafael Medeiros Sperb
4771	Rafael Roesler
4772	Rafael Sfair de Oliveira
4773	Rafael Soares de Arruda
4774	Rafael Soares Gonçalves
4775	Rafael Stelmach
4776	Rafael Trevisan
4777	Rafael Werneck Cinoto
4778	Raigna Augusta da Silva Zadra Armond
4779	Railda Shelsea Taveira Rocha do Nascimento
4780	Raimunda Liége Souza de Abreu
4781	Raimunda Nonata Ribeiro Sampaio
4782	Raimundo Carlos Silvério Freire
4783	Raimundo Cosme de Oliveira Junior
4784	Raimundo da Silva Barreto
4785	Raimundo Lopes de Oliveira Filho
4786	Raimundo Netuno Nobre Villas
4787	Raimundo Nonato Tavora Costa
4788	Raimundo Ribeiro Passos
4789	Raimundo Rocha dos Santos
4790	Raimundo Rodrigues Gomes Filho
4791	Raimundo Silva Junior
4792	Rajendra Mohan Srivastava
4793	Ralf Gielow
4794	Ralph Santos-Oliveira
4795	Ramachrisna Teixeira
4796	Ramon Alfredo Moreno
4797	Ranyere Silva Nóbrega
4798	Raphael Bragança Alves Fernandes
4799	Raquel Cardoso de Melo Minardi

4800	Raquel Carvalho Montenegro
4801	Raquel Ciuvalschi Maia
4802	Raquel Fernanda Gerlach
4803	Raquel Gouvêa Dos Santos
4804	Raquel Kely Bortoleto Bugs
4805	Raquel Luciana Boscariol Camargo
4806	Raquel Maria de Melo
4807	Raquel Maria de Oliveira
4808	Raquel Oliveira Prates
4809	Raquel Paiva Dias-Scopel
4810	Raquel Regina Duarte Moreira
4811	Raquel Rolnik
4812	Raquel Santini Leandro Rade
4813	Raquel Santos Mauler
4814	Raquel Wiggers
4815	Raul Antonino Feijóo
4816	Raul Jose Donangelo
4817	Raul Narciso Carvalho Guedes
4818	Raul Nunes de Carvalho Junior
4819	Raúl Oscar Vallejos
4820	Raymundo Baptista
4821	Regiani Carvalho de Oliveira
4822	Regina Aparecida Correia Gonçalves
4823	Regina Bitelli Medeiros
4824	Regina Borges de Araujo
4825	Regina Célia Cussa Kubrusly
4826	Regina Célia dos Santos Alvalá
4827	Regina Célia Garcia de Andrade
4828	Regina Celia Mello Santiago Moises
4829	Regina Célia Mingroni Netto
4830	Regina Celia Monteiro de Paula
4831	Regina Celia Viana Martins da Silva
4832	Regina Celi Costa Luizao
4833	Regina Coeli dos Santos Goldenberg
4834	Regina Helena Carlucci Santana
4835	Regina Helena Costa Queiroz
4836	Regina Helena da Silva
4837	Reginaldo Carmello Corrêa de Moraes
4838	Reginaldo Mattar Nasser
4839	Reginaldo Muccillo
4840	Reginaldo Palazzo Junior
4841	Reginaldo Sabóia de Paiva
4842	Reginaldo Sérgio Pereira
4843	Regina Lucia Teixeira Mendes da Fonseca
4844	Regina Mara Silva Pereira
4845	Regina Maria Barretto Cicarelli
4846	Regina Maria de Aquino
4847	Regina Oliveira da Silva
4848	Regina Pekelmann Markus
4849	Regina Sandra Veiga Nascimento
4850	Regina Terumi Yamaki
4851	Regis Munhoz Kras Borges
4852	Reinaldo Barreto Oriá
4853	Reinaldo Bertola Cantarutti
4854	Reinaldo Calixto de Campos
4855	Reinaldo Camino Bazito
4856	Reinaldo Francisco Ferreira Lourival
4857	Reinaldo Giudici
4858	Reinaldo Imbrozio Barbosa
4859	Reinaldo Nobrega de Almeida

4860	Reinaldo Oliveira Vianna
4861	Reinaldo Ramos de Carvalho
4862	Reinhardt Adolfo Fuck
4863	Rejane Corrêa Marques
4864	Rejane Jurema Mansur Custódio Nogueira
4865	Remis Balaniuk
4866	Renan Pedra de Souza
4867	Renata Barbosa de Oliveira
4868	Renata Binato Gomes
4869	Renata da Silva Schmitt
4870	Renata Fonseca Vianna Lopez
4871	Renata Galante
4872	Renata Gonçalves Ferreira
4873	Renata Lèbre La Rovere
4874	Renata Mendes de Araujo
4875	Renata Pardini
4876	Renata Pontin de Mattos Fortes
4877	Renata Schama Lellis
4878	Renata Weissmann Borges Mendonça
4879	Renato Antonio Celso Ferreira
4880	Renato Augusto DaMatta
4881	Renato Beozzo Bassanezi
4882	Renato Bortoloti
4883	Renato Camargo Giacomini
4884	Renato Campello Cordeiro
4885	Renato da Silva Carreira
4886	Renato da Veiga Guadagnin
4887	Renato David Ghisolfi
4888	Renato de Azevedo Tribuzy
4889	Renato de Figueiredo Jardim
4890	Renato de Lima Santos
4891	Renato de Mello-Silva
4892	Renato de Oliveira Resende
4893	Renato Evangelista de Araujo
4894	Renato Felix Nunes
4895	Renato Fileto
4896	Renato Fontes Guimarães
4897	Renato Fontoura de Gusmão Cerqueira
4898	Renato Luis Furlan
4899	Renato Luiz Prado
4900	Renato Mendes Guimarães
4901	Renato Moreira Angelo
4902	Renato Moreira Rosa
4903	Renato Paiva
4904	Renato Raul Boschi
4905	Renato Sanches Freire
4906	Renato Sergio Balao Cordeiro
4907	Renato Sergio Dallaqua
4908	Renato Sérgio de Lima
4909	René Alfonso Nome Silva
4910	René Duarte Martins
4911	René Rodrigues
4912	Renio dos Santos Mendes
4913	Renner de Souza Leite
4914	Reuven Opher
4915	Rex Antonio da Costa Medeiros
4916	Reynaldo Daniel Pinto
4917	Reynaldo Luiz Victoria
4918	Reynam da Cruz Pestana
4919	Ricardo Alaggio Ribeiro

4920	Ricardo Alberto Neto Ferreira
4921	Ricardo Alexandre Amar de Aguiar
4922	Ricardo Alexandre Cavalcanti de Lima
4923	Ricardo Amaral Contaifer
4924	Ricardo Ambrósio Fock
4925	Ricardo Andrade Rebelo
4926	Ricardo Andrade Reis
4927	Ricardo Antônio Amaral de Lemos
4928	Ricardo Antonio de Castro Pereira
4929	Ricardo Antonio De Simone Zanon
4930	Ricardo Arlen Buriti da Costa
4931	Ricardo Arraes de Alencar Ximenes
4932	Ricardo Augusto da Luz Reis
4933	Ricardo Augusto de Melo Reis
4934	Ricardo Augusto de Oliveira
4935	Ricardo Azevedo da Silva
4936	Ricardo Bentes de Azevedo
4937	Ricardo Bicca de Alencastro
4938	Ricardo Caetano Azevedo Biloti
4939	Ricardo Carvalho de Barros
4940	Ricardo Cordeiro Corrêa
4941	Ricardo da Silva Torres
4942	Ricardo de Andrade Medronho
4943	Ricardo de Araujo Kalid
4944	Ricardo de Camargo
4945	Ricardo de Carvalho Ferreira
4946	Ricardo de Freitas Lima
4947	Ricardo De Marco
4948	Ricardo de Oliveira Anido
4949	Ricardo de Oliveira Duarte
4950	Ricardo de Oliveira Souza
4951	Ricardo de Souza Kuchenbecker
4952	Ricardo de Souza Secco
4953	Ricardo Dias Silva
4954	Ricardo Elesbão Alves
4955	Ricardo Elgul Samad
4956	Ricardo Emmanuel de Souza
4957	Ricardo Erthal Santelli
4958	Ricardo Ferreira Bento
4959	Ricardo Ferreira Monteiro
4960	Ricardo Gomes da Rosa
4961	Ricardo Ivan Ferreira da Trindade
4962	Ricardo Jorge Klitzke
4963	Ricardo José Alves
4964	Ricardo José Gunski
4965	Ricardo Lima de Castro
4966	Ricardo Lima Guimarães
4967	Ricardo Luiz Longo
4968	Ricardo Machado Ruiz
4969	Ricardo Machado Xavier
4970	Ricardo Magnus Osorio Galvao
4971	Ricardo Mario Arida
4972	Ricardo Marques Ribeiro
4973	Ricardo Menegatti
4974	Ricardo Neves Marreto
4975	Ricardo Oliveira Freire
4976	Ricardo Paulino Marques
4977	Ricardo Pezzuol Jacobi
4978	Ricardo Pietrobon
4979	Ricardo Pimenta Bertolla

4980	Ricardo Pinto de Medeiros
4981	Ricardo Radighieri Rascado
4982	Ricardo Rego Bordalo Correia
4983	Ricardo Reis e Silva
4984	Ricardo Ruther
4985	Ricardo Sá Earp
4986	Ricardo Saldanha Honorato
4987	Ricardo Santiago Gomez
4988	Ricardo Tostes Gazzinelli
4989	Ricardo Vieira
4990	Ricardo Wagner Nunes
4991	Richard Charles Garratt
4992	Richard Hemmi Valente
4993	Richard John Ward
4994	Richard Ricachenevsky Gurski
4995	Rielva Solimairy Campelo do Nascimento
4996	Rilene Ferreira Diniz Valadares
4997	Rilke Tadeu Fonseca de Freitas
4998	Rinaldo Wellerson Pereira
4999	Rita Baltazar de Lima
5000	Rita de Cássia Araújo Pereira
5001	Rita de Cássia Barradas Barata
5002	Rita de Cássia da Conceição Gomes
5003	Rita de Cassia Fernandes de Lima
5004	Rita de Cassia Guimaraes Mesquita
5005	Rita de Cássia Martins Montezuma
5006	Rita de Cássia Pinheiro Machado
5007	Rita de Cássia Ruiz
5008	Rita Flavia Miranda de Oliveira Donzele
5009	Rita Goreti Amaral
5010	Rita Marcia da Silva Pinto
5011	Rita Maria de Brito Alves
5012	Rita Valeria Andreoli de Souza
5013	Rita Yuri Ynoue
5014	Rivaldo Niero
5015	Rivelilson Mendes de Freitas
5016	Rivelino Martins Cavalcante
5017	Roberta de Souza Coelho
5018	Roberta Mary Vidotti
5019	Roberta Monterazzo Cysneiros
5020	Robert Michael Boddey
5021	Roberto Araújo de Oliveira Santos Júnior
5022	Roberto Barbosa Bazotte
5023	Roberto Bechara Muniz
5024	Roberto César Pereira Lima Júnior
5025	Roberto Cid Fernandes Junior
5026	Roberto Coury Pedrosa
5027	Roberto Dall'Agnol
5028	Roberto Dell'Aglio Dias da Costa
5029	Roberto de Vasconcelos Antunes
5030	Roberto Eduardo Bittar
5031	Roberto Fernandes Silva Andrade
5032	Roberto Fernandes Tavares Filho
5033	Roberto Fernando da Fonseca Lyra
5034	Roberto Fernando de Souza
5035	Roberto Francisco Di Lorenzo
5036	Roberto Gil Annes da Silva
5037	Roberto Giugliani
5038	Roberto Guardani
5039	Roberto Guarniero

5040	Roberto Hideaki Tsunaki
5041	Roberto Hugo Bielschowsky
5042	Roberto Imbuzeiro Moraes Felinto de Oliveira
5043	Roberto Kant de Lima
5044	Roberto Kenji Nakamura Cuman
5045	Roberto Kopke Salinas
5046	Roberto Lent
5047	Roberto Luiz da Cunha Barroso Ramos
5048	Roberto Luiz do Carmo
5049	Roberto Luiz Moreira
5050	Roberto Manuel Torresi
5051	Roberto Martins de Souza
5052	Roberto Mauricio Carvalho Guedes
5053	Roberto Max de Argollo
5054	Roberto Mendes Finzi Neto
5055	Roberto Mendonca Faria
5056	Roberto Menezes Serra
5057	Roberto Ortiz
5058	Roberto Passetto Falcao
5059	Roberto Pedroso de Oliveira
5060	Roberto Perez Xavier
5061	Roberto Pontarolo
5062	Roberto Rezende
5063	Roberto Ricardo Panepucci
5064	Roberto Schirru
5065	Roberto Silva Sarthour Junior
5066	Roberto Soares de Castro
5067	Roberto Tadeu Raittz
5068	Roberto Takashi Sudo
5069	Roberto Ventura Santos
5070	Roberto Vieira Martins
5071	Roberto Vilela Veloso
5072	Roberto Willrich
5073	Roberto Yuji Tanaka
5074	Roberto Zilles
5075	Robert Ronald Maguiña Zamora
5076	Roberval Stefani
5077	Robledo de Almeida Torres
5078	Robson Augusto Souza dos Santos
5079	Robson Coutinho Silva
5080	Robson de Queiroz Monteiro
5081	Robson dos Santos
5082	Robson Nunes de Lima
5083	Robson Rogerio Cruz
5084	Rochel Montero Lago
5085	Rodnei Bertazzoli
5086	Rodolfo do Couto Maia
5087	Rodolfo Eusebio Lagos Monaco Leighton
5088	Rodolfo Jardim de Azevedo
5089	Rodolpho Mattos Albano
5090	Rodolpho Vilhena de Moraes
5091	Rodrigo Affonseca Bressan
5092	Rodrigo Alexandre Panepucci
5093	Rodrigo Atanes Batistelo
5094	Rodrigo Augusto Ferreira de Souza
5095	Rodrigo Azevedo dos Reis
5096	Rodrigo Barbosa Capaz
5097	Rodrigo Bastos Fóscolo
5098	Rodrigo Correa de Oliveira
5099	Rodrigo Corrêa Diniz Peixoto

5100	Rodrigo de Almeida
5101	Rodrigo Facchini Magnani
5102	Rodrigo Fernando Bianchi
5103	Rodrigo Ghiringhelli de Azevedo
5104	Rodrigo Grassi-Oliveira
5105	Rodrigo Gribel Lacerda
5106	Rodrigo Guerino Stabeli
5107	Rodrigo Intini Marques
5108	Rodrigo Jesus de Medeiros
5109	Rodrigo Jose Bezerra de Siqueira
5110	Rodrigo Kerr Duarte Pereira
5111	Rodrigo Leonardo de Oliveira Basso
5112	Rodrigo Luiz Oliveira Rodrigues Cunha
5113	Rodrigo Nicolato
5114	Rodrigo Nicoletti
5115	Rodrigo Nunes da Fonseca
5116	Rodrigo Octavio Mendonça Alves de Souza
5117	Rodrigo Otávio Rodrigues de Melo Souza
5118	Rodrigo Prioli Menezes
5119	Rodrigo Rocha Latado
5120	Rodrigo Schütz Rodrigues
5121	Rodrigo Villares Portugal
5122	Rodrygo Luis Teodoro Santos
5123	Rogelio Lopes Brandao
5124	Rogemar André Riffel
5125	Roger Chammas
5126	Roger Frigério Castilho
5127	Rogéria Inês Rosa Lara
5128	Rogéria Rocha Gonçalves
5129	Rogério Antonio Freitag
5130	Rogério Campos
5131	Rogerio Correa
5132	Rogerio Dultra dos Santos
5133	Rogério Galante Negri
5134	Rogério Gastal Xavier
5135	Rogério Gribel
5136	Rogerio Hermida Quintella
5137	Rogerio Luis Maltez
5138	Rogerio Magalhães Paniago
5139	Rogério Mercandelle Santana
5140	Rogério Riffel
5141	Rogerio Rosenfeld
5142	Rogério Saad Hossne
5143	Rogerio Valaski
5144	Rogério Valentim Gelamo
5145	Rogério Vieira Rossi
5146	Roger Walz
5147	Rohit Gheyi
5148	Roland Ernst Vetter
5149	Roland Koberle
5150	Rolf Ribeiro de Souza
5151	Rolf Roland Weber
5152	Romário Cerqueira Leite
5153	Romario Gava Ferrao
5154	Romariz da Silva Barros
5155	Romelia Pinheiro Gonçalves
5156	Romero Tori
5157	Romildo Jeronimo Ramos
5158	Romulo Antonio Fuentes Flores
5159	Rômulo Augusto Ando

5160	Romulo Cerqueira Leite
5161	Rômulo José da Costa Ribeiro
5162	Romulo Simoes Angelica
5163	Ronald Buss de Souza
5164	Ronald Dickman
5165	Ronald Kennedy Luz
5166	Ronaldo Adelfo Wasum
5167	Ronaldo Alves Garcia
5168	Ronaldo Alves Pinto Nagem
5169	Ronaldo Arias
5170	Ronaldo Censi Faria
5171	Ronaldo da Silva Mohana Borges
5172	Ronaldo de Albuquerque Ribeiro
5173	Ronaldo Domingues Mansano
5174	Ronaldo Fiani
5175	Ronaldo Joaquim da Silveira Lobão
5176	Ronaldo Junio Campos Batista
5177	Ronaldo Mello Pereira
5178	Ronaldo Nascimento de Oliveira
5179	Ronaldo Nogueira de Moraes Pitombo
5180	Ronaldo Ramos Laranjeira
5181	Ronaldo Willian Reis
5182	Ronald Zanetti Bonetti Filho
5183	Ronei Jesus Poppi
5184	Ronei Marcos de Moraes
5185	Ronnie Antunes de Assis
5186	Roosecelis Brasil Martines
5187	Roque Pacheco de Almeida
5188	Rosa Helena Veras Mourão
5189	Rosalina Jorge Koifman
5190	Rosalinda Carmela Montone
5191	Rosa Maria de Medeiros Marinho
5192	Rosa Maria Meri Leão
5193	Rosa Maria Nascimento dos Santos
5194	Rosa Maria Videira de Figueiredo
5195	Rosana Aparecida Baeninger
5196	Rosana Aparecida Nogueira de Araujo
5197	Rosana Lopes Fialho
5198	Rosana Maria dos Reis
5199	Rosana Teresinha Vaccare Braga
5200	Rosane Gonçalves Ito
5201	Rosane Lowenthal
5202	Rosane Maria Trindade de Medeiros
5203	Rosane Minghim
5204	Rosane Rodrigues Chaves
5205	Rosane Silva
5206	Rosane Vianna Jorge
5207	Rosângela Aparecida Dellosso Penteado
5208	Rosângela Assis Jacques
5209	Rosângela de Oliveira Alves Carvalho
5210	Rosangela Getirana Santana
5211	Rosangela Gonçalves Peccinini
5212	Rosangela Saher Correa Cintra
5213	Rosario Dominguez Crespo Hirata
5214	Rose Adele Monteiro
5215	Roseane Borner de Oliveira
5216	Roseane Simões Palavizini
5217	Rosecelia Moreira da Silva Castro
5218	Rose Clívia Santos
5219	Roselane Laudares Silva

5220	Roselena Faez
5221	Roseli Buzanelli Torres
5222	Roseli Farias Melo de Barros
5223	Roseli Gedanke Shavitt
5224	Roseli Wassem
5225	Rosely Maria Zancopé Oliveira
5226	Rose Maria Carlos
5227	Rosemary Laís Galati
5228	Rosemary Matias
5229	Rosemary Vieira
5230	Rose Mary Zumstein Georgetto Naal
5231	Rosemeire Cristina Linhari Rodrigues Pietro
5232	Rosemery da Silva Nascimento
5233	Rosiane Lopes da Cunha
5234	Rosilene Fressatti Cardoso
5235	Rosilene Moretti Marçal
5236	Rosmeri Porfírio da Rocha
5237	Rossana Maria de Castro Andrade
5238	Rossana Pulcineli Vieira Francisco
5239	Rossiane Claudia Vommaro
5240	Roy Edward Bruns
5241	Rozangela Curi Pedrosa
5242	Rubem Carlos Araujo Guedes
5243	Ruben Dario Sinisterra Millán
5244	Rubens Cesar Lopes Figueira
5245	Rubens Duarte Coelho
5246	Rubens José Guimarães
5247	Rubens Maciel Filho
5248	Rubens Martins Moreira
5249	Rubens Monti
5250	Rubens Nascimento Melo
5251	Rubens Rodrigues dos Santos Junior
5252	Rubens Souza dos Santos
5253	Rubens Tadeu Delgado Duarte
5254	Rubens Tomio Honda
5255	Rubens Viana Ramos
5256	Rubia Figueredo Gouveia
5257	Rubiana Mara Mainardes
5258	Rudi Emerson de Lima Procópio
5259	Rudinei Goularte
5260	Rui Alberto Ferriani
5261	Rui Curi
5262	Rui Marcos Grombone de Vasconcellos
5263	Rusiene Monteiro de Almeida
5264	Russolina Benedeta Zingali
5265	Rute Loreto Sampaio de Oliveira
5266	Rute Maria Gonçalves de Andrade
5267	Ruth Meri Lucinda da Silva
5268	Ruy Carlos Ruver Beck
5269	Ruy Exel Filho
5270	Ruy Gastaldoni Jaeger
5271	Ruy José Válka Alves
5272	Ruy Kenji Papa de Kikuchi
5273	Ruy Luiz Milidiu
5274	Ruy Morgado de Castro
5275	Ruynet Lima de Matos Filho
5276	Ruy Silveira Moraes Filho
5277	Ruy Tojeiro de Figueiredo Junior
5278	Sabiniano Araujo Rodrigues
5279	Sacha Braun Chaves

5280	Sadek Crisostomo Absi Alfaro
5281	Said Najati Sidki
5282	Saint-Clair Cordeiro da Trindade Júnior
5283	Salomon Sylvain Mizrahi
5284	Salvador Airton Gaeta
5285	Salvador Pinillos Gimenez
5286	Samantha Monteiro Martins
5287	Sambasiva Rao Patchineelam
5288	Sâmia Andrícia Souza da Silva
5289	Samuel Aguiar Junior
5290	Samuel da Silva
5291	Samuel de Abreu Pessôa
5292	Samuel Goldenberg
5293	Samuel Hilsdorf Barbanti
5294	Samuell Aquino Holanda
5295	Samuel Leite de Oliveira
5296	Samuel Luna de Abreu
5297	Sanclayton Geraldo Carneiro Moreira
5298	Sandoval Carneiro Junior
5299	Sandra Andréa Santos da Silva
5300	Sandra Aparecida Drigo Linde
5301	Sandra Aparecida Santos
5302	Sandra Camargo Pinto Ferraz Fabbri
5303	Sandra Cristina Pereira Costa Fuchs
5304	Sandra de Brito Barreto
5305	Sandra de Souza Hacon
5306	Sandra Elisabete Vieira
5307	Sandra Estrazulas Farias
5308	Sandra Helena Poliselli Farsky
5309	Sandra Josefina Ferraz Ellero Grisi
5310	Sandra Maria Feliciano de Oliveira e Azevedo
5311	Sandra Maria Warumby Zanin
5312	Sandra Patricia Zanotto
5313	Sandra Regina Ceccato Antonini
5314	Sandra Regina Freitas Pinheiro
5315	Sandra Roberta Gouvea Ferreira Vivolo
5316	Sandra Sampaio Vianna
5317	Sandra Sueli Chan
5318	Sandra Verza da Silva
5319	Sandro Augusto Pavlik Haddad
5320	Sandro Gonçalves da Silva
5321	Sandro José de Souza
5322	Sandro Roberto Marana
5323	Sandro Roberto Valentini
5324	Sandro Rodrigues Mazorche
5325	Sandro Rogério de Almeida
5326	Sandro Schreiber de Oliveira
5327	Sandro Sendin Mitsuhiro
5328	Sânzia Alves do Nascimento
5329	Sarah Isabel Pinto Monteiro do Nascimento Alves
5330	Sára Maria Chalfoun de Souza
5331	Sara Teresinha Olalla Saad
5332	Sara Timoteo Passos
5333	Sarita Mazzini Bruschi
5334	Satie Taniguchi
5335	Saulo Finco
5336	Saulo Rabello Maciel de Barros
5337	Saulo Ribeiro de Freitas
5338	Sayonara Maria de Carvalho Gonzalez
5339	Sayuri Miyamoto

5340	Scott Correll Head
5341	Sean Wolfgand Matsui Siqueira
5342	Sebastián Gonçalves
5343	Sebastian Yuri Cavalcanti Catunda
5344	Sebastião André De Felice
5345	Sebastiao Antonio Loureiro de Souza e Silva
5346	Sebastiao Carlos Velasco e Cruz
5347	Sebastiao da Cruz Silva
5348	Sebastiao de Campos Valadares Filho
5349	Sebastião do Amaral Machado
5350	Sebastiao Gomes dos Santos Filho
5351	Sebastião José de Melo
5352	Sebastiao Jose Nascimento de Padua
5353	Sebastiao William da Silva
5354	Segundo Sacramento Urquiaga Caballero
5355	Selan Rodrigues dos Santos
5356	Selma Gomes Ferreira Leite
5357	Selma Junqueira
5358	Selma Maria Bezerra Jeronimo
5359	Selma Rodrigues de Castilho
5360	Selma Siéssere
5361	Selvino Neckel de Oliveira
5362	Sergio Akira Uyemura
5363	Sergio Alvaro de Souza Camargo Junior
5364	Sérgio André Fontes Azevedo
5365	Sergio Atala Dib
5366	Sergio Augusto de Miranda Chaves
5367	Sergio Bampi
5368	Sérgio Bergamaschi
5369	Sérgio Carlos Zilio
5370	Sergio Castelo Branco Soares
5371	Sergio Costa Oliveira
5372	Sérgio de Albuquerque
5373	Sergio de Almeida Oliveira
5374	Sergio de Azevedo
5375	Sergio de Mendonça
5376	Sergio Duvoisin Junior
5377	Sergio Eduardo de Carvalho Eyer Jorás
5378	Sergio Eduardo de Carvalho Machado
5379	Sérgio Faloni de Andrade
5380	Sergio Ferreira Juacaba
5381	Sergio Franca Adorno de Abreu
5382	Sergio Frascino Muller de Almeida
5383	Sergio Henrique Bezerra de Sousa Leal
5384	Sérgio Henrique Pezzin
5385	Sergio Herminio Brommonschenkel
5386	Sérgio Ivan Gil Braga
5387	Sergio Koifman
5388	Sergio Leonardo Gómez
5389	Sergio Luis Costa Ferreira
5390	Sérgio Luis Felisbino
5391	Sergio Luiz de Toledo Barreto
5392	Sergio Luiz Fontes
5393	Sérgio Machado Corrêa
5394	Sérgio Marsiglia Duailibi
5395	Sergio Medeiros Paulino de Carvalho
5396	Sergio Miranda Freire
5397	Sergio Monthezuma Santoianni Guerra
5398	Sergio Nascimento Duarte
5399	Sergio Novita Esteves

5400	Sérgio Oliveira De Paula
5401	Sergio Pacheco Neves
5402	Sergio Paulo Bydlowski
5403	Sérgio Pereira da Rocha
5404	Sergio Pereira Leite
5405	Sergio Pilling Guapyassu de Oliveira
5406	Sergio Pinheiro Firpo
5407	Sérgio Ricardo Bezerra dos Santos
5408	Sergio Ricardo de Lazaro
5409	Sergio Rodrigues Fontes
5410	Sergio Rosim
5411	Sergio Shiguemi Furuie
5412	Sergio Tadeu Bernatavicius
5413	Sergio Teixeira Ferreira
5414	Sérgio Telles de Oliva
5415	Sérgio Veloso Brant Pinheiro
5416	Sérgio Zucoloto
5417	Serguei Balachov
5418	Setsuo Iwakiri
5419	Severino Alves Junior
5420	Severino Collier Coutinho
5421	Shaker Chuck Farah
5422	Sharlene Lopes Pereira
5423	Sheila Bünecker Lecke
5424	Sheila Garcia
5425	Sheila Villanova Borba
5426	Sherlan Guimarães Lemos
5427	Shirlei Maria Recco-Pimentel
5428	Sidarta Tollendal Gomes Ribeiro
5429	Sidnei Antonio Pianaro
5430	Sidnei Joao Siqueira Sant'Anna
5431	Sidney Antonio da Silva
5432	Sidney Jose Lima Ribeiro
5433	Sidney Nicodemos da Silva
5434	Sidney Roberto Nadal
5435	Silene de Paulino Lozzi
5436	Silvana Allodi
5437	Silvana Amaral Kampel
5438	Silvana Auxiliadora Bordin da Silva
5439	Silvana Bocanegra
5440	Silvana Chiavegatto
5441	Silvana Cristina dos Santos
5442	Silvana dos Santos Meyrelles
5443	Silvana Maria Medeiros de Sousa Silva
5444	Silvana Maria Quintana
5445	Silvana Marques de Araujo
5446	Silvana Nair Leite
5447	Silvana Praxedes de Paiva Gurgel
5448	Silvana Quintella Cavalcanti Calheiros
5449	Silvana Spíndola de Miranda
5450	Silvane Maria Braga Santos
5451	Silvânia Maria Mendes de Vasconcelos
5452	Silvania Sousa do Nascimento
5453	Silvete Coradi Guerini
5454	Silvia Beatriz Boscardin
5455	Silvia Berlanga de Moraes Barros
5456	Silvia Carolina Guatimosim Fonseca
5457	Silvia Cristina Fernandes Rossi
5458	Silvia Ferreira Lima Cavalheiro
5459	Silvia Helena Paixao Alencar

5460	Sílvia Ligório Fialho
5461	Silvia Lima Costa
5462	Silvia Lorenz Martins
5463	Silvia Maranca
5464	Silvia Maria Giuliatti Winter
5465	Silvia Maria Gomes Massironi
5466	Silvia Passos Andrade
5467	Silvia Regina Batistuzzo de Medeiros
5468	Silvia Regina Cavani Jorge Santos
5469	Silvia Regina Rogatto
5470	Sílvia Stanisçuaski Guterres
5471	Silvia Storpirtis
5472	Silvio Alexandre Beisl Vieira de Melo
5473	Silvio Barberato Filho
5474	Silvio Bispo do Vale
5475	Silvio Brienza Júnior
5476	Sílvio Carlos Ribeiro Vieira Lima
5477	Silvio Coelho dos Santos
5478	Silvio do Desterro Cunha
5479	Silvio Ernesto Barbin
5480	Silvio Frosini de Barros Ferraz
5481	Sílvio José Reis da Silva
5482	Silvio Nilo Figueroa Rivero
5483	Silvio Rainho Teixeira
5484	Silvio Romero de Lemos Meira
5485	Silvya Stuchi Maria-Engler
5486	Simaia do Socorro Sales das Mercês
5487	Simara Marcia Marcato
5488	Simey Thury Vieira Fisch
5489	Simone Caldas Tavares Mafra
5490	Simone Cecilio Hallak Regalo
5491	Simone Cristina Baggio Gnoatto
5492	Simone Cristina Olenscki Gilli
5493	Simone da Graca Ribeiro
5494	Simone Dantas de Souza
5495	Simone de Lima Martins
5496	Simone Diniz Junqueira Barbosa
5497	Simone do Rocio Senger de Souza
5498	Simone Eliza Facioni Guimaraes
5499	Simone Gonçalves Cardoso
5500	Simone Karst Passos Soares
5501	Simone Kashima Haddad
5502	Simone Kodlulovich
5503	Simone Koprowski Garcia
5504	Simone Maria Gonçalves de Barros
5505	Simone Martins Mendes
5506	Simone Pereira da Costa Dourado
5507	Simone Pereira da Silva Ribeiro
5508	Simone Sette Lopes Lafayette
5509	Simone Silva Alexandre
5510	Simoni Lahud Guedes
5511	Simoni Margareti Plentz Meneghetti
5512	Sinésio Talhari
5513	Siome Klein Goldenstein
5514	Sirlei Daffre
5515	Sivanildo da Silva Borges
5516	Sócrates Cabral de Holanda Cavalcanti
5517	Socrates de Oliveira Dantas
5518	Solange Binotto Fagan
5519	Solange Cadore

5520	Solange Maria de Toledo Serrano
5521	Solange Reis Ferreira
5522	Sol Garson Braule Pinto
5523	Songeli Menezes Freire
5524	Song Won Park
5525	Sônia Cristina Lima Chaves
5526	Sonia Isoyama Venancio
5527	Sônia Letícia de Méllo Cardoso
5528	Sonia Licia Baldochi
5529	Sônia Lúcia Modesto Zampieron
5530	Sonia Maria Barreto Pereira
5531	Sonia Maria Dozzi Brucki
5532	Sonia Maria Flores Gianesella
5533	Sonia Maria Malmonge
5534	Sônia Nair Báo
5535	Sonia Regina da Cal Seixas
5536	Sônia Renaux Wanderley Louro
5537	Sônia Weidner Maluf
5538	Soniza Vieira Alves-Leon
5539	Sorahia Domenice
5540	Soraia Carvalho de Souza
5541	Soraia Maria do Socorro Carlos Vidal
5542	Soraia Raupp Musse
5543	Soraia Vanessa Matarazzo
5544	Soraya Maria Vargas Cortes
5545	Soraya Silveira Simões
5546	Stanislav Moshkalev
5547	Steel Silva Vasconcelos
5548	Stefane Rodrigues Xavier Lopes
5549	Stefan Michael Blawid
5550	Stefan Schwab
5551	Stela Maris Kuze Rates
5552	Stella Maris da Silveira Duarte
5553	Stenio Perdigão Fragoso
5554	Stephan Stephany
5555	Stephen Patrick Walborn
5556	Stevens Kastrup Rehen
5557	Sttela Dellyzete Veiga Franco da Rosa
5558	Suédina Maria de Lima Silva
5559	Sueli Rodrigues
5560	Suely Lins Galdino
5561	Suely Vilela Sampaio
5562	Su Jian
5563	Sulamita Klein
5564	Suraia Said
5565	Susana de Souza Lalic
5566	Susana Ines Cordoba de Torresi
5567	Susana Maria Werner Samuel
5568	Susana Marta Isay Saad
5569	Susana Tchernin Wofchuk
5570	Susan Martins Pereira
5571	Susanne Rath
5572	Suzana Assad Kahn
5573	Suzana Caetano da Silva Lannes
5574	Suzana Carvalho Herculano Houzel
5575	Suzana Guimaraes Leitao
5576	Suzana Maria de Salis
5577	Suzana Maria Gico Lima Montenegro
5578	Suzana Pasternak
5579	Suzete Araujo Oliveira Gomes

5580	Suzete Maria Cerutti
5581	Suzi Alves Camey
5582	Sylvia Mendes Carneiro
5583	Sylvio Carlos Bandeira de Mello e Silva
5584	Sylvio Ferraz Mello
5585	Sylvio Luiz Mantelli Neto
5586	Sylvio Roberto Accioly Canuto
5587	Symone Fulgencio Lima
5588	Tadeu da Mata Medeiros Branco
5589	Tadeu Uggere de Andrade
5590	Taisy Silva Weber
5591	Takako Matsumura Tundisi
5592	Takashi Yoneyama
5593	Talita Mazon
5594	Tamar Gomes Pinheiro Frankenfeld
5595	Tânia Alves Amador
5596	Tania Aparecida Silva Brito
5597	Tania Bacelar de Araujo
5598	Tania Beatriz Creczynski Pasa
5599	Tania Cristina Leite de Sampaio e Spohr
5600	Tânia Fraga Barros
5601	Tania Marcourakis
5602	Tânia Maria de Almeida Alves
5603	Tania Maria Sarmento da Silva
5604	Tania Mari Bellé Bresolin
5605	Tânia Pereira Dominici
5606	Tania Tome Martins de Castro
5607	Tania Ueda Nakamura
5608	Tarlei Arriel Botrel
5609	Tatiana Alexandrovna Michtchenko
5610	Tatiana Barichello
5611	Tatiana de Almeida Simão
5612	Tatiana de Campos Bicudo
5613	Tatiana Menchini Steiner
5614	Tatiana Paula Teixeira Ferreira
5615	Tatiane Almeida de Menezes
5616	Tatiane Corrêa de Godoy
5617	Tatiane da Silva Dal-Pizzol
5618	Tatiane Pereira de Souza
5619	Tayana Uchôa Conte
5620	Tecia Maria Ulisses de Carvalho
5621	Telma Teresinha Berchielli
5622	Teodoro Isnard Ribeiro de Almeida
5623	Teogenes Augusto da Silva
5624	Tercilio Calsa Junior
5625	Tercio Ambrizzi
5626	Teresa Cristina Bezerra Saldanha
5627	Teresa Cristina de Abreu Ferrari
5628	Teresa Cristina Sauer de Avila-Pires
5629	Teresa Cristina Tavares Dalla Costa
5630	Teresa de Souza Fernandez Seixas
5631	Teresa Dib Zambon Atvars
5632	Teresa Domitila Fossari
5633	Teresa Maria de Jesus Ponte Carvalho
5634	Teresa Maria Fernandes de Freitas Mendes
5635	Teresinha de Jesus Alvarenga Rodrigues
5636	Teresinha de Jesus Stuchi
5637	Teresinha Gonçalves da Silva
5638	Tereza Cristina Melo de Brito Carvalho
5639	Tereza Maria Dantas de Medeiros

5640	Tereza Maria Piccinini Feitosa
5641	Terezinha Inez Estivalet Svidzinski
5642	Tetsuo Yamane
5643	Thaciana Valentina Malaspina Fileti
5644	Thadeu Josino Pereira Penna
5645	Thaiana da Cunha Ferreira Mendes
5646	Thais Aidar de Freitas Mathias
5647	Thaisa Storchi Bergmann
5648	Thais Cristina Baeta Soares Souto Padron
5649	Thais Horta Álvares da Silva
5650	Thais Mauad
5651	Thais Mothé Diniz
5652	Thais Navajas Corbisier
5653	Thais Porlan de Oliveira
5654	Thea Mirian Medeiros Machado
5655	Theodomiro Dias Neto
5656	Theodoro Antoun Netto
5657	Theophilos Rifiotis
5658	Theresinha Monteiro Absher
5659	Thereza Amélia Soares da Silva
5660	Thereza Christina Barja Fidalgo
5661	Thereza Christina Vessoni Penna
5662	Thereza Cristina Cardoso Menezes
5663	Thereza Cristina de Lacerda Paiva
5664	Thêssa Cristina Alonso
5665	Thiago Fernandes Bernardes
5666	Thiago José de Carvalho André
5667	Thiago Lima da Silva
5668	Thiago Mattar Cunha
5669	Thiago Mendonça de Aquino
5670	Thiago Motta Venancio
5671	Thiago Rodrigues de Oliveira
5672	Thiago Sotero Fragoso
5673	Thomas Dan Otto
5674	Thomas Patrick Dwyer
5675	Thomas Scheller
5676	Tiago Antônio da Silva Brandão
5677	Tiago Francisco Manea
5678	Tiago Góss dos Santos
5679	Tiago Lima Massoni
5680	Tiago Roberto Balen
5681	Tiago Venancio
5682	Ticiano José Saraiva dos Santos
5683	Tina Bimestre Selles Ribeiro
5684	Tito Dias Júnior
5685	Tito José Bonagamba
5686	Toby Alan Gardner
5687	Tomas Hrbek
5688	Tomaz Henrique de Araujo
5689	Tomomasa Yano
5690	Tonny José Araújo da Silva
5691	Túlio Flávio Accioly de Lima e Moura
5692	Tullo Vigevani
5693	Turan Peter Urmenyi
5694	Turibio Gomes Soares Neto
5695	Ubirajara Agero Batista
5696	Ubiratan de Paula Santos
5697	Ubiratan Fabres Machado
5698	Uilson Schwantz Sias
5699	Uira Kulesza

5700	Ulf Mehlig
5701	Ulisses Franz Bremer
5702	Ulisses Gazos Lopes
5703	Ulrich Christian Karl Heinz Bruno Seeliger
5704	Ulysses Garcia Casado Lins
5705	Umberto Giuseppe Cordani
5706	Umberto Klock
5707	Umberto Laino Fulco
5708	Uwe Horst Schulz
5709	Vagner Anabor
5710	Vagner Candido de Sousa
5711	Vagner de Castro
5712	Valbert Nascimento Cardoso
5713	Valdair Francisco Muglia
5714	Valdeilson Souza Braga
5715	Valdely Ferreira Kinupp
5716	Valdemir Garcia Ferreira
5717	Valdenice Moreira Novelli
5718	Valderes Drago
5719	Valder Nogueira Freire
5720	Valder Steffen Junior
5721	Valdiléa Gonçalves Veloso dos Santos
5722	Valdinete Lins da Silva
5723	Valdir Barbosa Bezerra
5724	Valdir Florencio da Veiga Junior
5725	Valdir Grassi Junior
5726	Valdir Marcos Stefenon
5727	Valdir Soldi
5728	Valdivino Alexandre de Santiago Júnior
5729	Valdo da Silva Marques
5730	Valdo Jose Dias da Silva
5731	Valentina Porta
5732	Valentin Obac Roda
5733	Valeria Belli Riatto
5734	Valeria Cavalcanti Rolla
5735	Valéria de Oliveira
5736	Valeria Gonçalves da Vinha
5737	Valéria Gonçalves Soares
5738	Valeria Maria de Souza Framil
5739	Valéria Maria Nascimento Abreu
5740	Valéria Moraes Longo
5741	Valeria Pereira de Sousa
5742	Valéria Regina de Souza Moraes
5743	Valéria Rêgo Alves Pereira
5744	Valéria Saldanha Motta
5745	Valerio Carruba
5746	Valfredo Azevedo Lemos
5747	Valmecir Antonio dos Santos Bayer
5748	Valmir Antonio Costa
5749	Valmir Carneiro Barbosa
5750	Valmir da Silva Souza
5751	Valmor Roberto Mastelaro
5752	Valnês da Silva Rodrigues Junior
5753	Valquiria de Campos
5754	Valquiria Linck Bassani
5755	Valtencir Zucolotto
5756	Valter Vieira de Camargo
5757	Vanda Porpino Lemos
5758	Vandeir Francisco Guimaraes
5759	Vander de Freitas Melo

5760	Vanderlan da Silva Bolzani
5761	Vanderlei Cunha Parro
5762	Vanderlei Gageiro Machado
5763	Vanderlei Perez Canhos
5764	Vanderlei Salvador Bagnato
5765	Vanessa Carla Furtado Mosqueira
5766	Vanessa Cristina Arantes
5767	Vanessa de Paula Soares Rachetti
5768	Vanessa Hatje
5769	Vanessa Petrilli Bavaresco
5770	Vanessa Pinho da Silva
5771	Vanessa Przybylski Ribeiro Magri
5772	Vanessa Schmidt Giacomelli
5773	Vania Aparecida Silva
5774	Vânia Caldas de Sousa
5775	Vania dos Santos
5776	Vania Ferreira Prado
5777	Vânia Gomes Zuin
5778	Vania Lucia Loro
5779	Vânia Maria Corrêa da Costa
5780	Vânia Maria Maciel Melo
5781	Vania Passarini Takahashi
5782	Vânia Rita Elias Pinheiro Bittencourt
5783	Vânia Zikán Cardoso
5784	Venerando Eustáquio Amaro
5785	Vera Aparecida Fernandes Martin
5786	Vera Fernanda Martins Hossepian de Lima
5787	Vera Jatenco Silva Pereira
5788	Vera Lucia Antunes de Lima
5789	Vera Lúcia Azzolin Frescura Bascuñan
5790	Vera Lucia Borges Isaac
5791	Vera Lúcia de Miranda Guarda
5792	Vera Lucia Divan Baldani
5793	Vera Lucia Doyle Louzada de Mattos Dodebei
5794	Vera Lucia Eifler-Lima
5795	Vera Lucia Flor Silveira
5796	Vera Lucia Lanchote
5797	Vera Lucia Michalany Chaia
5798	Vera Lucia Rodrigues Machado Benassi
5799	Vera Margarete Scarpassa
5800	Vera Maria Ferreira da Silva
5801	Vera Maria Fonseca de Almeida e Val
5802	Verônica Maria Morandi da Silva
5803	Veronica Massena Reis
5804	Verônica Porto Carreiro de Vasconcellos Coelho
5805	Vicente de Paulo Teixeira Pinto
5806	Vicente Gomes
5807	Vicente Lopes Junior
5808	Victor Bertucci Neto
5809	Victor Ciro Solano Reynoso
5810	Victor Hugo Aquino Quintana
5811	Victor Pellegrini Mammana
5812	Victor Prochnik
5813	Victor Sonnenberg
5814	Victor Túlio Ribeiro de Resende
5815	Vietla Satyanarayana Rao
5816	Vilma de Lima
5817	Vilma Mota da Silva
5818	Vilma Regina Martins
5819	Vincent Patrick Marie Bourguet

5820	Vinicius André Rodrigues Henriques
5821	Vinícius Antonio de Oliveira Dittrich
5822	Vinicius Bordalo Schmidt Marques
5823	Vinicius de Frias Carvalho
5824	Vinicius de Souza Cantarelli
5825	Vinícius Piccirillo
5826	Virgilio Augusto Fernandes Almeida
5827	Virginia Berlanga Campos Junqueira
5828	Virginia Maria Tavano
5829	Virginia Mello Alves
5830	Virginia Ragoni de Moraes Correia
5831	Virginia Sampaio Teixeira Ciminelli
5832	Virgínia Verônica de Lima
5833	Vital Pedro da Silva Paz
5834	Vitaly Félix Rodríguez Esquerre
5835	Vitor Francisco Ferreira
5836	Vitor Hugo Ferreira
5837	Vitor Rafael Coluci
5838	Vitor Tumas
5839	Vitor Vivolo
5840	Vivaldo Moura Neto
5841	Viviana Giampaoli
5842	Viviane Monteiro Góes
5843	Viviane Pereira Moreira
5844	Viviane Renata Scalon
5845	Viviane Santuari Parisotto Marino
5846	Viviane Torres da Silva
5847	Vivian Gilbert Ferreira Paes
5848	Vivian Mary Barral Dodd Rumjanek
5849	Vívian Nicolau Gonçalves
5850	Vladas Sidoravicius
5851	Vladimir Jesus Trava-Airoldi
5852	Vladimir Pinheiro Ponczek
5853	Volnei Tita
5854	Volney de Magalhaes Camara
5855	Wagner da Silva Amaral
5856	Wagner de Oliveira Vital
5857	Wagner Flauber Araujo Lima
5858	Wagner José Corradi Barbosa
5859	Wagner Luiz Ferreira Marcolino
5860	Wagner Luiz Ramos Barbosa
5861	Wagner Meira Junior
5862	Wagner Nunes Rodrigues
5863	Walace Gomes Leal
5864	Walcy Santos
5865	Waldeci Paraguassu Feio
5866	Waldemar de Castro Leite Filho
5867	Waldemar Silva Costa
5868	Walderez Ornelas Dutra
5869	Waldinei Rosa Monteiro
5870	Waldir Avansi Junior
5871	Waldo Gonzalo Cancino Ticona
5872	Waleska Martins Eloi
5873	Walkyria Bueno Scivittaro
5874	Wallace Chamon Alves de Siqueira
5875	Wallace Duarte Fragoso
5876	Wallace Pacienza Lima
5877	Walmir Maximo Torres
5878	Walnéia Aparecida de Souza
5879	Walter Antônio Pereira Abrahão

5880	Walter Araujo Zin
5881	Walter Collischonn
5882	Walter de Britto Vidal Filho
5883	Walter Demetrio Gonzalez Alarcon
5884	Walter dos Santos Soares Filho
5885	Walter Eugênio de Medeiros
5886	Walter José Rodrigues Matrangolo
5887	Walter Junqueira Maciel
5888	Walter Katsumi Sakamoto
5889	Walter Martin Roland Oelemann
5890	Walter Mendes de Azevedo
5891	Walter Ribeiro Terra
5892	Wanda Maria Almeida von Kruger
5893	Wandemberg Paiva Ferreira
5894	Wanderley Dantas dos Santos
5895	Wanderley de Souza
5896	Wanderley Ferreira de Amorim Júnior
5897	Wanderley Lopes de Souza
5898	Wanderley Pereira Oliveira
5899	Wanderley Rodrigues Bastos
5900	Wanderli Pedro Tadei
5901	Wander Luiz Vasconcelos
5902	Wanderson Duarte da Rocha
5903	Wânia Pasinato
5904	Warlley de Sousa Sales
5905	Washington Luis Conrado dos Santos
5906	Washington Luiz Assuncao Pereira
5907	Washington Marcondes Ferreira Neto
5908	Watson Loh
5909	Wayner Vieira de Souza
5910	Weber Friederichs Landim de Souza
5911	Welington Celso de Melo
5912	Welles Antonio Martinez Morgado
5913	Wellington Avelino do Amaral
5914	Wellington da Silva Mendes
5915	Wellington de Paula Martins
5916	Wellington Ferreira da Silva Filho
5917	Wellington Garcia Campos
5918	Wendel Andrade Alves
5919	Wendell Karlos Tomazelli Coltro
5920	Wesley Bueno Cardoso
5921	Widinei Alves Fernandes
5922	Wido Herwig Schreiner
5923	Wilfried Klein
5924	Wilhelm Passarella Freire
5925	Wilhelmus Adrianus Maria Van Noije
5926	Wilker Ribeiro Filho
5927	William Augusto Rodrigues de Souza
5928	William Dias Belangero
5929	William Ernest Magnusson
5930	William Eufrasio Nunes Pereira
5931	Willian Ricardo Rocha
5932	Willy Alvarenga Lacerda
5933	Wilmar Barbosa Ferraz
5934	Wilma Regina Barrionuevo
5935	Wilson Alves do Prado
5936	Wilson Araújo da Silva Junior
5937	Wilson Araújo Lopes
5938	Wilson da Costa Santos
5939	Wilson de Figueiredo Jardim

5940	Wilson Jacob Filho
5941	Wilson Magela Gonçalves
5942	Wilson Marques Junior
5943	Wilson Moreira Dutra Junior
5944	Wilson Negrão Macêdo
5945	Wilson Ricardo Matos Rabelo
5946	Wilson Roberto Malfará
5947	Wilson Roberto Spironello
5948	Wilson Sacchi Peternele
5949	Wilson Teixeira
5950	Wilton da Silva Dias
5951	Wim Maurits Sylvain Degrave
5952	Wladimyr Mattos da Costa Dourado
5953	Wolfgang Johannes Junk
5954	Yan Levin
5955	Yara Maria Lucisano Valim
5956	Ygor Arzeno Ferrão
5957	Yocie Yoneshigue Valentin
5958	Yoshiharu Kohayakawa
5959	Yoshio Kawano
5960	Yoshitaka Gushikem
5961	Younes Messaddeq
5962	Yraima Moura Lopes Cordeiro
5963	Yuan Jinyun
5964	Yvonne Primerano Mascarenhas
5965	Yzel Rondon Súarez
5966	Zelia Ines Portela Lobato
5967	Zélia Soares Macedo
5968	Zoroastro Torres Vilar
5969	Zuleica Bruno Fortes
5970	Zulema Abraham
5971	Zulma das Graças Lucena Schussel
5972	Zulmira Aurea Cruz Bomfim
5973	Zulmira Guerrero Marques Lacava
